# Unveiling the bactericidal effects of extracts and phytocompounds from *Eichhornia crassipes* (Mart.) Solms against methicillin-resistant *Staphylococcus aureus* (MRSA): An *in vitro* and *in silico* approach

**DOI:** 10.1371/journal.pone.0349750

**Published:** 2026-06-11

**Authors:** Md. Mashiar Rahman, Shaulina Sayeed Simu, Nafis Fuad Shahir, Sadia Israt, Nazia Islam Rafi, Md. Enamul Kabir Talukder, Md. Rashidur Rahman, Mohammad Abu Hena Mostofa Jamal, Shahina Akhter

**Affiliations:** 1 Molecular and Cellular Biology Laboratory (MCBL), Department of Genetic Engineering and Biotechnology, Jashore University of Science and Technology, Jashore, Bangladesh; 2 Department of Pharmacy, Jashore University of Science and Technology, Jashore, Bangladesh; 3 Department of Biotechnology and Genetic Engineering, Islamic University, Kushtia, Bangladesh; 4 Department of Life Sciences, School of Environment and Life Sciences, Independent University, Bangladesh (IUB), Bashundhara R/A, Dhaka, Bangladesh; University of Buea, CAMEROON

## Abstract

**Background:**

*Eichhornia crassipes* (Mart.) Solms is used as a vegetable by the tribal communities of the Chittagong Hill Tracts in Bangladesh, where it has traditionally been employed to treat diarrhea, intestinal worms, digestive disorders, and hepatic conditions. However, its effects against methicillin-resistant *Staphylococcus aureus* (MRSA) remain unexplored.

**Purpose:**

To assess the *in vitro* antibacterial potential of methanol, ethanol, and ethyl acetate extracts from the flowers and leaves of *E. crassipes* (MEECF, EEECF, EAEECF, MEECL, EEECL, and EAEECL) and to identify multi-target antibacterial phytocompounds *in silico* against MRSA.

**Methods:**

Preliminary phytochemical screening (PPS), Fourier-transform infrared spectroscopy (FT-IR), and gas chromatography-mass spectrometry (GC-MS) were used for phytochemical profiling of MEECF, EEECF, EAEECF, MEECL, EEECL, and EAEECL. The antimicrobial activity of the extracts was evaluated using *in vitro* agar well diffusion, minimum inhibitory concentration (MIC), and minimum bactericidal concentration (MBC) assays. Computational approaches were applied to identify multi-targeting agents from GC-MS-annotated phytocompounds.

**Results:**

In phytochemical profiling, PPS revealed the diverse nature of the plant’s compounds, while FT-IR identified various functional groups. GC-MS analysis metabolically annotated a total of 383 phytocompounds across all extracts. Molecular docking identified two key compounds: CID 4970 in MEECF and EEECF, and CID 615944 in MEECL. These hit phytocompounds exhibited strong multi-modal binding affinity toward β-lactamase and PBP2a. Further analysis demonstrated that CID 4970 and CID 615944 possessed favorable pharmacokinetic (PK) and drug-like properties without toxicity, making them promising candidates. Molecular dynamics simulation confirmed the binding stability and multi-modal inhibitory activity of CID 4970 and CID 615944 within the active sites of β-lactamase and PBP2a, reinforcing their potential as lead compounds for developing inhibitors against these proteins. All extracts demonstrated dose-dependent antibacterial activity in agar well diffusion, MIC, and MBC assays, supporting the *in silico* results.

**Conclusion:**

This study provides scientific validation for the traditional use of *E. crassipes* in treating bacterial infections and suggests that CID 4970 and CID 615944 could serve as promising candidates for developing novel anti-MRSA agents.

## 1. Introduction

Infectious diseases are the second most frequent cause of fatality worldwide [1]. *Staphylococcus aureus* (*S. aureus*) is a widespread human pathogen associated with numerous infectious conditions, including dermal and subdermal infections, infective endocarditis, bone infection, bloodstream infection, and severe pneumonia. Based on its antibiotic susceptibility, *S. aureus* is categorized into methicillin-sensitive *S. aureus* (MSSA) and methicillin-resistant *S. aureus* (MRSA). MRSA, in particular, has evolved into a major contributor to infections occurring in both community and hospital settings. In recent decades, bacterial adaptation along with the excessive use of antibiotics has contributed to the steady rise of drug resistance in *S. aureus*, and MRSA infections have increased worldwide. Studies indicate that MRSA is responsible for infections at a rate ten times higher than that of all multidrug-resistant (MDR) Gram-negative pathogens combined. Currently, MRSA has been designated by the WHO as one of the twelve critical pathogens posing a threat to human health, making anti-infective treatment increasingly challenging [2]. MRSA’s resistance evolves rapidly through several mechanisms, including (i) inactivation of β-lactam antibiotics by β-lactamase, (ii) expression of an alternative transpeptidase, PBP2a, instead of penicillin-binding proteins (PBPs), (iii) horizontal gene transfer, (iv) chromosomal mutations, (v) traditional resistance mechanisms, and (vi) the capacity to persist on both living and nonliving surfaces through the development of biofilms [1,2].

A growing body of research has shown that MRSA develops resistance against β-lactam antibiotics (penicillins, cephalosporins, cephamycins, monobactams) through the synthesis of the β-lactamase enzyme. This enzyme breaks down the four-membered β-lactam ring in these antibiotics, thereby deactivating their antibacterial properties [1–3]. A significant quantity of β-lactamase rapidly binds and securely attaches to extracellular antibiotics, blocking them from entering the intracellular space. As a result, MRSA develops antibiotic resistance due to the drugs’ inability to reach their target site.

Generally, β-lactam antibiotics interfere with bacterial cell wall construction by binding to PBPs, which are membrane-associated enzymes involved in cross-linking peptidoglycan strands through transpeptidation, a crucial step in maintaining the structural integrity of the bacterial cell wall [2,4]. However, MRSA expresses a modified version of PBPs (PBP2a), encoded within the *mecA* gene, that spreads via horizontal gene transfer [5]. This modification prevents antibiotics from binding to PBP2a, resulting in antibiotic resistance [5]. Despite the modification, PBP2a retains the structural features of PBPs, blocking the β-lactam antibiotic binding site while facilitating the enzymatic reactions that connect peptidoglycan strands in the bacterial cell wall [2,4].

Both antibiotic resistance mechanisms of MRSA limit available treatments, prompting researchers to seek novel antibacterial agents effective against MRSA. Compounds with multiple targets that exhibit efficacy against multiple biological targets have gained significant importance in drug discovery due to the emergence of diseases through multiple mechanisms [6,7]. In this context, a multi-target drug that counteracts several antibiotic resistance mechanisms of MRSA would logically be the best choice for combating the infection. Therefore, a single-compound, multi-targeting approach that inhibits both β-lactamase and PBP2a could be a promising strategy for developing a drug against MRSA. Plant products have drawn increasing attention from researchers in the search for alternative and novel antibacterial drugs due to their low or no side effects, safety, efficacy, cost-effectiveness, structural diversity, and multitarget functionality.

*Eichhornia crassipes* (Mart.) Solms is classified under the kingdom Plantae, within the class Liliopsida, order Commelinales, family Pontederiaceae, and belongs to the genus *Eichhornia* and the species *Eichhornia crassipes*. It is commonly known as water hyacinth, a freely floating, perennial hydrophytic plant found across Bangladesh. Within the genus, *E. crassipes* represents one of the most morphologically distinct and widely studied species due to its invasive behavior and rapid vegetative growth. This species is recognized by its bulbous petioles, lavender flowers with a prominent eye spot on the upper petal, and a fibrous root system. It is used as a carotene-rich vegetable in Vietnam, Japan, Taiwan, Thailand, and among the tribal communities of the Chittagong Hill Tracts in Bangladesh. Various parts of *E. crassipes* have traditionally been utilized as herbal remedies due to their therapeutic effects on human ailments. The plant manages gastrointestinal conditions, including diarrhea, intestinal parasites, digestive disturbance, and bloating. Additionally, its seeds are used to promote healthy spleen function [8]. In Bangladesh, the roots and flowers have been traditionally used to treat hepatic disorders and abdominal swelling [9]. The water extract derived from *E. crassipes* leaves has demonstrated potent antimicrobial effects against *Proteus vulgaris*, *Salmonella typhi,* and *Bordetella bronchiseptica* [10]. The crude extract, along with its isolated fractions from *E. crassipes,* has shown antibacterial activity against *Enterococcus faecalis* and *Escherichia coli*, as well as remarkable antifungal activity against *Aspergillus flavus*, *Aspergillus niger*, and *Candida albicans* [11]. Additionally, the antimicrobial potential of extracts prepared using hydro alcohol and ethanol from *E. crassipes* has also been extensively studied against *Bacillus subtilis*, *Escherichia coli*, *Staphylococcus epidermidis*, and *Pseudomonas aeruginosa* [12]. Moreover, the disc diffusion method has evaluated the antifungal effects of ethanol extracts from the shoots and leaves against *Aspergillus fumigatus* and *Monascus ruber* [13]. It has also been reported that *E. crassipes* is an abundant source of diverse secondary metabolites with various pharmacological properties, including antiviral, antifungal, antitumor, antibacterial, skin-whitening, neuroprotective, and hepatoprotective effects [13].

Although the phytochemical composition of *E. crassipes* has been extensively studied, its potential activity specifically against MRSA and the molecular mechanisms underlying this activity remain largely unexplored. More importantly, no existing research has evaluated whether its phytochemicals can effectively target and inhibit the two key MRSA resistance determinants, β-lactamase and PBP2a, through a multi-targeted computational approach. To date, the pharmacokinetics, drug-likeness, toxicity profiling, and molecular dynamics–based validation of *E. crassipes*-derived phytochemicals against MRSA have not been systematically investigated.

Therefore, the present study aims to confirm the major functional groups and compounds present in the extracts using FT-IR and GC-MS, drawing on previously documented chemical profiles; evaluate the antibacterial activity of leaf and flower extracts against MRSA; identify potentially active phytochemicals through molecular docking; assess the pharmacokinetic, drug-likeness, and toxicity characteristics of the top-ranked compounds; and validate their inhibitory effects on β-lactamase and PBP2a through molecular dynamics simulation. This integrated approach is intended to identify promising lead compounds from *E. crassipes* with multi-targeting potential for combating MRSA infections.

## 2. Materials and methods

### 2.1. Solvents and chemicals

Methanol (Cat# 179337, ACS reagent), ethanol (Cat# 459844, ACS reagent) and ammonia solution (Cat#221228, ACS reagent), Fehling’s solutions A (Cat# 11–0090, SAJ special grade) and Fehling’s solutions B (Cat# 11–0100, SAJ special grade), CuSO₄ (Cat# C1297), ethyl acetate (Cat# 319902, ACS reagent), hydrochloric acid (Cat# 22020198, Analytical grade), sulfuric acid (Cat# 1.12080, Analytical grade), glacial acetic acid (Cat# A6283, Analytical grade), chloroform (Cat# 319988, Analytical grade), and ferric sulfate (Cat# F0638, Analytical grade), lead acetate (Cat# 215902, ACS reagent), ferric chloride (Cat#157740, Reagent grade), sodium chloride (Cat# 05819, ACS reagent), sodium nitroprusside (Cat#71778, ACS reagent), pyridine (Cat#33553, ACS reagent), tris base (Cat# T6687), ninhydrin (Cat# 151173, ACS reagent) potassium dihydrogen phosphate (Cat# P0662, ACS reagent), potassium persulfate (Cat# 216224, ACS reagent), Na₂HPO₄ (Cat# S9763, ACS reagent) and monosodium dihydrogen phosphate (Cat# S9638, ACS reagent), ammonia solution (Cat# 221228, ACS reagent), bacto-agar (Cat# A5306, molecular biology grade), Nutrient agar (Cat # 17222, Cell culture grade), and nutrient broth (Cat# 03856, Cell culture grade) were purchased from Sigma-Aldrich, Germany. Sodium hydroxide (Cat#05898, Extra pure), Mayer’s reagent (Cat# 04537, Extra pure), and potassium chloride (Cat# 05339, Extra pure) were obtained from BIOSOL, India. Methicillin antibiotic discs were procured from HiMedia, India.

### 2.2. Plant material collection

The flowers, along with the leaves, of *E. crassipes* were harvested in February 2023 from their natural habitats around the campus of Jashore University of Science and Technology (JUST), Jashore, Bangladesh, and were taxonomically verified and identified by Dr. A. H. M. Mahbubur Rahman, Professor in the Department of Botany at the University of Rajshahi, Bangladesh. The specimen was preserved there under the voucher number RUHLF248, ensuring traceability and reproducibility of the study. The gathered plant materials were rinsed under running tap water and subsequently dried at room temperature (25°C) in an air-conditioned space. Once dried, the plant components were finely ground into powder using an electric grinder and stored at 4°C in sealed containers.

### 2.3. Plant extracts preparation

Plant leaves and flowers were extracted according to previously described methods, with slight alterations [14]. In summary, six flasks were used in total, of which three contained 25 g of leaf powder each and the remaining three contained 25 g of flower powder each. In the leaf powder group, 100 mL of methanol, ethanol, and ethyl acetate were separately added to the first, second, and third flasks, respectively. Similarly, in the flower powder group, 100 mL of methanol, ethanol, and ethyl acetate were separately added to the first, second, and third flasks, respectively.. The flasks were placed in a shaking incubator (JSSI300T, JSR, South Korea) set to 250 rpm and maintained at 37 °C for 72 hours. After incubation, the mixtures were first passed through cotton gauze and subsequently through Whatman no. 1 filter paper. The filtrates were then reduced in volume under vacuum at room temperature with the help of a rotary evaporator (DLAB Scientific Inc., CA, USA), yielding concentrated extracts of the flowers and leaves. From 25 g of powdered plant material, 2.5 to 2.75 g of crude extract was obtained, equivalent to 10% to 11% yield by dry weight. The concentrated extracts were stored in airtight containers at 4°C until further analysis.

### 2.4. Bacterial strain collection

A glycerol stock containing methicillin-resistant *Staphylococcus aureus* (MRSA) strains was provided by the Department of Biotechnology and Genetic Engineering at Islamic University, Kushtia, Bangladesh. The bacterial strain was isolated and identified from the drainage water of a medical center. Methicillin resistance was screened using cefoxitin disc diffusion (30 µg) according to Clinical and Laboratory Standards Institute (CLSI) M100 guidelines. Zone diameters ≤ 21 mm (CLSI) were interpreted as MRSA, while larger zones indicated methicillin-susceptible *S. aureus* (MSSA). The identified MRSA strain also showed resistance to β-lactam antibiotics, including methicillin, oxacillin, and ciprofloxacin, among others.

### 2.5. Analytical analysis

#### 2.5.1. Qualitative preliminary phytochemical screening.

A range of standard colorimetric techniques was utilized to detect different phytochemical groups present in the respective extracts, following previously established protocols [15]. For flavonoids, 25 mg of each extract was dissolved in 2.5 mL of methanol. The addition of 5% NaOH produced a yellow color, which became colorless upon adding a few drops of 10% HCl, indicating the presence of flavonoids. Tannins were detected using both ferric chloride and lead acetate assays. In the ferric chloride test, 2 mL of a 10 mg/mL extract solution was mixed with 5% FeCl₃, producing a greenish-black or reddish-black color. In the lead acetate test, the extract solution was combined with 10% Pb(C₂H₃O₂)₂ solution, forming a grayish-white or creamy precipitate. Terpenoids were identified by dissolving 10 mg of extract in 8 mL chloroform, followed by the gentle layering of concentrated H₂SO₄ along the tube; the formation of a brownish ring at the interface confirmed terpenoids. Steroids were detected by treating 10 mg of extract with concentrated H₂SO₄ and gently shaking, resulting in a reddish or dark lower layer. Saponins were assessed by mixing 0.5 mg/mL extract solution with 3 mL distilled water, shaking vigorously for 2–3 minutes, and allowing the mixture to stand for 10 minutes; persistent foam indicated the presence of saponins. Reducing sugars were tested by mixing 25 mg of extract with equal volumes of Fehling’s solutions A and B, then heating for 5–10 minutes; a yellowish or reddish-orange precipitate confirmed their presence. Alkaloids were detected by dissolving 50 mg of extract in 5 mL of 2% HCl, boiling briefly, and filtering; the addition of 1 mL Mayer’s reagent produced a creamy-white precipitate. Cardiac glycosides were identified using the Keller–Killiani method, where 25 mg of extract was treated with 4 mL glacial acetic acid, 1 mL 5% FeCl₃, and 1 mL 1 N HCl, producing a brown ring at the interface. All tests were performed in triplicate to ensure reproducibility.

#### 2.5.2. FT-IR spectroscopic analysis.

FT-IR was conducted on the respective extract based on a previously described method [7]. In short, the extract was pressed into a KBr disc (1 mg of extract blended with 100 mg of spectroscopic-grade KBr), which was subsequently loaded into the FT-IR instrument’s sample chamber. The IR spectrum was captured within the wavenumber range of 4500–400 cm ⁻ ¹, with a spectral resolution of 4 cm ⁻ ¹. Each spectrum was obtained after 32 scans under ambient temperature (25 ± 1 °C).

#### 2.5.3. GC-MS analysis.

The GC-MS examination was carried out following an established protocol [16]. Phytocompounds present in the respective extract were identified using a Shimadzu triple quadrupole GC-MS-TQ8040 system. Helium served as the carrier gas (99.999% purity), while an Rtx-5MS capillary column (30 m in length, 0.25 mm internal diameter, and 0.25 µm film thickness) functioned as the stationary phase. The column oven was programmed to start at 50°C for 1 minute, ramped up to 200°C for 2 minutes, and finally held at 300°C for 7 minutes. During the 40-minute analysis, the injector temperature was steadily maintained at 250°C. A 1 µL aliquot of the sample was introduced into the GC inlet at a constant flow rate of 1 mL/min using spitless injection mode. Detector parameters were set as follows: Q3 full scan in mass spectrometry, scanning from 50 to 600 m/z, with a scan speed setting of 2000, interface temperature at 250°C, ion source temperature at 230°C, scan duration of 0.3 seconds per scan, and an ionization energy of 70 eV. Phytochemical identification was based on retention time and spectral data comparisons, which were further validated by matching spectra to the NIST database for verification of compound identity.

### 2.6. *In vitro* studies

#### 2.6.1. Antibacterial activity assessment by agar-well diffusion assay.

The antibiotic activity of MEECF, EEECF, EAEECF, MEECL, EEECL, and EAEECL against MRSA was evaluated using agar-well diffusion assay, according to previously established protocols [16]. Briefly, the cryopreserved strain of MRSA was thawed and plated separately on LB agar medium. The plates were incubated at 37°C to allow bacterial colony development. One selected colony was then transferred to 25 mL of LB broth and cultured at 37°C under constant shaking at 250 rpm until its optical density (OD) at 600 nm wavelength reached 0.4, as measured with a Multiskan Sky Microplate Spectrophotometer (Multiskan Sky with Cuvette and Touch Screen, Cat #A51119700C, Thermo Fisher Scientific, USA). 50 µL of this culture was uniformly spread over LB agar plates, and four wells were punched with a sterile cork borer. The MEECF, EEECF, EAEECF, MEECL, EEECL, and EAEECL stock solutions (1000 µg/mL) were dissolved in their respective solvent and serially diluted to obtain concentrations of 500, 250, 125, and 62.5 µg/mL. Each concentration was introduced into separate wells on the LB agar plates. A standard antibacterial disc containing 10 µg of methicillin was placed centrally on each plate and used as the positive control. Plates were then incubated at 37°C for 16 ± 1 hours, and the inhibition zones were measured to determine antibacterial efficacy. Each test was performed in triplicate.

#### 2.6.2. Antibacterial activity assessment by MIC and MBC assay.

The MIC of MEECF, EEECF, EAEECF, MEECL, EEECL, and EAEECL was determined through a two-fold serial dilution method, following a previously described protocol with slight adjustments [7]. Briefly, the initial stock solutions of MEECF, EEECF, EAEECF, MEECL, EEECL, and EAEECL were prepared at a concentration of 2000 µg/mL and subsequently serially diluted in LB broth using 25 mL glass tubes to obtain final concentrations of 1000, 500, 250, and 125 µg/mL. These tubes were then inoculated with a bacterial suspension adjusted to the 0.5 McFarland standards (~1 × 10⁸ CFU/mL). One glass tube containing only LB broth (equivalent in volume to the extract-containing tubes) served as the negative control, and another tube containing LB broth with the bacterial suspension adjusted to the 0.5 McFarland standard (~1 × 10⁸ CFU/mL) served as the growth control. All tubes were incubated at 37°C for 24 h, and turbidity was monitored visually. The lowest concentration of each extract that inhibited visible bacterial growth was recorded as the MIC. For the determination of MBC, 50 µL from each bacterial culture was spread onto LB agar plates and incubated at 37°C for 16 h. The minimum concentration of the respective extracts that completely inhibited bacterial colony growth on the agar plate was recorded as the MBC. The MIC and MBC values reported in the Results section were determined strictly following the procedures described above, and all reported values are fully consistent with the experimental methodology. All experiments were conducted in triplicate to ensure reproducibility.

### 2.7. *In silico* study of antibacterial activity

#### 2.7.1. Protein structure retrieval and preparation.

Although 1VQQ offers higher resolution, it is an apo (ligand-free) structure and does not reflect ligand-induced conformations of the active and allosteric sites. 3ZG0, in contrast, is ligand-bound with ceftaroline and a peptidoglycan fragment at the allosteric site, providing a biologically relevant conformation. This makes 3ZG0 more appropriate for docking studies and for predicting phytocompound interactions with functionally relevant PBP2a sites. Therefore, the three-dimensional tertiary structures of the PBP2a (PDB ID: 3ZG0) and β-Lactamase (PDB ID: 3BLM) proteins in PDB format were downloaded via the RCSB protein data bank (RCSB PDB: Homepage). The crystallographic structures of β-Lactamase and PBP2a were resolved with a resolution of 2.00 Å and 2.60 Å, respectively. Initially, the structure of these two proteins was prepared using the Discovery Studio software. In this preparation method, the following criteria, including water molecules, different associated ions, and several cofactors, were removed from the crude protein. The non-polar hydrogen bonds were merging, and polar hydrogen bonds were added. Finally, the energy was minimized by the Swiss PDB viewer tools for preparing the protein molecules.

#### 2.7.2. Preparation of ligand.

A total of 383 instinct compounds were detected in the GC-MS output, which were screened using preliminary drug likeness approaches, including RO5 violation and gastrointestinal permeability checks, two crucial factors for selecting drug candidates. One hundred and one (101) compounds were obtained, which exhibited pharmacologically acceptable properties. These phytochemicals were obtained in 3D SDF format from the PubChem database (https://pubchem.ncbi.nlm.nih.gov/) and prepared using AutoDock 4. During ligand preparation, aromatic carbon atoms were first detected, followed by the generation of the torsion tree. The correct atom types were assigned, and non-polar hydrogens were merged. Finally, all the compounds were energy-minimized using the MMFF94 force field and saved in PDBQT format.

#### 2.7.3. Molecular docking studies.

The *in silico* docking analysis study is among the key strategies used to determine the active site pose of a protein with potential ligand molecules. The prepared ligands were docked with the prepared protein by utilizing PyRx software via the AutoDock Vina wizard. The exhaustiveness was set to 8, and the energy range was maintained at 4 kcal/mol during the docking procedure to ascertain optimal binding affinities between the ligands and the protein. For docking the two target proteins (β-Lactamase and PBP2a), a grid box was generated for the docked ligand in the proteins’ active pockets mentioned in S1 Table. For the β-Lactamase, the grid box center coordinates were defined as X = 59.4384 Å, Y = 42.9518 Å, Z = 48.4556 Å, where the center was chosen at the position of X = 59.4384 Å, Y = 42.9518 Å, Z = 48.4556 Å, respectively. Whereas the PBP2a, its grid box size was assigned as X = 18.1968 Å, Y = 19.1843 Å, Z = 51.9170 Å, and the center was set at X = 69.8303 Å, Y = 59.6052 Å, Z = 130.9461 Å, correspondingly. The interactions among the proteins (β-Lactamase and PBP2a) and their respective ligands were interpreted by estimating their docking scores in Kcal/mol. The different chemicals and ligand-binding residues were identified using the Maestro Schrodinger viewer from the protein-ligand docking complex pose.

#### 2.7.4. Molecular dynamics (MD) simulation.

MD simulations were carried out to assess the structural persistence of the protein-ligand complexes under simulated physiological conditions. To evaluate atomic-level motions and dynamics of the ligands within the protein-bound environment, 200 ns long MD simulations with a 2 fs integration time step were performed to examine the dynamic stability of the protein-ligand complexes. The physical motions of atoms in protein molecules were examined using Schrödinger’s Desmond module (Release 2020−3) operating in a Linux environment. A cubic simulation box with Simple Point Charge (SPC) water molecules was created for each complex, which had dimensions of 10 × 10 × 10 Å³, to maintain a constant system volume. Na^+^ and Cl^-^ ions were randomized into the simulation environment to achieve a physiological salt content level of approximately 0.15 M. The OPLS3e force field enabled the stabilization and relaxation of the system. During the simulation, temperature was maintained at 300 K and pressure at 1.01 bar within the NPT (constant pressure-constant temperature) ensemble, summarized in S1 Table. Through the computation of parameters such as RMSD, RMSF, Rg, SASA, and protein-ligand contacts, overall structural stability and dynamic properties of each complex were assessed.

#### 2.7.5. Pharmacokinetics (PK) and toxicity analysis.

The body’s interaction with an administered drug depends on its chemical and physical characteristics, including lipophilicity, water solubility, pharmacokinetics (such as GI absorption and BBB permeability), adherence to the Rule of Five for drug-likeness, synthetic accessibility, and toxicity prediction. These factors are crucial criteria in determining the effectiveness of a drug. Diverse computational tools were used to predict the initial potential of selected compounds with multi-targeting capabilities, showing higher molecular docking scores than the control drug methicillin. Thus, an online tool named SwissADME (SwissADME) was utilized to evaluate these parameters, excluding those related to toxicity and renal clearance. Admetlab2 (ADMETlab 2.0) and Admetsar2 (admetSAR) were employed for toxicity and excretion (renal clearance) analyses, respectively. All predictions were performed using default server parameters.

### 2.8. Statistical analysis

Antibacterial activity results are expressed as the mean followed by the standard deviation (SD) derived from three independent experiments. These experiments were conducted with varying concentrations of the respective extracts. The statistical analysis was performed using one-way ANOVA in OriginLab version 2018, followed by Bonferroni’s and Tukey’s post hoc tests.

## 3. Results

### 3.1. Analytical study

#### 3.1.1. PPS of *Eichhornia crassipes* flowers and leaves.

The preliminary phytochemical screening (PPS) of methanolic, ethanolic, and ethyl acetate extracts of *E. crassipes* flowers and leaves revealed the presence of diverse classes of bioactive compounds, including flavonoids, tannins, alkaloids, cardiac glycosides, saponins, terpenoids, steroids, and reducing sugars. Detailed qualitative test outcomes are summarized in Table 1, highlighting the intensity of color change and test confirmation results for each extract. As illustrated in S1 Fig, most extracts showed strong positive reactions for flavonoids, tannins, terpenoids, and alkaloids, which are known to contribute to antibacterial potential. Flavonoids were confirmed by yellow coloration in the alkaline reagent test, while tannins showed greenish-black coloration with FeCl₃. Similarly, saponins were indicated by stable foam formation, and alkaloids were identified by creamy precipitate formation in Mayer’s test. These findings indicate that both flowers and leaves of *E. crassipes* are rich in phytochemical constituents linked to antimicrobial, antioxidant, and anti-inflammatory properties.

**Table 1:** Preliminary phytochemical screening of MEECF, EEECF, EAEECF, MEECL, EEECL, and EAEECL showed the co-existence of different classes of phytochemicals.

Test Name	Color appearance	Extracts						Phytochemical class
		MEECF	EEECF	EAEECF	MEECL	EEECL	EAEECL	
Alkaline reagent test	Colorless	+	+	+	+	+	+	Flavonoids
FeCl_3_ test	Greenish-black/Reddish-black	+	+	+	+	+	+	Tannins
Pb(C₂H₃O₂)₂ test	Gray-white precipitate/Cream gelatinous precipitate/ Precipitate	+	+	+	+	+	+	Tannins
Salkowski’s test	Brown-layer at the upper interface/Bluish-brown or greenish-brown layer at the interface/Formation of yellow-colored lower layer layer	+	+	+	+	+	+	Terpenoids
Salkowski’s test	A blackish layer at the bottom/dark-red color on the bottom surface/greenish-black color appears in the lower layer	+	+	+	+	+	+	Steroids
Foaming test	Stable foam formation	+	+	+	+	+	+	Saponins
Fehling’s test	Yellowish color/reddish-orange precipitate	+	+	+	+	+	+	Reducing sugar
Mayer’s test	Creamy-colored precipitation/Creamy-white precipitation	+	+	+	+	+	+	Alkaloids
Keller-Kiliani test	Reddish or dark-brown color	+	+	+	+	+	+	Cardiac glycosides

“−” signifies the absence of specific phytochemicals. “+” indicates the presence of specific phytochemicals.

#### 3.1.2. FT-IR spectroscopic functional group analysis.

As shown in Table 2 and S2 and S3 Figs**, t**he FT-IR spectra confirmed the presence of multiple functional groups characteristic of biologically active compounds. The major absorption peaks corresponded to –OH, –NH, C–H, C = O, and C = C stretching vibrations, indicating the presence of alcohols, phenols, amides, alkanes, ketones, esters, and aromatic compounds. Broad absorption bands around 3700−2970 cm ⁻ ¹ represented O–H and N–H stretching, suggesting alcohols, phenols, and amines. Peaks between 1710−1650 cm ⁻ ¹ corresponded to C = O stretching in carbonyl compounds (esters, ketones, and amides). Aromatic C = C and N–O vibrations appeared around 1630−1460 cm ⁻ ¹, while bands at 1440−1260 cm ⁻ ¹ indicated aliphatic C–H bending and C–O stretching. The 1070−520 cm ⁻ ¹ region reflected C–O, C–N, and C–X stretching, confirming esters, amines, and halide groups. FT-IR results revealed that *E. crassipes* extracts contain multiple functional groups typically associated with antimicrobial phytochemicals such as phenolics, terpenoids, and fatty acids.

**Table 2:** FT-IR spectral evaluation of MEECF, EEECF, EAEECF, MEECL, EEECL, and EAEECL. The distinct peak positions observed in the spectrum typically indicate the vibrational modes of specific chemical bonds.

Wavenumber (cm ⁻ ¹)	Functional Group Assignment	MEECF	EEECF	EAEECF	MEECL	EEECL	EAEECL
~3700−2970	O-H stretching (alcohols/phenols) or N-H stretching (amines)	Br	Br	Br	Br	Br	Br
2924.21 - 2851.88	C-H stretching (alkanes, asymmetric/symmetric)	w	m	m	w	m	m
1710.93 - 1655.00	C = O stretching (ketones or esters)	sh	sh	m	sh	sh	m
1630.88 - 1615.45	C = C stretching (alkenes) or amide C = O stretching	s	s	m	s	s	m
1517.08 - 1464.03	Aromatic C = C stretching or N-O symmetric stretching (nitro groups)	w	w	w	w	w	w
1444.75 - 1374.34	C-H bending (alkanes)	w	w	w	w	w	w
1401.34 - 1267.29	C-O stretching (alcohols or esters)	w	w	w	w	w	w
1265.36 - 1166.98	C-O stretching (esters) or C-N stretching (amines)	w	w	w	w	w	w
1063.79 - 1075.36	C-O stretching (alcohols/ethers) or S = O stretching (sulfonamides)	w	w	w	w	w	w
~880−520	C-X stretching (alkyl halides)	Br	Br	Br	Br	Br	Br
520−400	Lattice vibrations or metal-ligand interactions	–	–	–	w	w	w

Here, Br, s, m, sh, and w stand for the shape (relative intensity) of the peak and represent broad, strong, medium, shoulder, and weak intensity, respectively.

#### 3.1.3. GC-MS analysis.

The GC-MS chromatograms for MEECF, EEECF, and EAEECF revealed 74, 65, and 65 peaks, respectively, while MEECL, EEECL, and EAEECL exhibited 72, 63, and 44 peaks. Each peak corresponds to a specific phytochemical, and the relative abundance of the compounds was determined by comparing each peak’s average area against the total area beneath the retention time (RT) curve (S4 and S5 Figs). The detailed identification of compounds’ chemical name, molecular formula, RT, peak area (%), PubChem CID, and chemical nature is provided in S2**-**S7 Tables. Among the detected phytochemicals, several bioactive constituents known for antibacterial potential were predominant. In MEECF, key compounds included 9-Octadecenamide, (Z)- (26.14%), cholest-5-en-3-ol, (3α)-, TMS derivative (17.44%), 9,12,15-Octadecatrienoic acid, methyl ester (3.18%), allocryptopine (2.81%), and phytol (1.85%). In EEECF, the major compounds were n-Hexadecanoic acid (17.17%), 9,12,15-Octadecatrienoic acid (12.82%), β-Sitosterol (7.20%), stigmasterol (5.20%), and 9-Octadecenamide, (Z)- (5.72%). The EAEECF extract primarily contained (Z)6,(Z)9-Pentadecadien-1-ol (11.80%), n-Hexadecanoic acid (8.04%), stigmasterol (7.32%), and γ-Sitosterol (4.54%). In MEECL, predominant compounds were 9-Octadecenamide, (Z)- (30.02%), stigmasterol (11.37%), and β-Sitosterol (2.29%). For EEECL, the major compounds were 9-Octadecenamide, (Z)- (25.21%), stigmasterol (16.71%), n-Hexadecanoic acid (6.54%), and neophytadiene (4.24%). Finally, EAEECL exhibited high contents of stigmasterol (29.85%), 9-Octadecenamide, (Z)- (23.95%), and eicosyl isopropyl ether (3.00%). Collectively, the predominance of fatty acids (n-Hexadecanoic acid, linoleic acid derivatives), sterols (stigmasterol, β-Sitosterol), and fatty acid amides (9-Octadecenamide, (Z)-) across all extracts suggests a consistent presence of compounds with well-documented antibacterial properties, correlating with their observed bioactivities. The differences observed in the chemical profiles of the extracts can be explained by the varying polarities of the solvents used for extraction. Methanol is a highly polar solvent capable of extracting a broad spectrum of polar and moderately polar phytochemicals, including compounds containing polar functional groups such as ester (–COO–), ketone/oxo (C = O), and hydroxyl (–OH) groups. Ethanol, slightly less polar than methanol, can also efficiently extract these compounds, though with slightly reduced efficiency. Ethyl acetate, being moderately polar, is more suitable for semi-polar to non-polar compounds and is less efficient at isolating highly oxygenated molecules. As a result, each solvent enriched a distinct subset of metabolites from *E. crassipes*, which is consistent with the GC–MS results obtained in this study.

### 3.2. *In vitro* study

#### 3.2.1. Antibacterial activity assessment in agar well diffusion assay.

**Fig 1:**
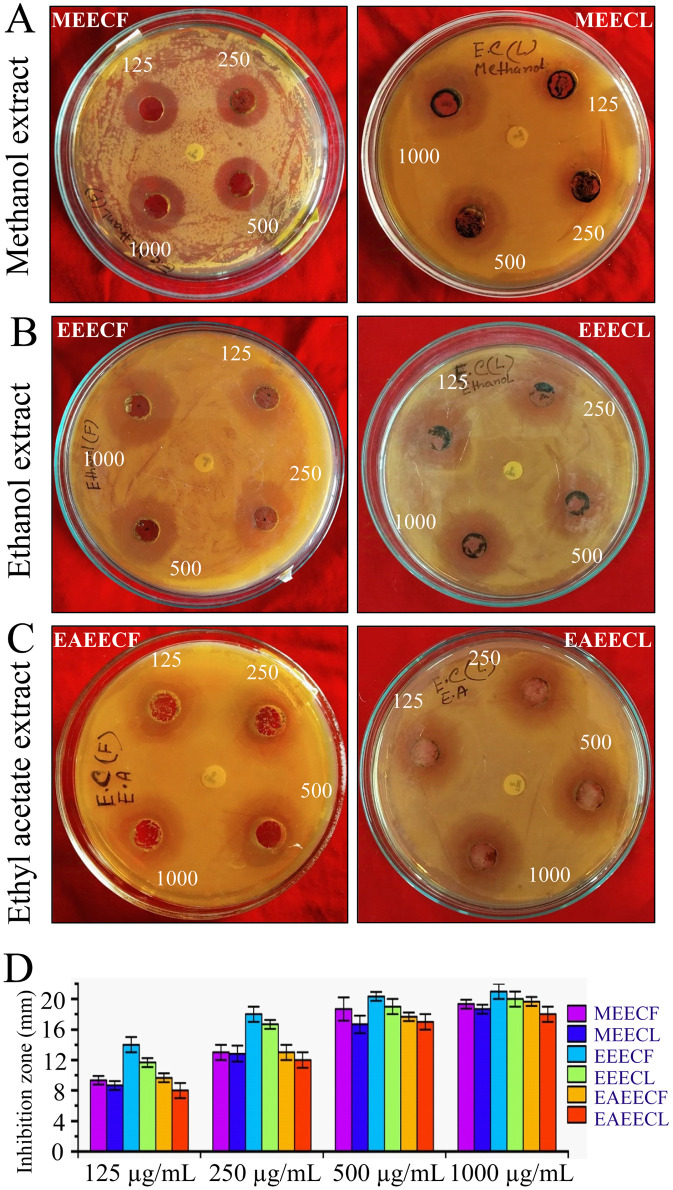
Antibacterial efficacy of different extracts derived from *Eichhornia crassipes* (Mart.) Solms flowers and leaves against methicillin-resistant *Staphylococcus aureus.* **(A)** Methanol extracts of flowers (MEECF) and leaves (MEECL). **(B)** Ethanol extracts of flowers (EEECF) and leaves (EEECL). **(C)** Ethyl acetate extracts of flowers (EAEECF) and leaves (EAEECL). **(D)** The zones of inhibition recorded through agar-well diffusion were measured in millimeters (mm), indicating the antibacterial impact of MEECF, EEECF, EAEECF, MEECL, EEECL, and EAEECL. As a positive control, the commercial antibiotic methicillin did not inhibit the growth of MRSA. The data were presented as mean ± standard deviation (STDEV, n = 3).

Given the persistent threat of MRSA, an evaluation was performed to determine the antibacterial efficacy of MEECF, MEECL, EEECF, EAEECF, EEECL, and EAEECL against MRSA. Our results demonstrated that the respective extracts prevented the growth of MRSA proliferation in a concentration-dependent fashion, producing inhibition zones ranging between 14 ± 1–21 ± 1 mm by MEECF, 8.666 ± 0.577 to 18.666 ± 0.577 mm by MEECL, 9.333 ± 0.577 to 19.333 ± 0.577 mm by EEECF, 11.66666667 ± 0.577350269 to 20 ± 1 mm by EEECL, 8 ± 1–18 ± 1 mm by EAEECF and 9.333 ± 0.577 to 19.333 ± 0.577 mm by EAEECF in the agar well diffusion method, using extract concentrations between 125–1000 µg/mL (Figs 1A-C).

Notably, the reference antibiotic methicillin, typically ineffective against MRSA due to resistance mechanisms, failed to exhibit antibacterial activity. The apparent antibacterial activity in the agar well diffusion assay highlighted comparative antibacterial activity of MEECF, MEECL, EEECF, EAEECF, EEECL, and EAEECL depicted in Fig 1D.

#### 3.2.2. Antibacterial activity assessment by MIC and MBC.

**Fig 2:**
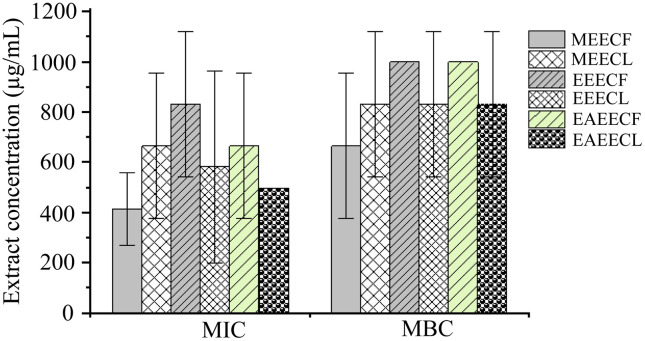
MIC and MBC of the six extracts of *Eichhornia crassipes* (Mart.) Solms flowers and leaves against MRSA in an antibacterial activity assay. The mean+STDEV (n = 3) was used to represent the data.


The minimum inhibitory concentration (MIC) refers to the lowest amount of an antimicrobial agent that completely inhibits visible microbial growth (indicated by the absence of turbidity) following overnight incubation. In contrast, the minimum bactericidal concentration (MBC) denotes the lowest concentration that achieves bacterial eradication after subculturing onto antibiotic-free solid agar media. As shown in S6-S11 Figs, our antibacterial experiment, some of the dilutions of the test extract exhibited no turbidity. In the MBC assay, the respective extracts at final concentrations corresponding to the MIC or higher resulted in the killing of 99.9% of the bacteria being tested The MIC of MEECF, MEECL, EEECF, EAEECF, EEECL, and EAEECL was determined to be 416.666 ± 144.337 µg/mL for MEECF, 666.666 ± 288.675 µg/mL for MEECL, 833.333 ± 288.675 µg/mL for EEECF, 583.333 ± 381.881 µg/mL for EEECL, 666.666 ± 288.675 µg/mL for EAEECF, and 500 ± 0 µg/mL for EAEECL Meanwhile, MBC values were observed as 666.666 ± 288.675 µg/mL for MEECF, 833.333 ± 288.675 µg/mL for MEECL, 1000 ± 0 µg/mL for EEECF, 833.333 ± 288.675 µg/mL for EEECL, 1000 ± 0 µg/mL for EAEECF, and 833.333 ± 288.675 µg/mL for EAEECL (Fig 2). It is worth noting that higher concentrations of the extracts were required during the MBC test to ensure complete bacterial killing, whereas lower concentrations were sufficient to halt bacterial growth in the MIC assay. The bactericidal potential of the extracts was further evaluated by calculating the ratio of MBC to MIC using the following formula:

$$\begin{array}{c} \frac{\text{MBC}}{\text{MIC}}\text{ratio}=\text{MBC value }(\mu \text{g}/\text{mL})/\text{MIC value }(\mu \text{g}/\text{mL}) \end{array}$$

The calculated MBC/MIC ratios were 1.6 for MEECF, 1.25 for MEECL, 1.2 for EEECF, 1.43 for EEECL, 1.5 for EAEECF, and 1.67 for EAEECL. As all ratios were below 4, these findings indicate that all tested extracts exhibited bactericidal activity against the examined bacterial strains. The MIC and MBC values presented here are fully consistent with the methodology described in the Materials and Methods section.

### 3.3. *In silico* study

#### 3.3.1. Multi-modal molecular docking interaction analysis.

Molecular docking is a vital approach for identifying the fundamental interaction mode of a ligand to a macromolecule with a 3D structure in computer-aided drug design and structural molecular biology [17]. Binding affinity studies were conducted between phytochemicals obtained from GC-MS and the target proteins β-lactamase and PBP2a for MRSA. Among 383 phytocompounds, 3 exhibited multi-modal binding affinities greater than methicillin with β-lactamase (Table 3), and 41 displayed multi-modal binding affinities greater than methicillin with PBP2a (Table 4**).** However, two phytocompounds, protopine (CID 4970) and propanoic acid, 2-methyl-,[(3aS,6R,6aR,9aS,9bR)-dodecahydro-6a-hydroxy-9a-methyl-3-methylene-2,9dioxoazuleno[4,5-b]furan-6-yl]-methyl ester (CID 615944), exhibited multi-modal and notably higher negative binding affinities for both target proteins (β-lactamase and PBP2a) compared to the control drug, methicillin (CID 6087). CID 4970 exhibited molecular binding within the active pocket, with a docking energy score of −8.1 kcal/mol for β-lactamase and −7.7 kcal/mol for PBP2a. In comparison, CID 615944 showed binding energy scores of −7.7 kcal/mol for β-lactamase and −7.6 kcal/mol for PBP2a. Since both phytocompounds demonstrated multi-modal binding and better binding poses for all targets compared to the control drug methicillin, they were selected as hit phytocompounds for further evaluation through a rational drug design process.

**Table 3:** The molecular docking interaction score between phytochemicals and the β-lactamase receptor (PDB ID: 3BLM) of methicillin-resistant *Staphylococcus aureus* was determined. Phytocompounds that exhibited a docking score of ≥ −7.6 are listed here, as this is the docking score of the control drug methicillin.

SL No.	Compound name	Compound CID	Canonical smiles	Docking Score (kcal/mol)	Plant extracts	GI	BBB
1	Protopine	4970	CN1CCC2=CC3 = C(C = C2C(=O)CC4 = C(C1)C5 = C(C = C4)OCO5)OCO3	−8.1	MEECF EEECF	High	Yes
2	Propanoic acid,2-methyl-,[(3aS,6R,6aR,9aS,9bR)-dodecahydro-6a-hydroxy-9a-methyl-3-methylene-2,9-dioxoazuleno[4,5-b]furan-6-yl]-methyl ester	615944	CC(C)C(=O)OCC1CCC2C(C3(C1(CCC3 = O)O)C)OC(=O)C2 = C	−7.7	MEECL	High	No
3	Allocryptopine	98570	CN1CCC2=CC3 = C(C = C2C(=O)CC4 = C(C1)C(=C(C = C4)OC)OC)OCO3	−7.6	MEECF EEECF	High	Yes
4	Methicillin	6087 (Control)	CC1(C(N2C(S1)C(C2 = O)NC(=O)C3 = C(C = CC = C3OC)OC)C(=O)O)C	−7.6	N/A	High	No

**Table 4:** The molecular docking interaction score between phytochemicals and the PBP 2a receptor (PDB ID: 3ZG0) of methicillin-resistant *Staphylococcus aureus* was determined. Phytocompounds that exhibited a docking score of ≥ −5.7 are listed here, as this is the docking score of the control drug methicillin.

SL No.	Compound Name	Compound CID	Canonical Smiles	Docking Score	Compound from which solvent	GI	BBB
1	2,5-Cyclohexadien-1-one, 4-[[4-(diethylamino)-2-methylphenyl]imino]-2-methyl-6-[[methyl(4-nitrophenyl)amino]methyl]	91741398 Simulation	CCN(CC)C1 = CC(=C(C = C1)N = C2C=C(C(=O)C(=C2)CN(C)C3 = CC = C(C = C3)[N+](=O)[O-])C)C	−7.8	MEECL	High	No
2	Sambucinol	5459101	CC1 = CC23C(CC1)(C4(CC(C(C4(O2)CO)O3)O)C)C	−7.7	MEECL	High	Yes
3	Protopine	4970	CN1CCC2=CC3 = C(C = C2C(=O)CC4 = C(C1)C5 = C(C = C4)OCO5)OCO3	−7.7	MEECF EEECF	High	Yes
4	Propanoic acid,2-methyl-,[(3aS,6R,6aR,9aS,9bR)-dodecahydro-6a-hydroxy-9a-methyl-3-methylene-2,9-dioxoazuleno[4,5-b]furan-6-yl]-methyl ester	615944	CC(C)C(=O)OCC1CCC2C(C3(C1(CCC3 = O)O)C)OC(=O)C2 = C	−7.6	MEECL	High	No
5	2,3:5,6-Di-O-1-Cyclohexylieden-1,4-cyclohexandiallylether	15704917	C = CCOC1C2C(C(C3C1OC4(O3)CCCCC4)OCC = C)OC5(O2)CCCCC5	−7.6	MEECF	High	Yes
6	3-[N’-(3H-Indol-3-ylmethylene)-hydrazino]-5-methyl-[1,2,4]triazol-4-ylamine	9600489	CC1 = NN = C(N1N)NN = CC2C=NC3 = CC = CC = C23	−7.5	MEECL	High	No
7	Ergost-25-ene-3,6-dione, 5,12-dihydroxy-, (5alpha,12beta)-	91692405	CC(CCC(C)C(=C)C)C1CCC2C1(C(CC3C2CC(=O)C4(C3(CCC(=O)C4)C)O)O)C	−7.5	EAEECL	High	No
8	Methyl-2,3:4,6-di-O-furylidenealphad-mannopyranoside	91697695	COC1C2C(C3C(O1)COC(O3)C4 = CC = CO4)OC(O2)C5 = CC = CO5	−7.3	MEECF	High	No
9	Ethyl acetate	6452096	CCOC(=O)CCC(C)C1CCC2C1(C(CC3C2C(CC4C3(CCC(C4)O)C)O)O)C	−7.3	MEECL EEECF	High	No
10	1-Hydroxy-2,2,6,6-tetramethyl-3-piperidinomethyl-4-piperidone	566506	CC1(CC(=O)C(C(N1O)(C)C)CN2CCCCC2)C	−7.3	EAEECF	High	Yes
11	Fenpropathrin	47326	CC1(C(C1(C)C)C(=O)OC(C#N)C2 = CC(=CC = C2)OC3 = CC = CC = C3)C	−7.2	MEECL	High	Yes
12	Allocryptopine	98570	CN1CCC2=CC3 = C(C = C2C(=O)CC4 = C(C1)C(=C(C = C4)OC)OC)OCO3	−6.9	MEECF EEECF	High	Yes
13	2-Hydroxymethyl-2,6,8,8-tetramethyltricyclo[5.2.2.0(1,6)]undecane	591157	CC1(CC23CCC1C2(CCCC3(C)CO)C)C	−6.9	MEECF	High	Yes
14	Cyclopentadecanone, 2-methyl-	543427	CC1CCCCCCCCCCCCCC1=O	−6.9	MEECF	High	Yes
15	2(1H)-Naphthalenone, octahydro-4a-methyl-7-(1-methylethyl)-. (4aalpha,7beta,8abeta)-	41133	CC(C)C1CCC2(CCC(=O)CC2C1)C	−6.9	MEECF	High	Yes
16	3,5-Di-tert-butylphenol	70825	CC(C)(C)C1 = CC(=CC(=C1)O)C(C)(C)C	−6.8	MEECF MEECL	High	Yes
17	3-(6,6-Dimethyl-5-oxohept-2-enyl)-cyclohexanone	5364977	CC(C)(C)C(=O)CC = CCC1CCCC(=O)C1	−6.8	EAEECF	High	Yes
18	3H-3,10a-Methano-1,2-benzodioxocin-3-ol, octahydro-7,7-dimethyl-, (3alpha,6abeta,10abeta)-	606210	CC1(CCCC23C1CCCC(C2)(OO3)O)C	−6.7	EAEECL	High	Yes
19	Isolongifolan-8-ol	535389	CC1(CCC(C2C13CCC(C3)C2(C)C)O)C	−6.7	MEECL	High	Yes
20	Costal	14262761	CC12CCCC(=C)C1CC(CC2)C(=C)C = O	−6.7	EEECF	High	Yes
21	6-Hydroxy-4,4,7a-trimethyl-5,6,7,7a-tetrahydrobenzofuran-2(4H)-one	14334	CC1(CC(CC2(C1 = CC(=O)O2)C)O)C	−6.6	MEECL EAEECL	High	Yes
22	Succinic acid, 2-methylpent-3-yl 2-methoxyphenyl ester	91712382	CCC(C(C)C)OC(=O)CCC(=O)OC1 = CC = CC = C1OC	−6.5	MEECL	High	Yes
23	1-Cyclododecanone, 2-ethylidene	5375859	CC = C1CCCCCCCCCCC1=O	−6.5	MEECL	High	Yes
24	1-(3-Hydroxypropyl)-5,5,8a-trimethyldecahydronaphthalen-2-ol	535298	CC1(CCCC2(C1CCC(C2CCCO)O)C)C	−6.5	MEECL	High	Yes
25	Isocalamenediol	91747826	CC(C)C1CCC(C2C1(CC(=C)CC2)O)(C)O	−6.4	MEECL	High	Yes
26	Fumaric acid, ethyl 2-propylphenyl ester	91712905	CCCC1 = CC = CC = C1OC(=O)C = CC(=O)OCC	−6.4	MEECL	High	Yes
27	Cyclohexane, 1,1’-(1-methylethylidene)bis[4-(ethenyloxy)-	11833232	CC(C)(C1CCC(CC1)OC = C)C2CCC(CC2)OC = C	−6.3	EAEECL	High	Yes
28	N,N-Diethyl-1-naphthylamine	66547	CCN(CC)C1 = CC = CC2 = CC = CC = C21	−6.2	EEECF	High	Yes
29	2,3-Dioxabicyclo[2.2.2]oct-7-en-5-one, 1-(3-oxo-1-butenyl)-6,6,7-trimethyl	5363634	CC1 = CC2C(=O)C(C1(OO2)C = CC(=O)C)(C)C	−6.2	MEECL	High	Yes
30	1,4-Piperazinediethanol, alpha,alpha’-bis(phenoxymethyl)-	3095130	C1CN(CCN1CC(COC2 = CC = CC = C2)O)CC(COC3 = CC = CC = C3)O	−6.2	EEECL	High	No
31	2-Cyclohexen-1-one, 3-(3-hydroxybutyl)-2,4,4-trimethyl-	520295	CC1 = C(C(CCC1 = O)(C)C)CCC(C)O	−6.1	MEECF	High	Yes
32	1-(beta-d-Arabinofuranosyl)-4-difluoromethyl-5-bromouracil	13989305	C1 = C(C(=NC(=O)N1C2C(C(C(O2)CO)O)O)OC(F)F)Br	−6.1	MEECL	High	No
33	1-Cyclohexene-1-butanal, alpha,2,6,6-tetramethyl-	579157	CC1=C(C(CCC1)(C)C)CCC(C)C = O	−6	EAEECL	High	Yes
34	3-Buten-2-ol, 2-methyl-4-(1,3,3-trimethyl-7-oxabicyclo[4.1.0]hept-2-yl)-	5363622	CC1(CCC2C(C1C=CC(C)(C)O)(O2)C)C	−6	MEECF EEECF	High	Yes
35	Acetic acid, 4-t-butyl-4-hydroxy-1,5-dimethyl-hex-2-ynyl ester	579166	CC(C)C(C#CC(C)OC(=O)C)(C(C)(C)C)O	−5.9	MEECF	High	Yes
36	1-Monolinolenoyl-rac-glycerol	5367328	CCC = CCC = CCC = CCCCCCCCC(=O)OCC(CO)O	−5.9	MEECL	High	Yes
37	Ethyl geranyl acetate	5363297	CCC(C = C(C)CCC = C(C)C)OC(=O)C	−5.9	EAEECL	High	Yes
38	Geranyl isovalerate	5362830	CC(C)CC(=O)OCC = C(C)CCC = C(C)C	−5.9	MEECF	High	Yes
39	Cyclohexene, 1,5,5-trimethyl-6-acetylmethyl-	579163	CC1 = CCCC(C1CC(=O)C)(C)C	−5.8	EAEECL	High	Yes
40	(1S,4R,5R)-1,3,3-Trimethyl-2-oxabicyclo[2.2.2]octan-5-ol	439906	CC1(C2CCC(O1)(CC2O)C)C	−5.8	EEECL	High	Yes
41	Methicillin	6087 (Control)	CC1(C(N2C(S1)C(C2 = O)NC(=O)C3 = C(C = CC = C3OC)OC)C(=O)O)C	−5.7	N/A	High	No

#### 3.3.2. Interpretations of 3D, and 2D structure of protein-ligands interaction.

**Fig 3:**
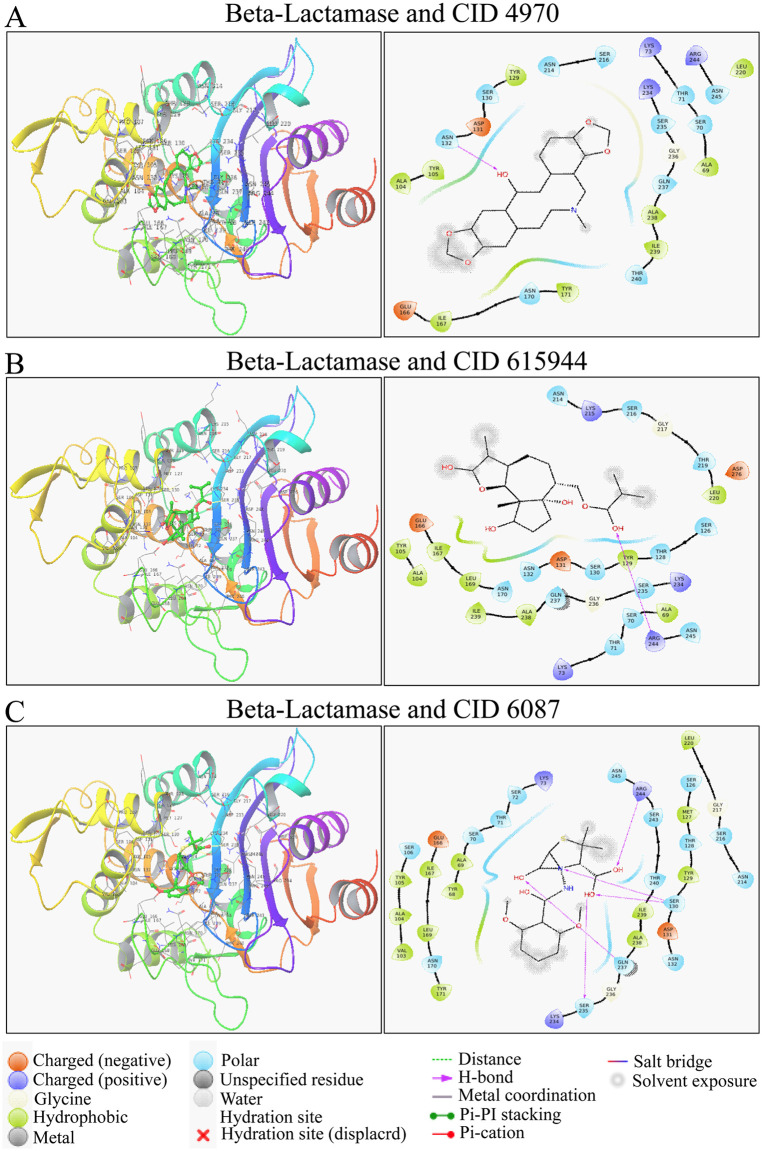
The molecular docking interactions of Beta-Lactamase with two selected phytocompounds and the control drug methicillin are illustrated in 3D (left) and 2D (right) representations. Panels (A-C) show the binding of CID 4970, CID 615944, and CID 6087 (control drug, methicillin), respectively, within the active pocket of the β-Lactamase protein.

**Fig 4:**
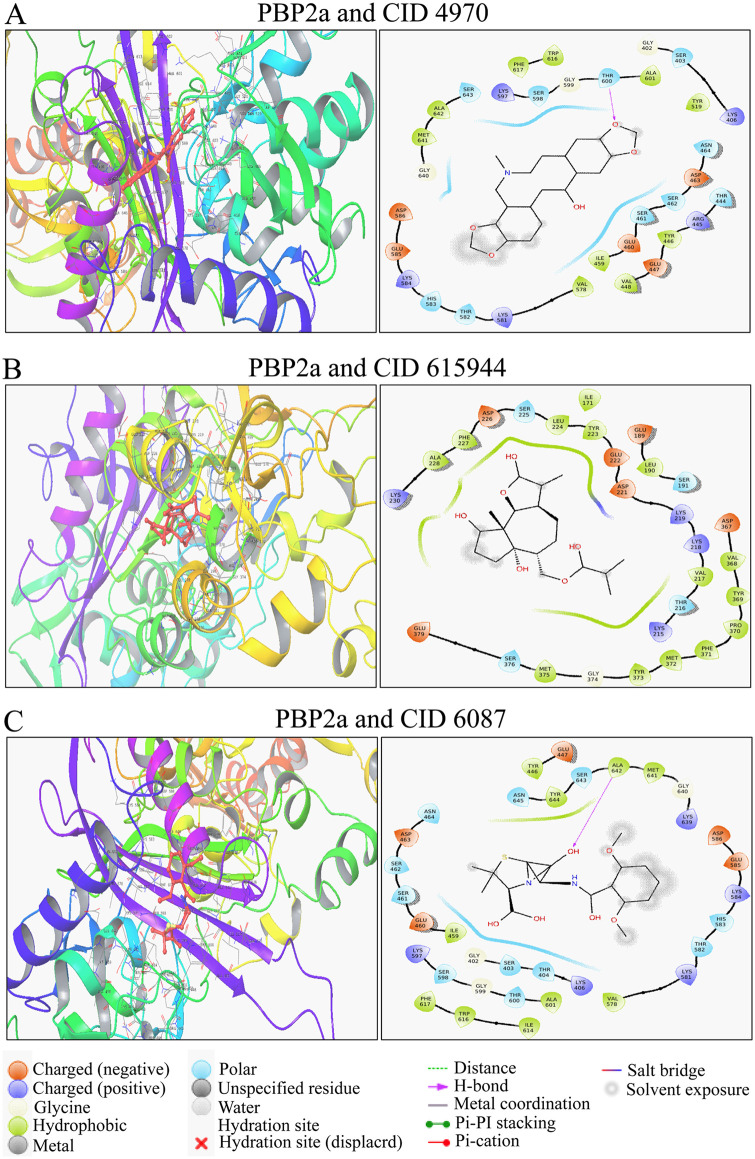
The molecular docking interactions of PBP2a with two selected compounds and the control drug methicillin are presented in 3D (left) and 2D (right) views. Panels (A-C) illustrate the binding of CID 4970, CID 615944, and CID 6087 (control drug, methicillin), respectively, bound within the active site of PBP2a.

The molecular docking interactions of CID 4970, CID 615944, and control drug methicillin (CID 6087) with the target proteins (β-actamase and PBP2a) have been illustrated by the Maestro module of the Schrödinger suite, depicted in Figs 3, 4. Different kinds of noncovalent bonds, like hydrophobic, polar, hydrogen bonds, and other bonds, contributed to the binding of ligands to the active pockets of target proteins to make an interaction between protein and ligands. Non-covalent bond formation in drug discovery plays a crucial role because ligands don’t alter the structure of the protein; rather, they bind with the target protein efficiently. The formation of hydrogen bonds is a crucial component of drug binding and appropriate metabolism. As indicated in the figures, CID 4970, when interacting with the β-lactamase receptor, formed one hydrogen bond, nine hydrophobic bonds, and several other bonds. Similarly, CID 615944 formed one hydrogen bond, seven hydrophobic bonds, and other bonds with the β-actamase protein. Methicillin (CID 6087) exhibited four hydrogen bonds, thirteen hydrophobic bonds, and additional bonds when binding to the β-lactamase receptor. Additionally, CID 4970 interacted with the PBP2a receptor, forming ten hydrophobic bonds, one hydrogen bond, and other bonds, while CID 615944, when binding to PBP2a, formed no hydrogen bonds, fourteen hydrophobic bonds, and additional bonds. Methicillin also formed ten hydrophobic bonds, one hydrogen bond, and other bonds with PBP2a.

#### 3.3.3. Protein-ligand binding interaction.

The molecular interactions between the selected ligands and the target proteins, β-lactamase and PBP2a, were explored using the Maestro interface of the Schrödinger suite, as represented in Figs 3, 4. The 2D and 3D visualizations revealed how the compounds are accommodated within the respective active sites through hydrogen bonding and hydrophobic interactions, contributing to their binding stability and specificity, as tabulated in S8 Table. For β-lactamase, both lead compounds (CID 4970 and CID 615944) and the control (CID 6087) exhibited strong affinity within the catalytic pocket. CID 4970 interacted with several key residues, including ALA 69, SER 70, THR 71, LYS 73, and GLU 166, establishing a single hydrogen bond with ASN 132. Hydrophobic contacts with ALA 69, TYR 105, TYR 129, ILE 167, TYR 171, and LEU 220 stabilized its binding conformation. Similarly, CID 615944 demonstrated extensive residue engagement involving ALA 69, SER 70, THR 71, TYR 105, and GLU 166, forming one hydrogen bond with ARG 244. Its hydrophobic network encompassed ALA 69, TYR 105, TYR 129, ILE 167, LEU 169, and ALA 238, indicating a strong anchoring within the hydrophobic cavity. The control compound, CID 6087, established multiple hydrogen bonds with SER 130, SER 235, GLN 237, and ARG 244, confirming its stable orientation in the active pocket. It also maintained hydrophobic interactions with residues TYR 68, ALA 69, TYR 105, MET 127, TYR 171, and ILE 239. Notably, all three ligands shared common hydrophobic contacts with ALA 69, TYR 105, TYR 129, and ILE 239, suggesting occupation of a conserved hydrophobic region crucial for β-lactamase inhibition. In the case of PBP2a, the binding patterns indicated a similar trend of strong noncovalent interactions. CID 4970 exhibited a stable conformation through one hydrogen bond with THR 600 and multiple hydrophobic contacts with TYR 446, VAL 448, ILE 459, ALA 601, and TRP 616. CID 615944, though lacking hydrogen bonding, maintained stability through hydrophobic interactions with residues such as ILE 171, LEU 190, TYR 223, PHE 227, TYR 369, and MET 372, implying its binding is primarily driven by van der Waals forces and π–π stacking. The control compound, CID 6087, formed a hydrogen bond with ALA 642 and numerous hydrophobic interactions involving TYR 446, ILE 459, TRP 616, PHE 617, and MET 641. Importantly, residues TYR 446, TRP 616, PHE 617, MET 641, and ALA 642 were common interaction points among the three ligands, signifying a conserved hydrophobic core at the PBP2a active site. The interaction profiles revealed that both lead compounds exhibited comparable or even stronger binding patterns than the control, primarily through stable hydrophobic interactions complemented by occasional hydrogen bonding. These conserved interactions suggest that CID 4970 and CID 615944 may effectively block the functional domains of β-lactamase and PBP2a, thereby interfering with bacterial resistance mechanisms.

#### 3.3.4. Examination of the protein-ligand complexes’ structural stability of the best-hit compound through molecular dynamics simulation.

Molecular dynamics (MD) simulation is a robust method to observe the conformational changes where ligands and proteins are both permitted to run within a defined period under simulated physiological conditions. A 200 ns simulation was conducted to examine the flexibility, conformational dynamics, and binding interactions of the complex, addressing metrics such as RMSD, RMSF, Rg, SASA, and protein-ligand interaction profiling.

**Fig 5:**
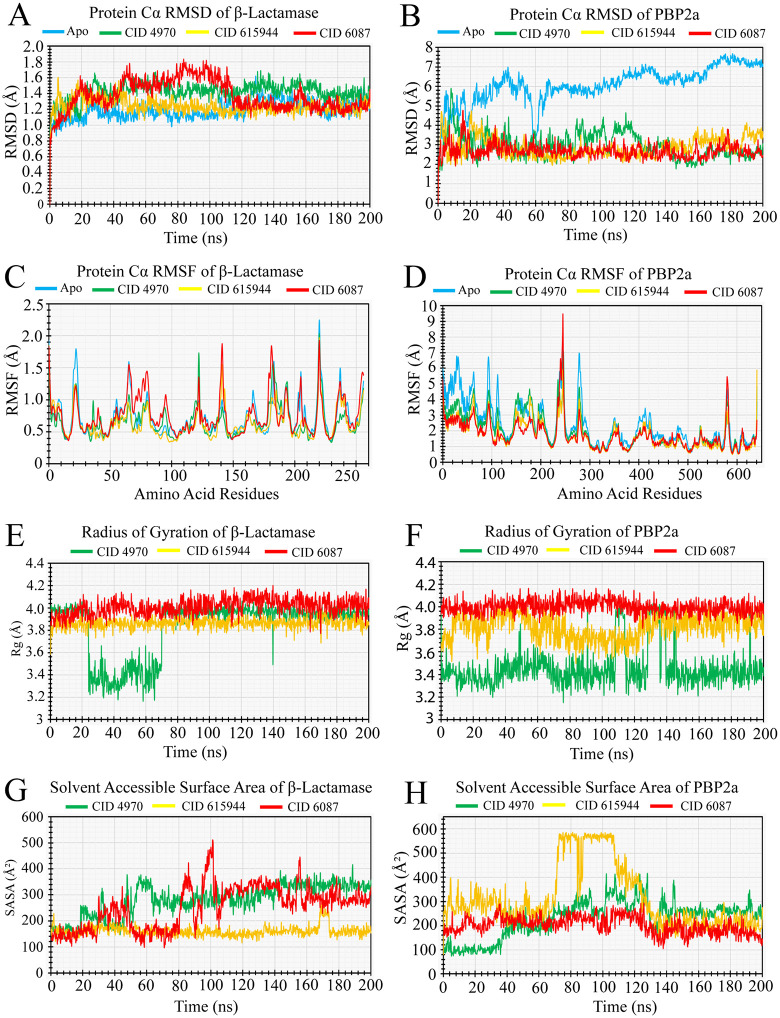
MD simulations of the selected phytocompounds and target protein complexes were conducted over a 200-nanosecond trajectory timeframe. (**A** and **B**) show the RMSD values from the Cα atoms of β-lactamase and PBP2a as apoproteins, as well as for the phytocompounds CID 4970, CID 615944, and the control drug methicillin (CID 6087) in the docked complexes. (**C** and **D**) present the RMSF values obtained from the Cα atoms within the protein-ligand docked complexes. The RMSF values for the β-lactamase and PBP2a as apoproteins are illustrated in blue, the control drug (methicillin) in violet, and the two selected phytocompounds, CID 4970 in red and CID 615944 in green, complexed with the β-lactamase and PBP2a proteins, respectively. **(E and F)** Illustrate the radius of gyration (Rg) of the protein-ligand complexes. The Rg values of the selected phytocompounds CID 4970 and CID 615944, along with the control drug (ampicillin), in complex with β-lactamase and PBP2a are delineated in red, green, and violet, respectively. **(G and H)** Provide a graphical presentation of the protein-ligand complex’s SASA values. The SASA values for the two phytocompounds (CID 4970 and CID 615944) and the control drug (methicillin) in complex with β-lactamase and PBP2a are represented by red, green, and violet, respectively.

**RMSD Analysis:** The RMSD analysis evaluates the overall conformational stability of proteins during the simulation by measuring the average atomic displacement over time. Deviations within 1–3 Å are generally considered stable, indicating that the protein-ligand complex maintains equilibrium throughout the simulation period. The RMSD values for protein Cα atoms were calculated across the 200-ns simulation trajectory. As illustrated in Figs 5A **and** 5B and Table 5, the RMSD profiles of the β-lactamase and PBP2a complexes demonstrated acceptable fluctuations during ligand binding compared to their respective apo forms and the reference drug methicillin (CID 6087). For β-lactamase, the calculated Cα RMSD values were 1.188 Å for the apoprotein, 1.422 Å for CID 4970, 1.240 Å for CID 615944, and 1.373 Å for the methicillin complex. These values remained well within the acceptable range, indicating stable conformations throughout the simulation period. Among the ligand-bound systems, the CID 615944-β-lactamase complex exhibited the lowest average deviation, suggesting strong structural rigidity and minimal conformational drift. The CID 4970-β-lactamase complex also maintained a stable trajectory, comparable to the control complex, confirming that both compounds effectively stabilized the protein backbone during dynamic motion. In the case of PBP2a, higher RMSD fluctuations were observed compared to β-lactamase, consistent with its larger structural domain and inherent flexibility. The average RMSD values were 6.070 Å for the apoprotein, 2.973 Å for CID 4970, 2.858 Å for CID 615944, and 2.686 Å for the methicillin complex. Despite slightly elevated deviations, both CID 4970 and CID 615944 complexes displayed consistent RMSD plateaus after the initial equilibration phase, reflecting structural convergence and stable protein-ligand binding over the 200-ns trajectory. Taken together, these results confirm that both CID 4970 and CID 615944 maintained stable interactions with β-lactamase and PBP2a throughout the 200-ns simulation. The RMSD trends of both protein-ligand complexes closely resembled those of the control drug and stabilized within the acceptable range, indicating that the selected ligands were able to preserve structural integrity and stable binding orientations within the active pockets of both target proteins.

**Table 5:** Values of simulation parameters of beta-lactamase, PBP2a in complex with CID 4970, CID 615944 and CID 6087 (Control) and apo form.

Receptor Name	Parameters	Value	Apo	CID 4970	CID 615944	CID 6087 (Control)
Beta Lactamase	Protein Cα RMSD	H. RMSD (Å)	1.62	1.685	1.606	1.836
		L. RMSD (Å)	0.827	0.958	0.826	0.758
		A. RMSD (Å)	1.188	1.422	1.240	1.373
	Protein Cα RMSF	H. RMSF (Å)	2.247	2.029	1.969	1.931
		L. RMSF (Å)	0.365	0.34	0.343	0.367
		A. RMSF (Å)	0.730	0.666	0.645	0.760
	Radius of gyration	H. Rg (Å)	N/A	4.076	3.998	4.201
		L. Rg (Å)	N/A	3.162	3.583	3.771
		A. Rg (Å)	N/A	3.830	3.864	4.009
	Solvent-accessible surface area	H. SASA (Å^2^)	N/A	414.899	269.314	511.286
		L. SASA (Å^2^)	N/A	129.668	115.002	96.883
		A. SASA (Å^2^)	N/A	280.534	160.742	252.802
PBP2a	Protein Cα RMSD	H. RMSD (Å)	7.666	5.887	1.527	4.773
		L. RMSD (Å)	1.970	1.69	4.731	1.735
		A. RMSD (Å)	6.070	2.973	2.858	2.686
	Protein Cα RMSF	H. RMSF (Å)	7.358	7.091	5.904	9.458
		L. RMSF (Å)	0.583	0.567	0.48	0.552
		A. RMSF (Å)	2.393	2.004	1.700	1.751
	Radius of gyration	H. Rg (Å)	N/A	4.054	4.029	4.166
		L. Rg (Å)	N/A	3.15	3.498	3.811
		A. Rg (Å)	N/A	3.460	3.821	4.002
	Solvent-accessible surface Area	H. SASA (Å^2^)	N/A	417.692	586.92	292.749
		L. SASA (Å^2^)	N/A	74.669	79.673	105.794
		A. SASA (Å^2^)	N/A	230.223	313.182	201.831

**RMSF Analysis:** RMSF profiling provides insight into the flexibility and localized movements of individual amino acid residues throughout the protein chain during simulation. This analysis helps identify regions of high mobility that may contribute to ligand accommodation or conformational shifts. As illustrated in Figs 5C **and** 5D and Table 5, RMSF values were determined for β-lactamase and PBP2a proteins complexed with CID 4970 and CID 615944, compared to their apoproteins and the control drug methicillin (CID 6087). The β-lactamase receptor exhibited average RMSF values of 0.730 Å, 0.666 Å, 0.645 Å, and 0.760 Å for the apoprotein, CID 4970, CID 615944, and methicillin complex, respectively. These results indicate minimal fluctuations within the β-lactamase binding site, suggesting that both ligands contributed to the maintenance of structural stability during the 200-ns simulation. For the PBP2a receptor, the average RMSF values were recorded as 2.393 Å for the apoprotein, 2.004 Å for the CID 4970 complex, 1.700 Å for the CID 615944 complex, and 1.751 Å for the control drug. The slightly reduced fluctuation observed in both CID 4970 and CID 615944 complexes indicates that ligand binding improved the rigidity of the protein structure, stabilizing the flexible loops within the active site. The highest atomic fluctuations were noted around specific residues, particularly ASP 120, TYR 272, SER 306, and GLN 607 for the PBP2a complex, while SER 53, LYS 99, GLY 156, LYS 175, and GLY 254 showed elevated peaks in β-lactamase. Overall, all ligand-bound systems exhibited well-controlled fluctuations within the acceptable range, reflecting stable and compact protein-ligand complexes over the 200-ns simulation period.

**Radius of Gyration Analysis:** The spatial distribution of atomic coordinates with respect to the protein’s center of mass, defined as the radius of gyration (Rg), provides critical insight into the overall compactness and stability of protein-ligand complexes. This parameter reflects whether ligand binding influences the structural folding or expansion of the protein macromolecule. The Rg values of β-lactamase and PBP2a complexes were analyzed over the 200-ns simulation period to assess the dynamic stability of CID 4970, CID 615944, and the control drug (methicillin, CID 6087), as illustrated in Figs 5E **and** 5F and Table 5. For β-lactamase, the average Rg values for the CID 4970- and CID 615944-bound complexes were 3.830 Å and 3.864 Å, respectively, while the control methicillin complex showed an average Rg of 4.009 Å. The observed Rg for the CID 4970 complex was slightly lower than both the CID 615944 and control complexes, suggesting that CID 4970 induced tighter packing and greater compactness of the protein structure. This implies that ligand binding contributed to an overall stable conformation during the simulation. Similarly, for PBP2a, the mean Rg values were 3.460 Å for CID 4970, 3.821 Å for CID 615944, and 4.002 Å for the control drug. The lower Rg values of the CID 4970 and CID 615944 complexes relative to the control indicate that the binding of both phytocompounds enhanced structural compactness and reduced molecular flexibility. Among the two, CID 4970 again demonstrated the most compact structure, suggesting superior stabilizing potential and stronger intramolecular packing. Collectively, these findings suggest that both phytocompounds promoted a more compact and stable conformation of β-lactamase and PBP2a compared to the reference drug methicillin. The consistently lower Rg values of CID 4970 across both target proteins further indicate its stronger structural stabilization effect during the 200-ns simulation period.

**SASA Analysis:** SASA serves as a vital structural parameter that reflects the degree of surface exposure of amino acid residues and provides insight into the solvation behavior, hydrophobicity, and functional dynamics of a protein during ligand interaction. Residues positioned on the outer protein surface often participate in molecular recognition and binding events, thereby influencing the conformational adaptability of macromolecules. As represented in Figs 5G **and** 5H and Table 5, the SASA profiles for β-lactamase and PBP2a were monitored throughout the 200-ns simulation for the apoprotein, the control drug (methicillin, CID 6087), and the lead compounds (CID 4970 and CID 615944). For β-lactamase, the SASA values fluctuated between 129.668 Å² and 414.899 Å² for the CID 4970 complex, and from 115.002 Å² to 269.314 Å² for the CID 615944 complex. In contrast, the methicillin-bound structure exhibited a range of 96.883 Å² to 511.286 Å². The average SASA values were recorded as 280.534 Å² for CID 4970, 160.742 Å² for CID 615944, and 252.802 Å² for the control complex. These findings suggest that the β-lactamase–CID 4970 complex exhibited higher solvent exposure relative to CID 615944 but remained well within the stable solvation range, indicating compact folding and reduced flexibility. Similarly, in the PBP2a complexes, the SASA values ranged between 74.669 Å² and 417.692 Å² for CID 4970, and from 79.673 Å² to 586.920 Å² for CID 615944. The methicillin complex displayed values between 105.794 Å² and 292.749 Å². The average SASA values were 230.223 Å² for CID 4970, 313.182 Å² for CID 615944, and 201.831 Å² for the control drug. The slightly elevated SASA observed for CID 615944–PBP2a indicates enhanced solvent exposure compared to the CID 4970 complex, which demonstrated better compactness and stability during the 200-ns trajectory. The SASA analysis revealed that both phytocompounds maintained favorable solvent accessibility compared to methicillin. Among them, CID 4970 displayed reduced surface area exposure, reflecting stronger packing interactions and a more stable protein–ligand interface for both β-lactamase and PBP2a during the entire simulation period.

**Fig 6:**
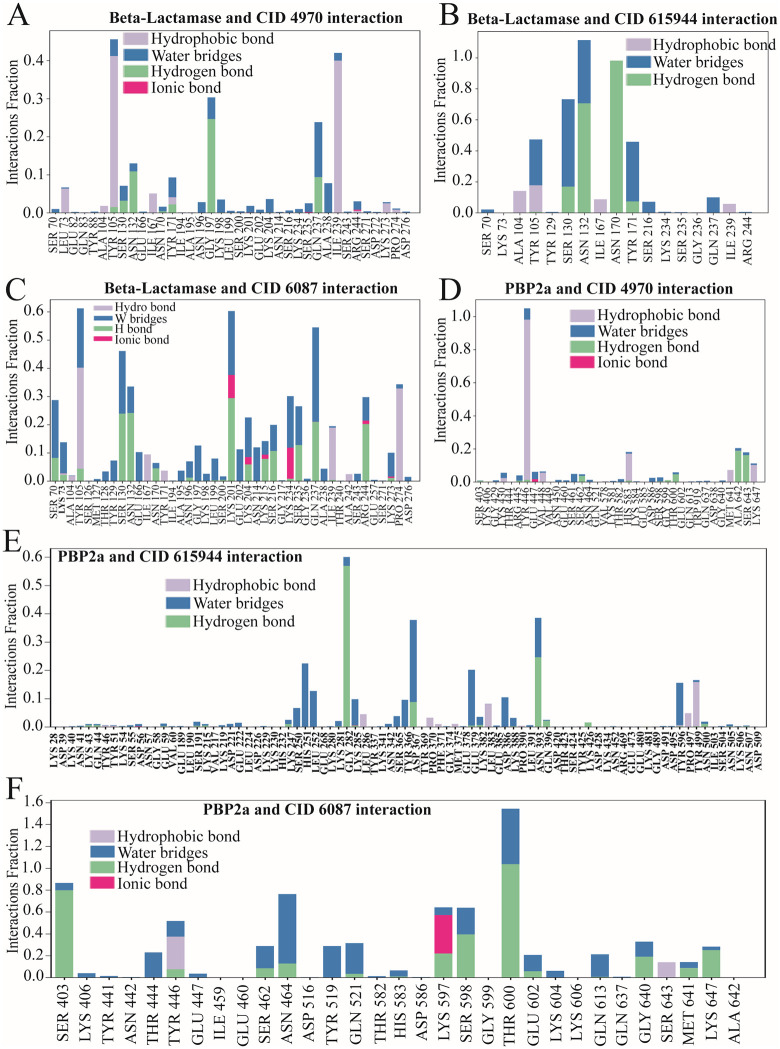
Bonding in the molecular dynamic simulation of CID 4970, CID 615944, and the control drug methicillin (CID 6087) with the target proteins (β-lactamase and PBP2a) was calculated from a 200-ns simulation. (**A**) β-Lactamase and CID 4970 interaction. (**B**) β-Lactamase and CID 615944 interaction. (**C**) β-Lactamase and CID 6087 association. **(D)** PBP2a and CID 4970 interaction. **(E)** PBP2a and CID 615944 interaction. **(F)** PBP2a and CID 6087 interconnection.

**Protein-ligand contact analysis:** Using the 100 ns simulation timeframe, the configurations of the target proteins complexed with the selected compounds and their interaction patterns were observed. Protein-ligand contacts within the receptors (β-lactamase and PBP2a) and the phytocompounds (CID 4970 and CID 615944) and the control drug were examined and illustrated in Fig 6, considering factors such as ionic bonding, water bridges, hydrogen bonding, and noncovalent interactions (hydrophobic bonding). Both compounds interacted during the 100 ns simulation, including ionic bonds, water bridges, hydrogen bonds, and hydrophobic interactions. These interactions facilitated stable binding between the compounds and the target proteins, which persisted throughout the simulation. CID 4970 exhibited water bridges, hydrophobic interactions, and hydrogen bonding with both PBP2a and β-lactamase. Similarly, CID 615944 interacted with β-lactamase and PBP2a through ionic bonds, water bridges, hydrophobic interactions, and hydrogen bonds. In contrast, the control ligand methicillin interacted with both receptors (β-lactamase and PBP2a) through consistent hydrogen bonds, hydrophobic contacts, solvent-mediated bridges, and electrostatic interactions.

#### 3.3.5. Pharmacokinetics, drug-likeness, and toxicity analysis.

The pharmacokinetics (PK), drug-likeness, and toxicity assessment are critical in drug development to ensure both safety and effectiveness for regulatory clearance. Accordingly, the PK, drug-likeness, and toxicity profiles of the two hit phytocompounds were evaluated. The PK properties of drug candidates are significantly influenced by their physicochemical parameters, including MW, HBD, HBA, RB, and TPSA. As shown in Table 6, both phytocompounds displayed molecular weights (≤500 g/mol), HBD (≤5), HBA (≤10), and TPSA values (≤140 Å²) within the optimal range, suggesting their potential for efficient oral absorption. The ideal range for RB is 0–11, and our phytocompounds fall within this, with RB values ranging from 0 to 4, suggesting favorable absorption characteristics. A cLogP value between 1 and 5 typically reflects a high likelihood of absorption, and both phytocompounds satisfy this requirement, with cLogP values ranging from 2.02 to 2.67. LogS, a measure of solubility, is ideally low, with a suitable range of −4.0 to 0.5. Both phytocompounds exhibited LogS values from −2.52 to −4.0, fitting well within the desired range. HIA predictions were also performed since they greatly impact systemic availability of a drug. CID 4970, CID 615944, and methicillin (CID 6087) exhibited high GI absorption. Renal excretion serves as a major pathway for eliminating both metabolized and unmetabolized compounds. Potential drug candidates are expected to demonstrate efficient and favorable excretion characteristics during drug development. We observed that protopine demonstrated high clearance, whereas CID 615944 and methicillin exhibited moderate clearance. Lipinski’s Rule of Five (RO5) provides a framework for evaluating the drug-like properties of compounds. Using the SwissADME server, we assessed the RO5 compliance of both hit phytocompounds and confirmed that they satisfied the criteria, indicating their potential as drug-like molecules. Evaluating the synthetic accessibility of potential drug candidates is a fundamental aspect of computational and medicinal chemistry. Both phytocompounds exhibited favorable synthetic accessibility scores, ranging from 3.48 to 4.65. An in-silico toxicity profiling was performed using the AdmetSAR2 web server, revealing non-hepatotoxic, non-carcinogenic, and non-mutagenic characteristics for both compounds. Based on these combined properties, the two hit phytocompounds were selected as the most promising candidates.

**Table 6:** The physicochemical characteristics, lipophilicity, water solubility, toxicity profiles, drug-likeness, and synthetic accessibility properties of the phytoconstituents.

Parameters		CID 4970	CID 615944	CID 6087
Physicochemical properties	MW (g/mol)	353.37	350.41	380.42
	Number of H-bond donors	0	1	2
	Number of H-bond acceptors	6	6	6
	TPSA	57.23 Å²	89.90 Å²	130.47 Å²
	Number of rotatable bonds (RB)	0	4	6
**Lipophilicity**	Consensus Log *P*_o/w_ (cLog*P*)	2.67	2.02	1.06
**Water Solubility**	Log *S* (ESOL)	−4.0	−2.52	−2.74
**Pharmacokinetics**	GI absorption	High	High	High
	Excretion: Renal clearance (mL/min/kg)	16.684 (High)	9.451 (Moderate)	6.118 (Moderate)
**Druglikeness**	Lipinski Rule of Five (RO5)	Yes; 0 violation	Yes; 0 violation	Yes; 0 violation
**Medicinal Chemistry**	Synthetic accessibility score	3.48	4.65	4.08
**Toxicity**	Hepatotoxicity	NT	NT	NT
	Carcinogenicity	NC	NC	NC
	Mutagenicity	NM	NM	NM

MW = Molecular weight ≤ 500, Number of rotatable bonds optimal = 0–11; Number of H-bond donors (acceptable range ≤5); Number of H-bond acceptors (acceptable range ≤10); Topological Polar Surface Area (TPSA) optimal range = 0–140; Consensus LogP (cLogP) = High lipophilicity (expressed as LogP, acceptable range: 1> and < 5); LogS = Log of the aqueous solubility optimal: −4 to 0.5 log mol/L; GI absorption = Gastrointestinal absorption; Renal clearance: > 15 mL/min/kg = High; Moderate: 5–15 mL/min/kg; Low < 5 mL/min/kg; RO5 = Lipinski’s rule of five; Synthetic accessibility from 1 (very easy) to 10 (very difficult); NT = Non-toxic; NC = Non-carcinogenic; NM = Non-mutagenic.

## 4. Discussion

Among several groups of *S. aureus*, the MRSA is the most successful strain to cause multidrug resistance. Managing *S. aureus* infections is becoming more challenging due to the rising antibiotic resistance, and currently, no effective drug has been developed [2,18]. MRSA exhibits broad resistance to beta-lactam antibiotics, including cephalosporins and carbapenems [19]. Considering this biomedical challenge, there is an increasing focus on investigating phytochemicals as promising antibacterial candidates, given that medicinal plants have proven to be a valuable source of antibacterial activity [20,21]. Although previous studies have demonstrated the antimicrobial activity of plant extracts against a range of pathogens, their efficacy against multidrug-resistant strains, especially MRSA, is still not well investigated.

Consequently, this study focused on evaluating the bactericidal effects of methanol, ethanol, and ethyl acetate extracts derived from the flowers and leaves of *E. crassipes* (Mart.) Solms, as well as their associated phytochemicals, against MRSA. Since β-lactamase and PBP2a play a pivotal role in antibiotic resistance in MRSA, we evaluated the *in vitro* antibacterial activity of MEECF, EEECF, EAEECF, MEECL, EEECL, and EAEECL against MRSA. In this study, we used methicillin as a reference antibiotic to confirm the resistance phenotype of the MRSA not as a therapeutic comparator. Since MRSA is defined by its resistance to methicillin, the use of methicillin in antimicrobial susceptibility testing provides direct evidence that the tested strain exhibits the expected resistant phenotype. This step helps verify the identity and resistance profile of the MRSA used in the experiment. Consistently, several antimicrobial studies involving MRSA have historically used methicillin or related β-lactam antibiotics as reference agents to demonstrate the resistance characteristics of the strain [22,23]. In this context, the inclusion of methicillin serves as an internal validation of the experimental model rather than as a clinical treatment comparator.

We assessed the *in silico* antibacterial effects of the phytocompounds in MEECF, EEECF, EAEECF, MEECL, EEECL, and EAEECL, with a specific emphasis on targeting β-lactamase and PBP2a. Since we observed concentration-dependent *in vitro* antibacterial activity of all extracts against MRSA, we metabolically annotated the extracts using GC-MS and identified 383 phytochemicals. These phytocompounds were subjected to molecular docking analysis, which identified CID 4970 in both MEECF and EEECF, and CID 615944 in MEECL, as the key phytocompounds. Protopine was detected in both MEECF and EEECF because it is a moderately polar benzylisoquinoline alkaloid. Its functional groups, such as methoxy and carbonyl groups, allow it to interact well with polar protic solvents like methanol and ethanol. Although methanol is more polar than ethanol, both solvents fall within the ideal polarity range for extracting medium-polar alkaloids like protopine. Therefore, its presence in both extracts is consistent with the chemical nature of the compound and the extraction properties of these solvents. CID 615944 is an oxygenated sesquiterpenoid ester and a semi-polar/moderately polar compound due to its hydrocarbon skeleton. However, its multiple polar oxygen-containing groups make it efficiently extracted by methanol rather than by ethyl acetate. This is because ethyl acetate, being moderately polar, favors semi-polar to non-polar compounds such as non-oxygenated terpenoids or fatty acid derivatives. This polarity-driven extraction explains why methanol and ethanol extracts were enriched in bioactive phytochemicals, which were subsequently evaluated in the *in silico* study for their potential to inhibit MRSA resistance determinants, β-lactamase, and PBP2a. Both compounds exhibited higher negative binding energy toward the target proteins (β-lactamase and PBP2a) compared to the control drug, methicillin, in our molecular docking study.

Supporting our findings, several previous studies have investigated the *in silico* antibacterial potential against multidrug-resistant (MDR) *Pseudomonas aeruginosa* by performing molecular docking analyses. These studies examined 82 phytochemicals from *Cassia occidentalis* L. and 51 phytochemicals from *Christella dentata* (Forssk.) Brownsey & Jermy, targeting LasR and LpxC in MDR *Pseudomonas aeruginosa*. They found that CID 102861, CID 136654, and CID 7150 in *Cassia occidentalis* L., and CID 536446 and CID 7734 in *Christella dentata* (Forssk.) Brownsey & Jermy exhibited higher negative binding affinities towards all target proteins (LasR and LpxC) compared to the co-crystallized ligands C12-HSL for LasR, BB-78485 for LpxC, and the control drug ampicillin [7,15].

In our subsequent identification of the best-hit phytochemicals from the molecular docking analysis, we found that CID 4970 and CID 615944 exhibited favorable physicochemical characteristics, including lipophilicity, water solubility, ADME properties, drug-likeness, medicinal chemistry, and acceptable toxicity profiles. As a result, these compounds were confirmed as the best-hit candidates for potential antibacterial agents and were selected for further molecular dynamics (MD) simulations, positioning them as promising lead compounds for treating MRSA. Similarly, previous studies identified methyl dihydrojasmonate (CID 102861), methyl benzoate (CID 7150), and 4a-methyl-4,4a,5,6,7,8-hexahydro-2(3H)-quinazolinone (CID 136654) from *Cassia occidentalis* L., as well as bicyclo[4.3.0]nonane, 2,2,6,7-tetramethyl-7-hydroxy- (CID 536446), and 1,4-diethylbenzene (CID 7734) from *Christella dentata* (Forssk.) Brownsey & Jermy, as the best-hit phytocompounds targeting LasR and LpxC in multidrug-resistant *Pseudomonas aeruginosa* [7,15], which were subsequently subjected to MD simulations to identify the lead phytocompound.

In all trajectories of the MD simulation, CID 4970 and CID 615944 exhibited stable binding in the active pockets of β-Lactamase and PBP2a over a 200-ns simulation period, indicating their potential as multi-targeting antibacterial lead phytochemical candidates against MRSA. Following this, research has not only identified lead phytochemicals (CID 102861, CID 7150, CID 136654, CID 536446, and CID 7734) against LasR and LpxC in *P. aeruginosa* [7,15].

Our *in silico*-identified anti-MRSA lead phytochemical, CID 4970, falls within the category of alkaloids [24]. In line with our findings, it has been reported that protopine, sourced from the aerial parts of *Hypecoum erectum* L., exhibits *in vitro* antibacterial activity against both Gram-positive (*Staphylococcus aureus* and *Bacillus cereus*) and Gram-negative (*Escherichia coli* and *Pseudomonas aeruginosa*) bacteria [25]. Another *in silico*-identified anti-MRSA lead phytochemical, CID 615944, belongs to the ester category. Consistent with our results, it has also been reported that hexadecanoic acid methyl ester, another ester compound, is the main active ingredient in clove (*Syzygium aromaticum*) alcoholic extract, exhibiting antibacterial activity against *S. aureus* W35, *P. aeruginosa* D31, *K. pneumoniae* DF30, and *K. pneumoniae* B45 [26].

We evaluated the antibacterial potential of the extracts through *in vitro* assays to support the *in silico* results. Similarly, it has been reported that *Papaver rhoeas L.* (red poppy), native to Turkey, is commonly used in folk remedies as a cough suppressant for children, a tea for sleep disorders, pain management, and as a natural sedative. The main component of this plant is protopine, and the acetone extract has demonstrated *in vitro* antibacterial efficacy against *Staphylococcus aureus [*27*]*. In line with this, hexadecanoic acid methyl ester is the predominant compound in the methanol extract of *Imperata cylindrica*, which showed inhibitory potential against the Gram-positive bacterium *Bacillus subtilis* and Gram-negative bacteria *Pseudomonas aeruginosa* and *Klebsiella pneumoniae [*28*]*.

Considering all, CID 4970 and CID 615944 have demonstrated the greatest potential as lead phytochemicals in the development of antibacterial therapy targeting MRSA. A limitation of the present study is the absence of a standard reference/control strain, such as methicillin-sensitive *Staphylococcus aureus* (MSSA) or a well-characterized ATCC strain, in the antimicrobial assays. Although the antibacterial activity was evaluated against a phenotypically characterized MRSA isolate, the lack of a standardized reference strain limits the direct comparability of the findings with previously published studies. In addition, this may affect the reproducibility and broader generalizability of the results across different experimental settings. Therefore, future studies should incorporate appropriate reference strains alongside clinical isolates to strengthen validation and ensure consistency of the observed antibacterial effects. The observed multi-target inhibitory potential, along with the predicted toxicity profiles and identification of potential lead compounds, is primarily based on *in silico* analyses. While such computational approaches are well established and widely applied in early-stage drug discovery for screening and prioritizing candidate molecules, the predicted multi-target inhibitory activity and potential lead compounds require further validation through comprehensive experimental investigations, including *in vitro* and *in vivo* studies, to confirm their biological efficacy, safety, and therapeutic relevance. Such validation is essential to establish the translational applicability of the present findings.

## 5. Conclusion

In this investigation, the flowers and leaves of *Eichhornia crassipes* (Mart.) Solms showed significant antibacterial effects against MRSA, as evidenced by the inhibitory zones in the agar well diffusion assay. The present study contributes to the identification of two predictive anti-MRSA phytochemical leads, CID 4970 and CID 615944, through *in vitro* and *in silico* analyses. These phytocompounds inhibited β-lactamase and PBP2a in MRSA, where β-lactamase is a key protein responsible for the degradation of β-lactam antibiotics, and PBP2a prevents the binding of β-lactam antibiotics to the bacterial cell wall. Together, these findings highlight the potential of *Eichhornia crassipes*–derived compounds as natural inhibitors against MRSA and underscore the need for subsequent cytotoxicity assessment and *in vivo* validation to establish their therapeutic applicability.

## Supporting information

S1 Fig PPS of methanol, ethanol, and ethyl acetate extracts of *E. crassipes* flowers and leaves. (A) Methanol extract of *E. crassipes* flowers (MEECF), ethanol extract of *E. crassipes* flowers (EEECF), and ethyl acetate extract of *E. crassipes* flowers (EAEECF). (B) Methanol extract of *E. crassipes* leaves (MEECL), ethanol extract of *E. crassipes* leaves (EEECL), and ethyl acetate extract of *E. crassipes* leaves (EAEECL).
(TIF)

S2 Fig Fourier transform-infrared spectra (FT-IR) of methanol, ethanol, and ethyl acetate extracts of *E. crassipes* flowers. (A) Methanol extract of *E. crassipes* flowers (MEECF). (B) Ethanol extract of *E. crassipes* flowers (EEECF). (C) Ethyl acetate extract of *E. crassipes* flowers (EAEECF).
(TIF)

S3 Fig Fourier transform-infrared spectra (FT-IR) of methanol, ethanol, and ethyl acetate extracts of *E. crassipes* leaves. (A) Methanol extract of *E. crassipes* leaves (MEECL). (B) Ethanol extract of *E. crassipes* leaves (EEECL). (C) Ethyl acetate extract of *E. crassipes* leaves (EAEECL).
(TIF)

S4 Fig GC-MS chromatograms of metabolite annotation of extracts of *Eichhornia* crassipes (Mart. ) Solms flowers. (A) Methanol extract of *Eichhornia crassipes* flowers (MEECF). (B) Ethanol extract of *Eichhornia crassipes* flowers (EEECF). (C) Ethyl acetate extract of *Eichhornia crassipes* flowers (EAEECF).
(TIF)

S5 Fig GC-MS chromatograms of metabolite annotation of extracts of *Eichhornia* crassipes (Mart. ) Solms leaves. (A) Methanol extract of *Eichhornia crassipes* flowers (MEECF). (B) Ethanol extract of *Eichhornia crassipes* flowers (EEECF). (C) Ethyl acetate extract of *Eichhornia crassipes* flowers (EAEECF).
(TIF)

S6 Fig The MIC and MBC of MEECF in µg/mL. (A) The MIC of MEECF and MEECL in µg/mL. (B) The MBC of MEECF and MEECL in µg/mL.
(TIF)

S7 Fig The MIC and MBC of MEECL in µg/mL. (A) The MIC of MEECF and MEECL in µg/mL. (B) The MBC of MEECF and MEECL in µg/mL.
(TIF)

S8 Fig The MIC and MBC of EEECF in µg/mL. (A) The MIC of EEECF and EEECL in µg/mL. (B) The MBC of EEECF and EEECL in µg/mL.
(TIF)

S9 Fig The MIC and MBC of EEECL in µg/mL. (A) The MIC of EEECF and EEECL in µg/mL. (B) The MBC of EEECF and EEECL in µg/mL.
(TIF)

S10 Fig The MIC and MBC of EAEECF in µg/mL. (A) The MIC of EEECF and EEECL in µg/mL. (B) The MBC of EEECF and EEECL in µg/mL.
(TIF)

S11 Fig The MIC and MBC of EAEECL in µg/mL. (A) The MIC of EEECF and EEECL in µg/mL. (B) The MBC of EEECF and EEECL in µg/mL.
(TIF)

S1 Table Summary of molecular docking and dynamics simulation parameters. (DOCX)

S2 Table GC-MS identified phytochemicals in the methanol extract of *Eichhornia crassipes* flower (MEECF). (DOCX)

S3 Table GC-MS identified phytochemicals in the ethanol extract of *Eichhornia crassipes* flower (EEECF). (DOCX)

S4 Table GC-MS identified phytochemicals in the Ethyl acetate extract of *Eichhornia crassipes* flower (EAEECF). (DOCX)

S5 Table GC-MS identified phytochemicals in the methanol extract of *Eichhornia crassipes* leaves (MEECL). (DOCX)

S6 Table GC-MS identified phytochemicals in the ethanol extract of *Eichhornia crassipes* leaves (EEECL). (DOCX)

S7 Table GC-MS identified phytochemicals in the ethyl acetate extract of *Eichhornia crassipes* leaves (EAEECL). (DOCX)

S8 Table Protein ligand interaction analysis of β-lactamase and PBP2a proteins with CID 4970, CID 615944 and control CID 6087. (DOCX)

## Abbreviations

BBB permeability: Blood-Brain Barrier Permeability
EAEECL: Ethyl Acetate Extract of *Eichhornia crassipes* Leaf
EAEECF: Ethyl Acetate Extract of *Eichhornia crassipes* Flower
EEECL: Ethanol Extract of *Eichhornia crassipes* Leaf
EEECF: Ethanol Extract of *Eichhornia crassipes* Flower
*E. crassipes*: *Eichhornia crassipes*
FT-IR: Fourier Transform Infrared Spectroscopy
GC-MS: Gas Chromatography-Mass Spectrometry
GI absorption: Gastrointestinal Absorption
HBA: Hydrogen Bond Acceptors
HBD: Hydrogen Bond Donors
JUST: Jashore University of Science and Technology
MBC: Minimum Bactericidal Concentration
MDR: Multidrug-Resistant
MD simulations: Molecular Dynamics Simulations
MEECF: Methanol Extract of *Eichhornia crassipes* Flower
MEECL: Methanol Extract of *Eichhornia crassipes* Leaf
MIC: Minimum Inhibitory Concentration
MRSA: Methicillin-Resistant *Staphylococcus aureus*
MSSA: Methicillin-Sensitive *Staphylococcus aureus*
MW: Molecular Weight
NIST: National Institute of Standards and Technology
PDB: Protein Data Bank
PBPs: Penicillin-Binding Proteins
PK: Pharmacokinetics
PPS: Preliminary Phytochemical Screening
RB: Rotatable Bonds
Rg: Radius of Gyration
RMSD: Root Mean Square Deviation
RMSF: Root Mean Square Fluctuation
RT: Retention Time
*S. aureus*: *Staphylococcus aureus*
SASA: Solvent Accessible Surface Area
SD: Standard Deviation
TPSA: Topological Polar Surface Area
WHO: World Health Organization
