# Supplementary figures and images for: Unveiling the bactericidal effects of extracts and phytocompounds from Eichhornia crassipes (Mart.) Solms against methicillin-resistant Staphylococcus aureus (MRSA): An in vitro and in silico approach
Source: PLoS One. 2026 Jun 11;21(6):e0349750. doi: 10.1371/journal.pone.0349750 (PMC13258022; doi:10.1371/journal.pone.0349750)

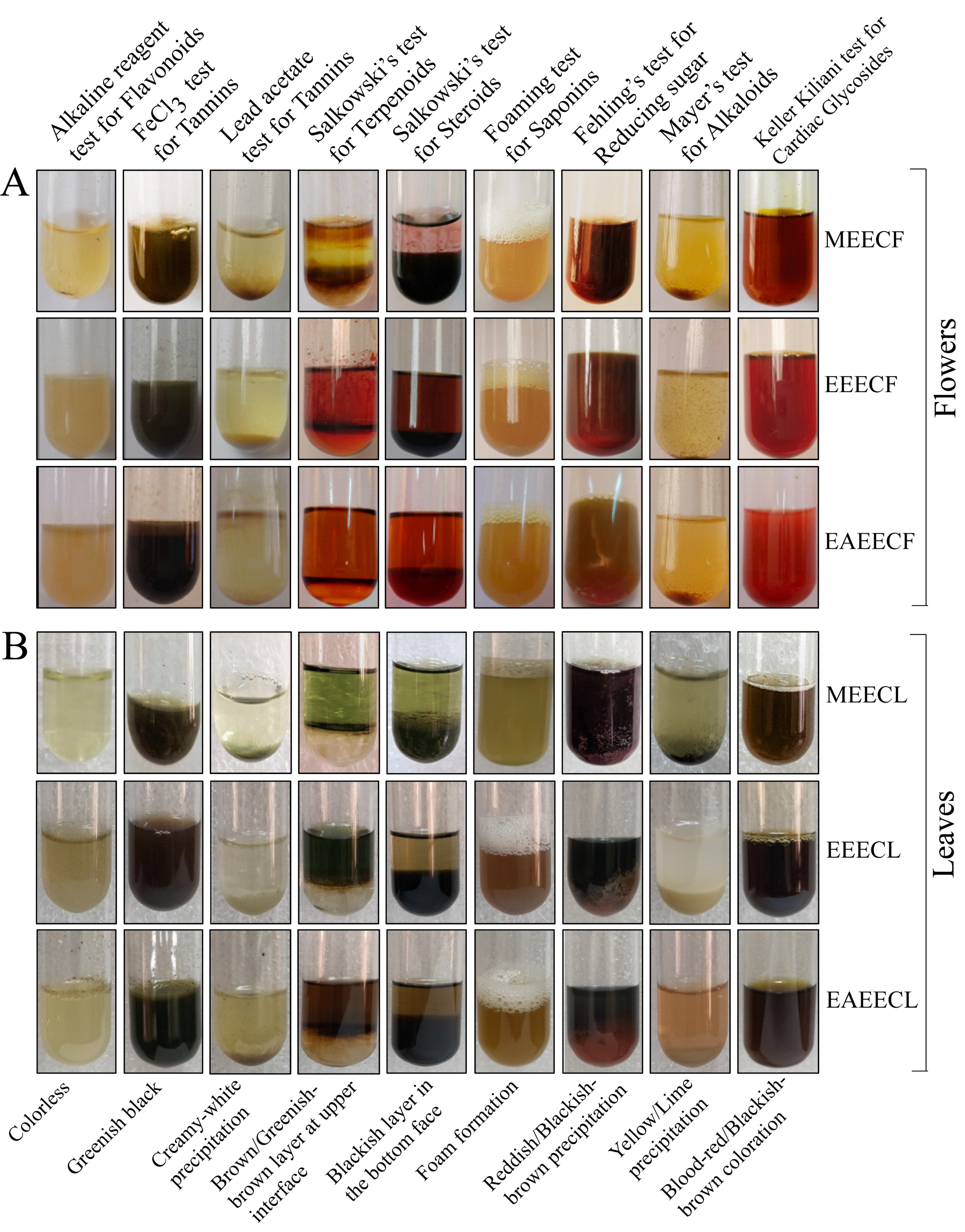

Supplement: S1 Fig — (A) Methanol extract of E. crassipes flowers (MEECF), ethanol extract of E. crassipes flowers (EEECF), and ethyl acetate extract of E. crassipes flowers (EAEECF). (B) Methanol extract of E. crassipes leaves (MEECL), ethanol extract of E. crassipes leaves (EEECL), and ethyl acetate extract of E. crassipes leaves (EAEECL). (TIF) [file pone.0349750.s001.tif]

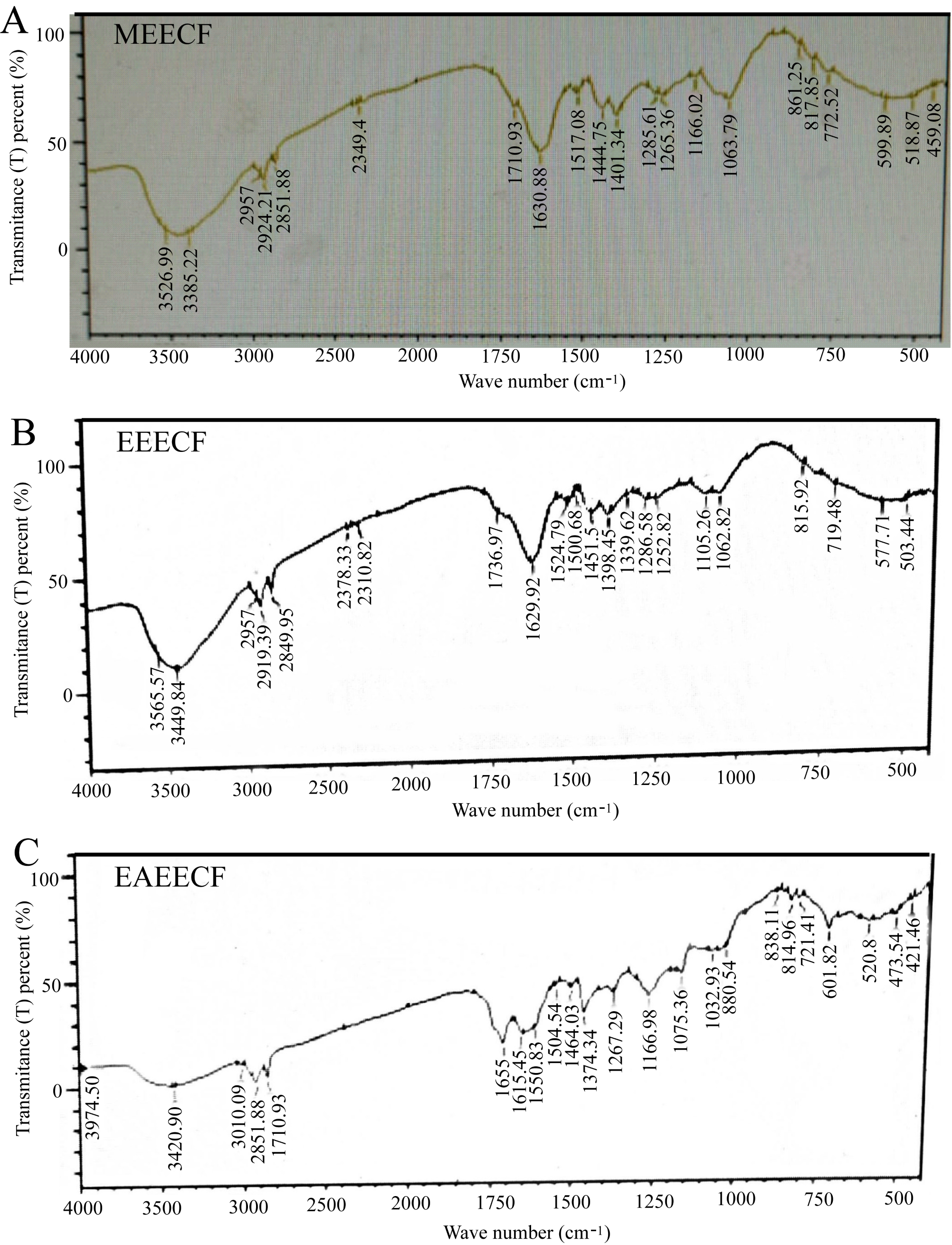

Supplement: S2 Fig — (A) Methanol extract of E. crassipes flowers (MEECF). (B) Ethanol extract of E. crassipes flowers (EEECF). (C) Ethyl acetate extract of E. crassipes flowers (EAEECF). (TIF) [file pone.0349750.s002.tif]

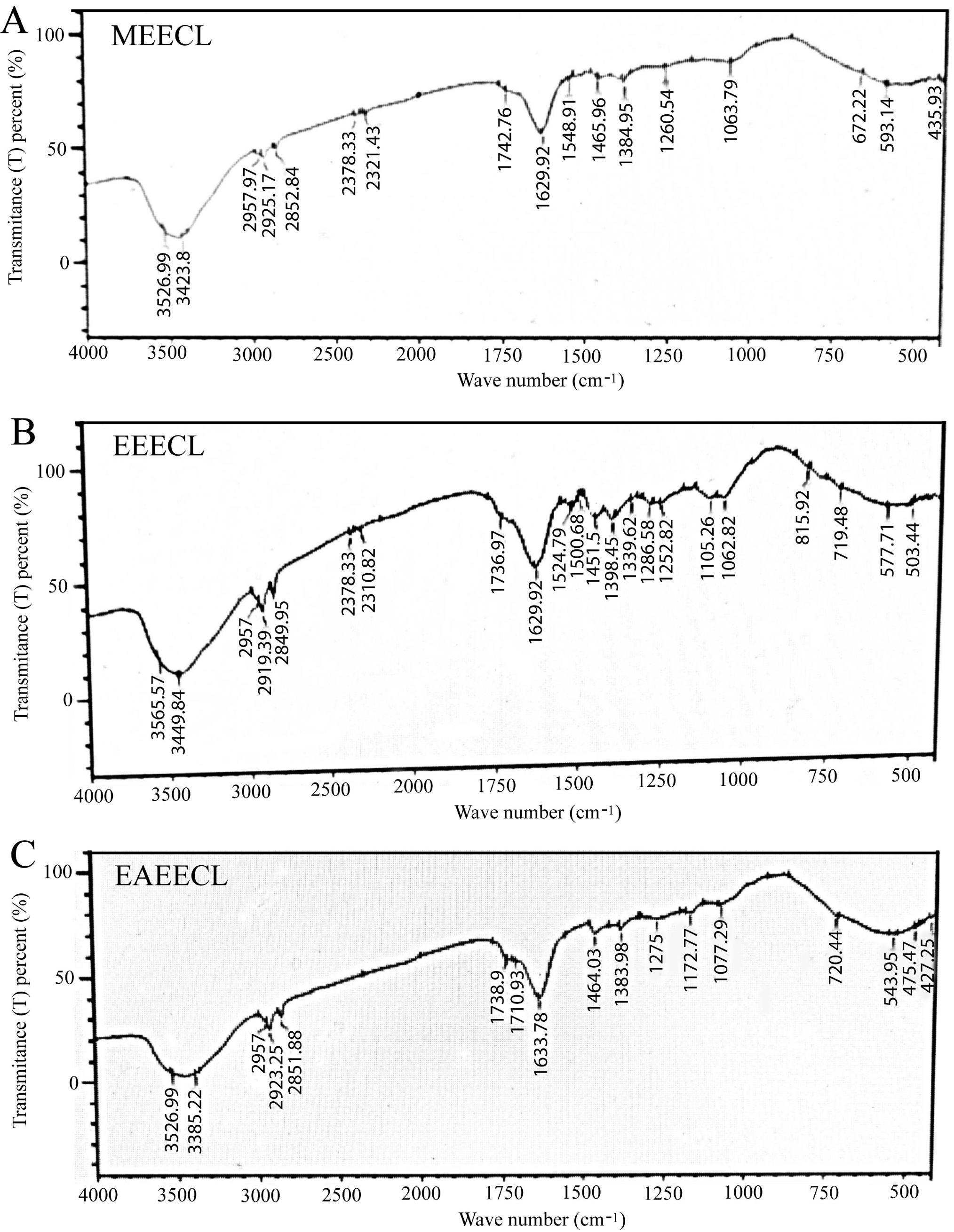

Supplement: S3 Fig — (A) Methanol extract of E. crassipes leaves (MEECL). (B) Ethanol extract of E. crassipes leaves (EEECL). (C) Ethyl acetate extract of E. crassipes leaves (EAEECL). (TIF) [file pone.0349750.s003.tif]

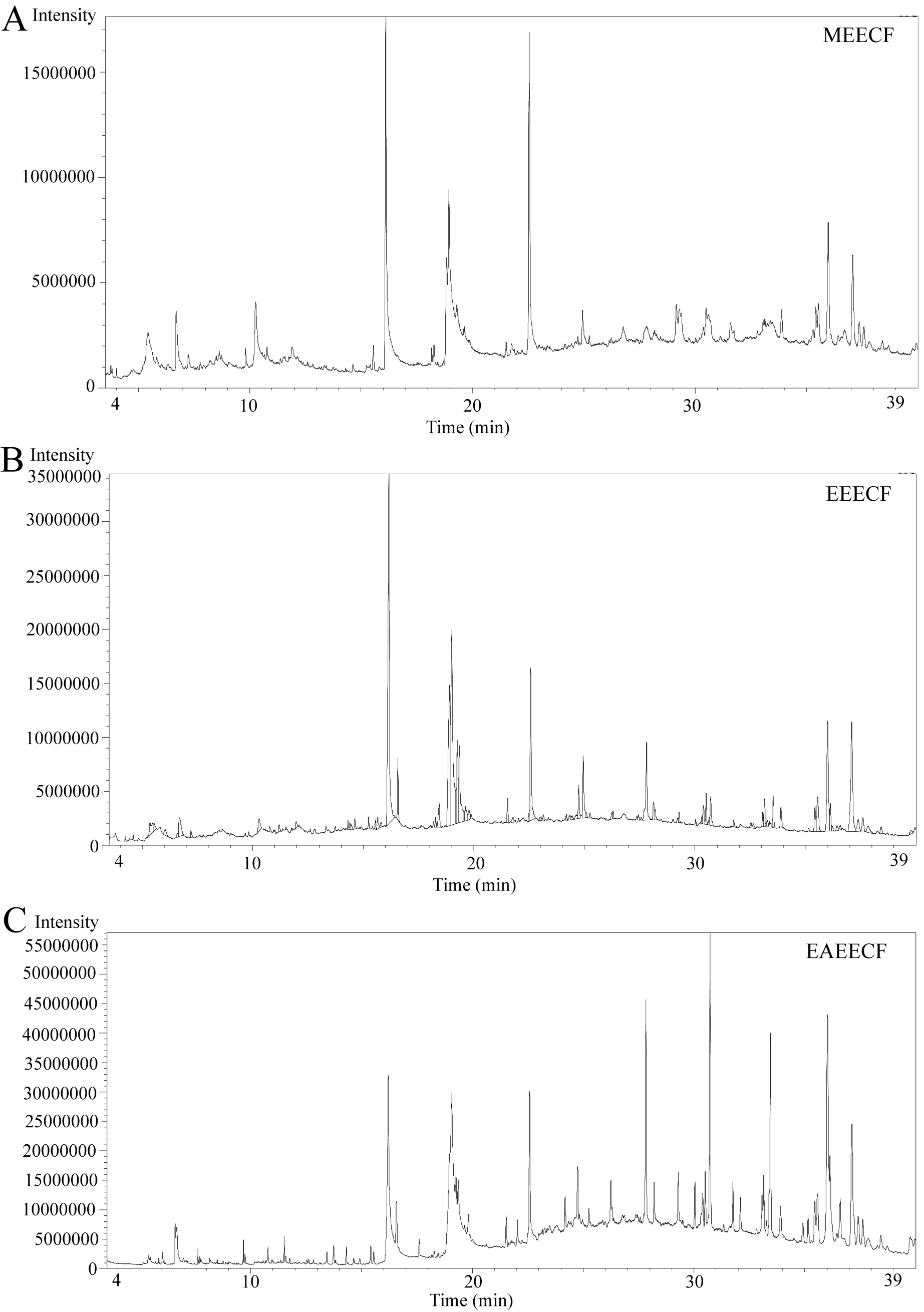

Supplement: S4 Fig — ) Solms flowers. (A) Methanol extract of Eichhornia crassipes flowers (MEECF). (B) Ethanol extract of Eichhornia crassipes flowers (EEECF). (C) Ethyl acetate extract of Eichhornia crassipes flowers (EAEECF). (TIF) [file pone.0349750.s004.tif]

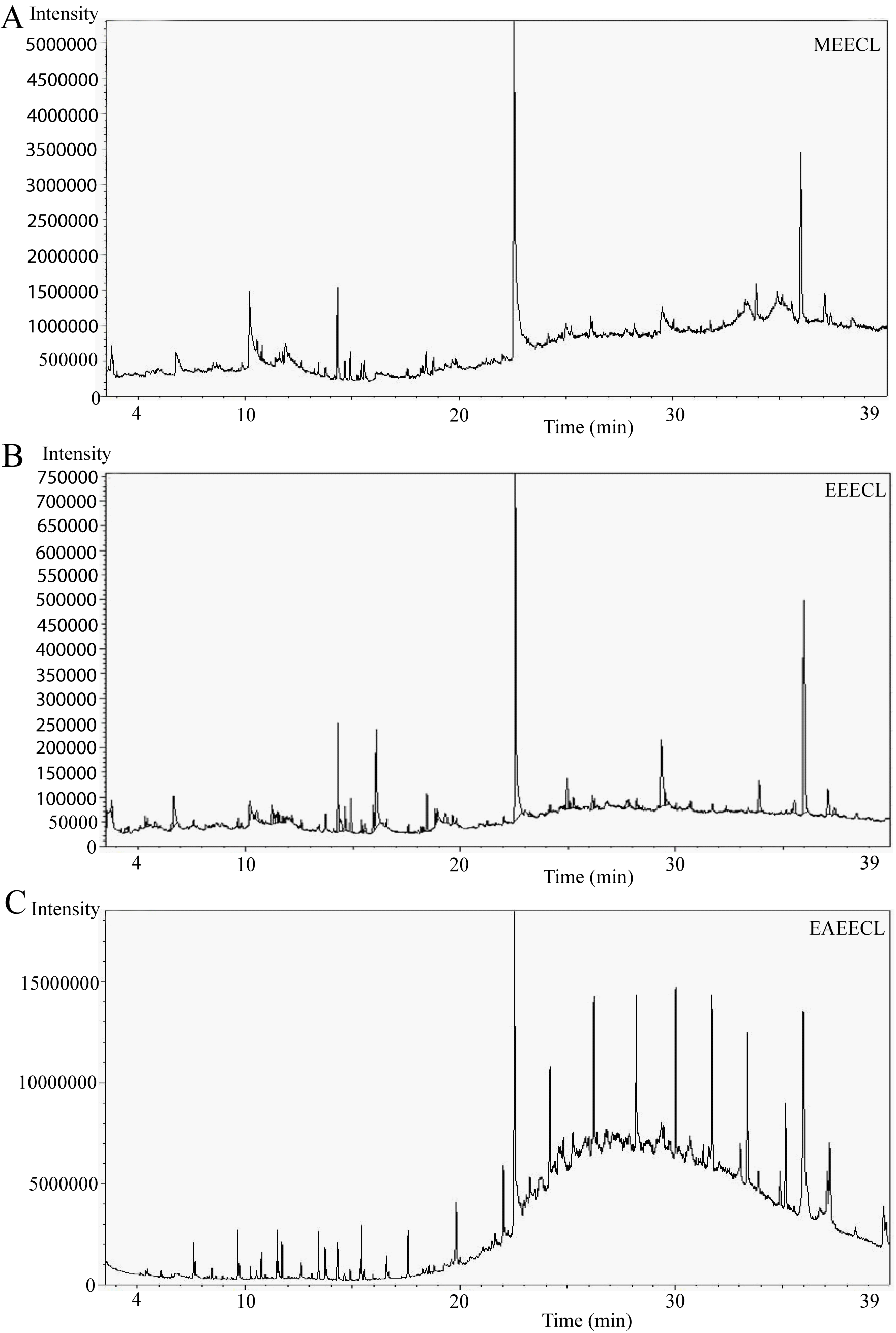

Supplement: S5 Fig — ) Solms leaves. (A) Methanol extract of Eichhornia crassipes flowers (MEECF). (B) Ethanol extract of Eichhornia crassipes flowers (EEECF). (C) Ethyl acetate extract of Eichhornia crassipes flowers (EAEECF). (TIF) [file pone.0349750.s005.tif]

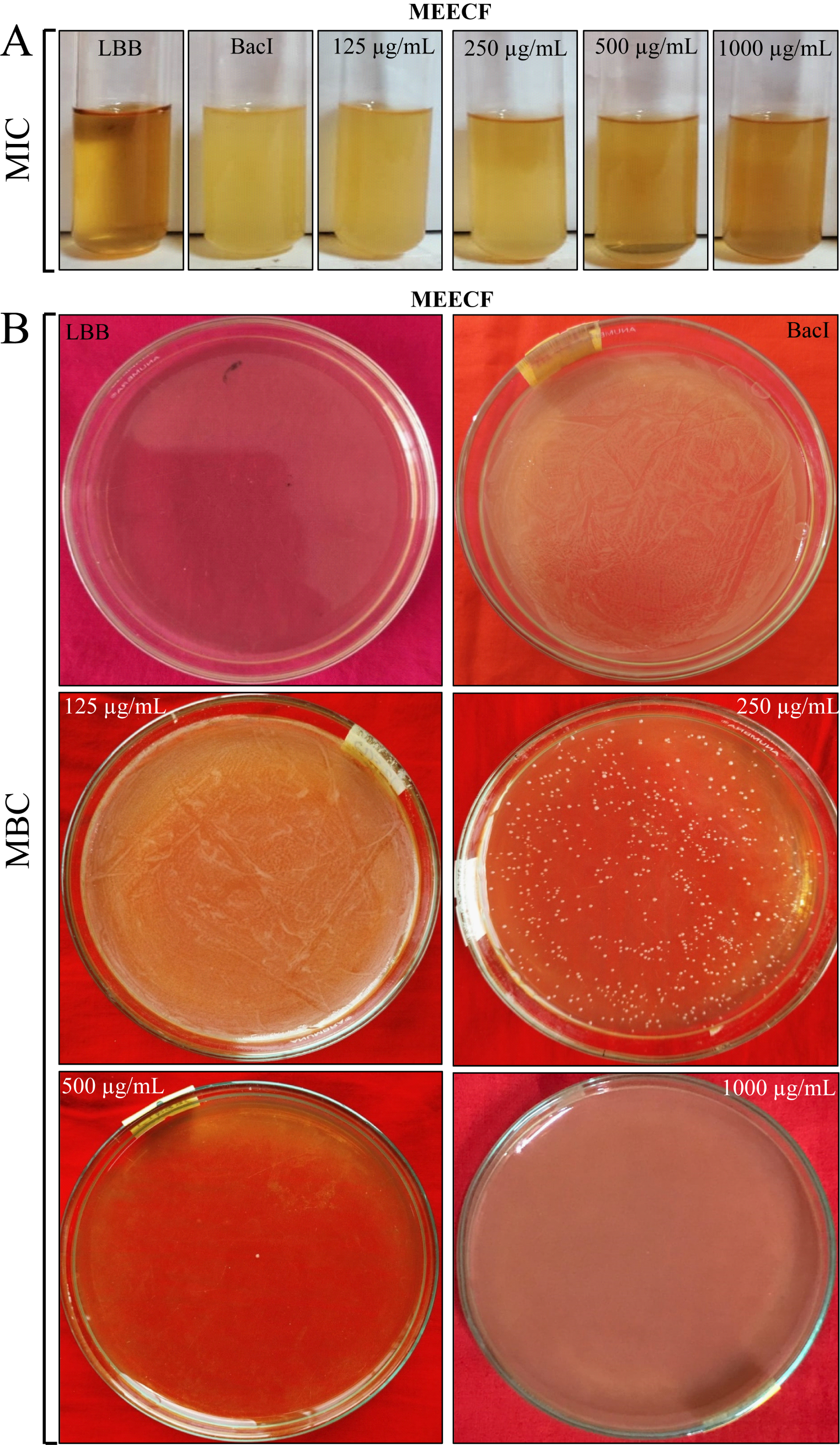

Supplement: S6 Fig — (A) The MIC of MEECF and MEECL in µg/mL. (B) The MBC of MEECF and MEECL in µg/mL. (TIF) [file pone.0349750.s006.tif]

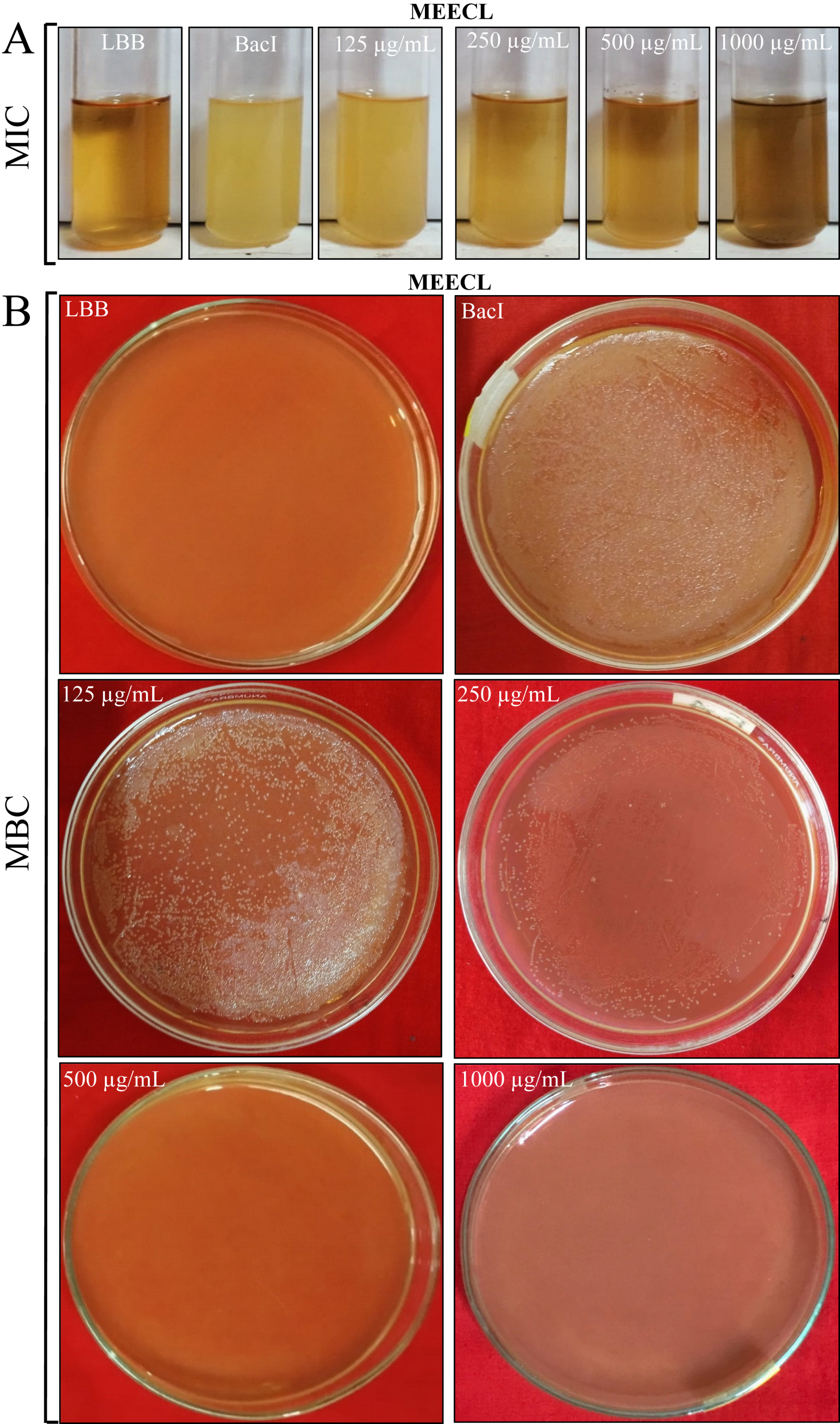

Supplement: S7 Fig — (A) The MIC of MEECF and MEECL in µg/mL. (B) The MBC of MEECF and MEECL in µg/mL. (TIF) [file pone.0349750.s007.tif]

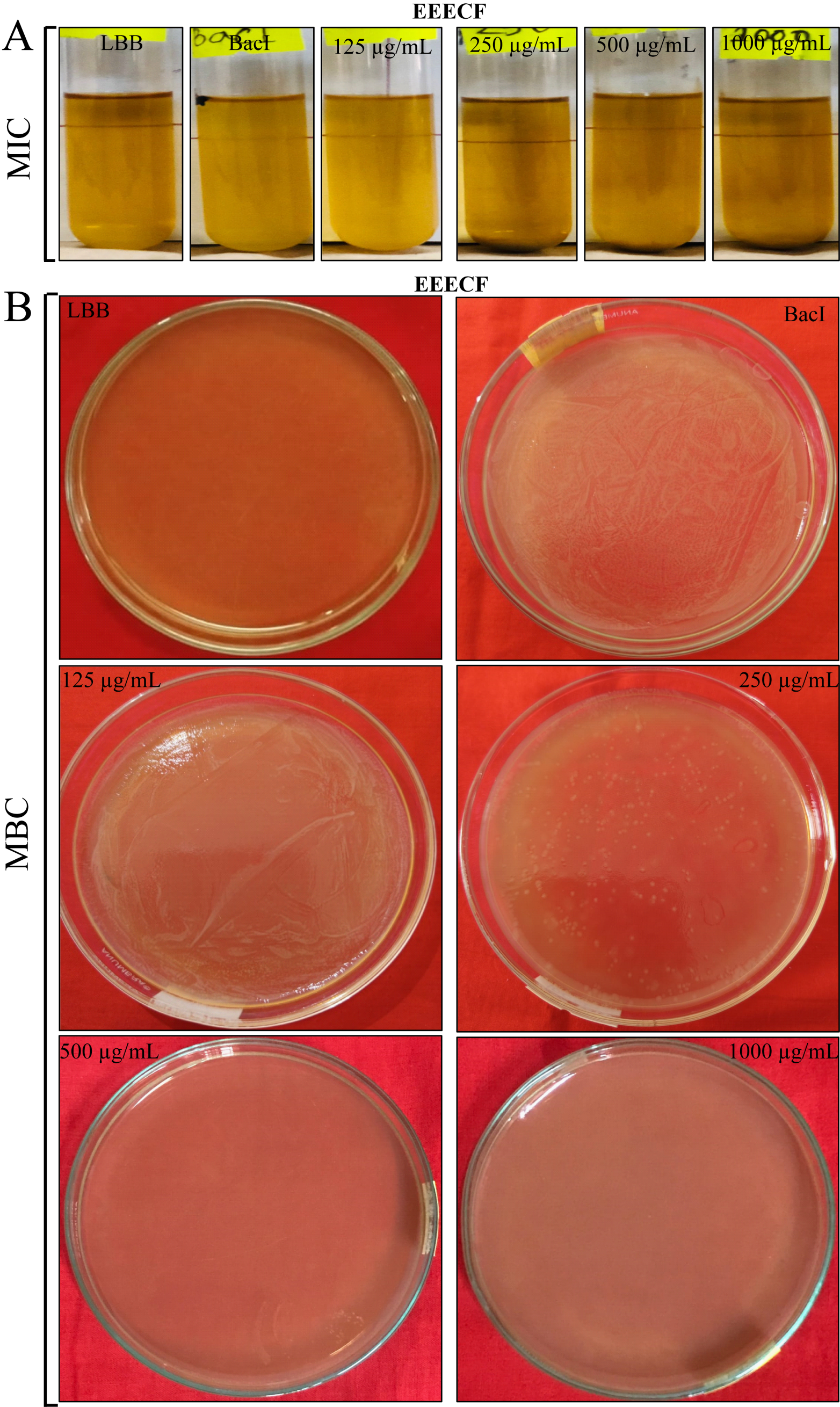

Supplement: S8 Fig — (A) The MIC of EEECF and EEECL in µg/mL. (B) The MBC of EEECF and EEECL in µg/mL. (TIF) [file pone.0349750.s008.tif]

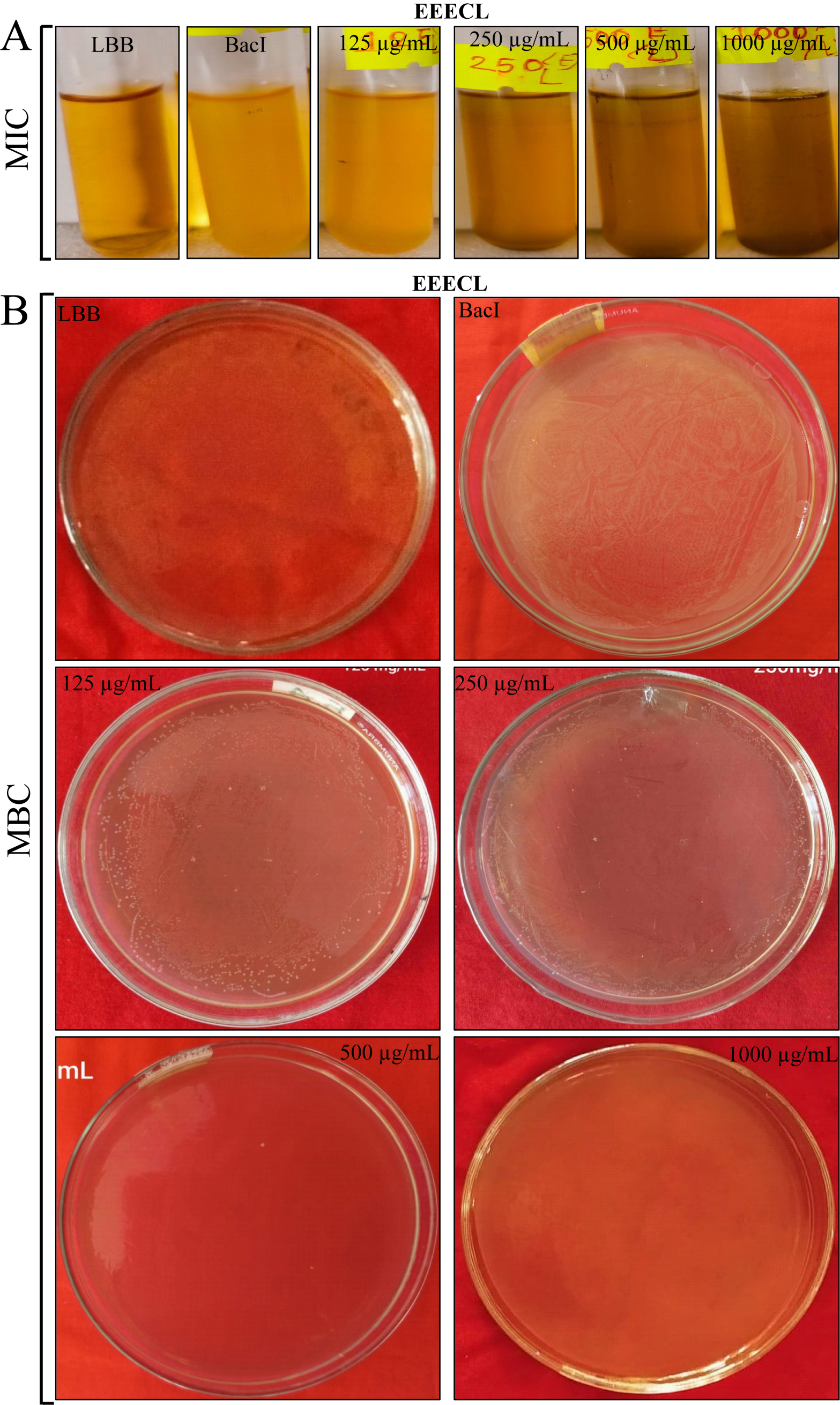

Supplement: S9 Fig — (A) The MIC of EEECF and EEECL in µg/mL. (B) The MBC of EEECF and EEECL in µg/mL. (TIF) [file pone.0349750.s009.tif]

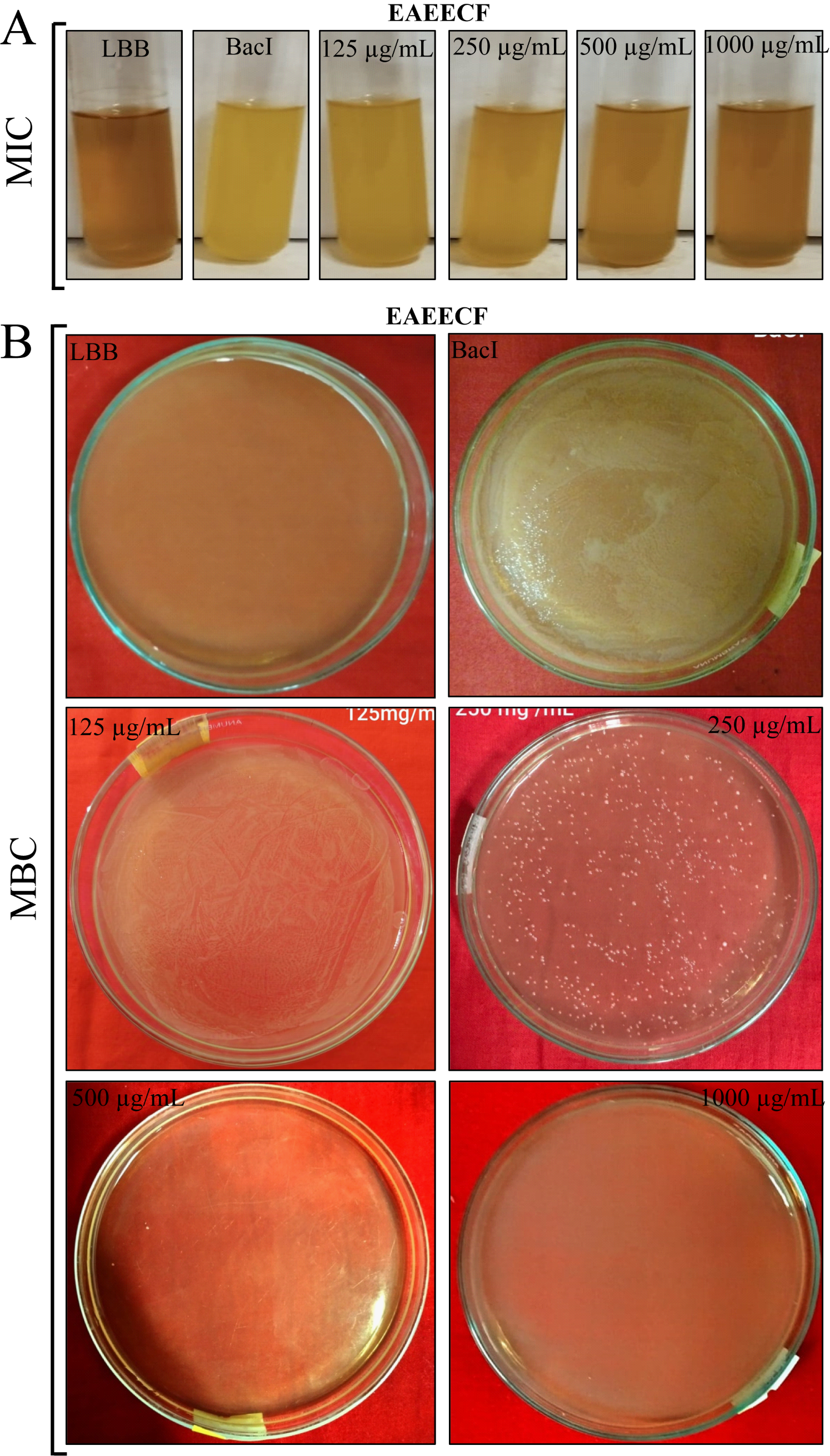

Supplement: S10 Fig — (A) The MIC of EEECF and EEECL in µg/mL. (B) The MBC of EEECF and EEECL in µg/mL. (TIF) [file pone.0349750.s010.tif]

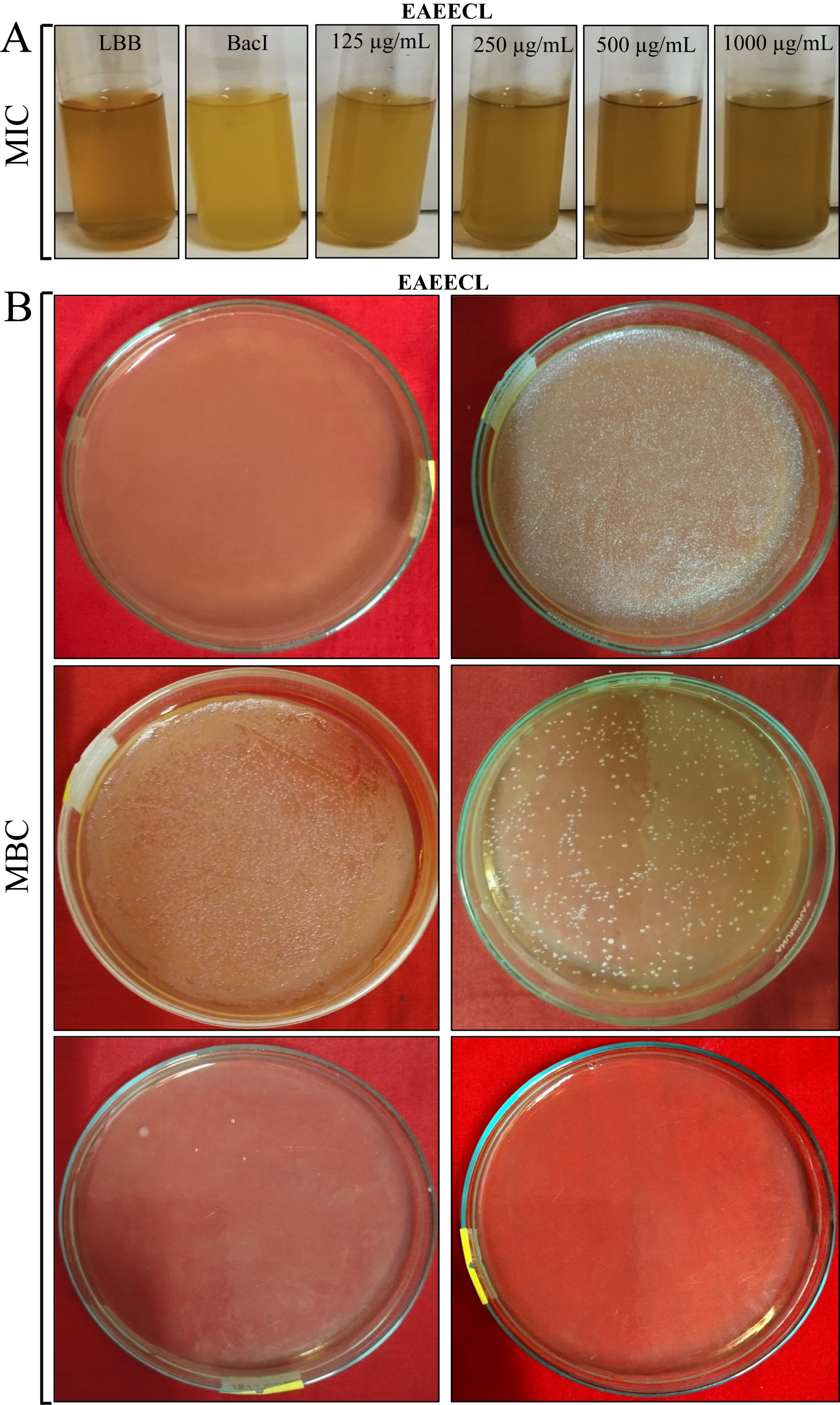

Supplement: S11 Fig — (A) The MIC of EEECF and EEECL in µg/mL. (B) The MBC of EEECF and EEECL in µg/mL. (TIF) [file pone.0349750.s011.tif]
