# Supplementary material for: Unveiling the bactericidal effects of extracts and phytocompounds from Eichhornia crassipes (Mart.) Solms against methicillin-resistant Staphylococcus aureus (MRSA): An in vitro and in silico approach
Source: PLoS One. 2026 Jun 11;21(6):e0349750. doi: 10.1371/journal.pone.0349750 (PMC13258022; doi:10.1371/journal.pone.0349750)
Supplement: S1 Table — (DOCX) [file pone.0349750.s012.docx]

**S1 Table.** Summary of molecular docking and dynamics simulation parameters.

| **Parameter** | **Molecular Docking** | **MD Simulation** |
| --- | --- | --- |
| Software | PyRx (AutoDock Vina) | Schrödinger Desmond |
| Force field | AutoDock default (Vina scoring) | OPLS3e |
| Grid box size | 40 × 40 × 40 Å³ | 10 × 10 × 10 Å³ box |
| Exhaustiveness | 8 | N/A |
| Energy range | 4 kcal/mol | N/A |
| Simulation time | N/A | 200 ns |
| Integration step | N/A | 2 fs |
| Temperature | N/A | 300 K |
| Pressure | N/A | 1.01 bar |
| Solvent model | N/A | SPC water |
| Salt concentration | N/A | 0.15 M NaCl |
