# Supplementary material for: Unveiling the bactericidal effects of extracts and phytocompounds from Eichhornia crassipes (Mart.) Solms against methicillin-resistant Staphylococcus aureus (MRSA): An in vitro and in silico approach
Source: PLoS One. 2026 Jun 11;21(6):e0349750. doi: 10.1371/journal.pone.0349750 (PMC13258022; doi:10.1371/journal.pone.0349750)
Supplement: S2 Table — (DOCX) [file pone.0349750.s013.docx]

**S2 Table**. GC-MS identified phytochemicals in the methanol extract of *Eichhornia crassipes* flower (MEECF).

| **Peak no.** | **Name and formula of the phytochemicals** | **Retention  time** | **Area %** | **Compound CID** | **Nature of**  **phytochemicals** |
| --- | --- | --- | --- | --- | --- |
| 1. | Sucrose (C_12_H_22_O_11_) | 10.187 | 1.034126 | 5988 | Disaccharide |
| 2. | Phenol, 3,5-bis(1,1-dimethylethyl)- (C_14_H_22_O) | 10.781 | 0.271458 | 70825 | Phenol |
| 3. | Neophytadiene (C_20_H_38_O) | 14.313 | 0.581696 | 10446 | Acyclic sesquiterpene |
| 4. | Hexadecanoic acid, methyl ester (C_17_H_34_O_2_) | 15.568 | 0.736815 | 8181 | Ester |
| 5. | 9,12-Octadecadienoic acid, methyl ester (C_19_H_34_O_2_) | 18.185 | 0.736815 | 5284421 | Methyl linoleate |
| 6. | 9,12,15-Octadecatrienoic acid, methyl ester, (Z,Z,Z)- (C_24_H_40_O_4_) | 18.277 | 3.179938 | 5319706 | Fatty acid methyl ester |
| 7. | Phytol (C_20_H_40_O) | 18.432 | 1.848501 | 5280435 | Alcohol |
| 8. | 2,8,9-Trioxa-5-aza-1-silabicyclo[3.3.3]undecane, 1-ethyl- (C_8_H_17_NO_3_Si) | 18.799 | 0.594623 | 74983 | Saturated fatty acid |
| 9. | Octadecanamide (C_18_H_37_NO) | 19.639 | 0.400724 | 31292 | - |
| 10. | 1,1,1,5,7,7,7-Heptamethyl-3,3-bis(trimethylsiloxy)tetrasiloxane (C_13_H_39_O_5_Si_6_) | 19.814 | 0.749741 | 6329081 | - |
| 11. | Tetrapentacontane, 1,54-dibromo- (C_54_H_108_Br_2_) | 21.201 | 0.349018 | 545963 | - |
| 12. | Eicosane (C_20_H_42_) | 21.493 | 0.478283 | 8222 | Alkane |
| 13. | 2-Bromotetradecane (C_14_H_29_Br) | 21.631 | 0.568769 | 12798926 | Alkane |
| 14. | 9-Octadecenamide, (Z)- (C_18_H_35_NO) | 22.552 | 26.13754 | 5283387 | Amide |
| 15. | Cyclopentadecanone, 2-methyl- (C_16_H_30_N) | 22.79 | 2.520683 | 543427 | Ketone |
| 16. | 6-Acetoxy-4-methyl-hept-4-enoic acid (C_10_H_16_O_4_) | 22.915 | 0.52999 | 5371432 | Carboxylic acid |
| 17. | erythro-7,8-Bromochlorodisparlure (C_19_H_38_BrCl) | 22.979 | 0.94364 | 537616 | - |
| 18. | 3-Methyl-8-oxabicyclo[3.2.1]oct-6-en-3-ol (C_8_H_12_O_2_) | 23 | 0.45243 | 557597 | Alcohol |
| 19. | 2-Octynoic acid, methyl ester (C_9_H_14_O_2_) | 23.03 | 0.258532 | 8092 | Ester |
| 20. | 2-methyloctacosane (C2_9_H_60_) | 23.236 | 1.447777 | 519147 | - |
| 21. | 3-Buten-2-ol, 2-methyl-4-(1,3,3-trimethyl-7-oxabicyclo[4.1.0]hept-2-yl)- (C_14_H_24_O_2_) | 23.315 | 0.478283 | 5363622 | Alcohol |
| 22. | Acetic acid, 4-t-butyl-4-hydroxy-1,5-dimethyl-hex-2-ynyl ester (C_14_H_24_O_3_) | 23.37 | 0.52999 | 579166 | Ester |
| 23. | 9,19-Cyclolanostan-3-ol, 24,24-epoxymethano-, acetate (C_33_H_54_O_3_) | 23.41 | 0.336091 | 545556 | Alcohol |
| 24. | Ethyl hexatriacontyl ether (C_38_H_78_O) | 23.49 | 0.400724 | 91693231 | Ether |
| 25. | Phytylhexadecanoate (C_36_H_70_O_2_) | 23.545 | 0.41365 | 14486557 | Ester |
| 26. | 9-Desoxo-9-x-acetoxy-3,8,12-tri-O-acetylingol (C_28_H_40_O_10_) | 23.62 | 0.374871 | 537583 | - |
| 27. | Pipobroman (C_36_H_70_O_2_) | 23.756 | 0.439504 | 4842 | - |
| 28. | Triacontane, 1,30-dibromo- (C_14_H_29_Br) | 23.825 | 0.801448 | 545601 | Alkane |
| 29. | Isopropyl tetracosyl ether (C_27_H_56_O) | 23.915 | 0.297311 | 91691500 | Ether |
| 30. | Propyleneglycol monoleate (C_21_H_40_O_3_) | 23.98 | 0.504137 | 5365625 | Ester |
| 31. | Oxirane, 2-butyl-3-methyl-, cis- (C_7_H_14_O) | 24.236 | 0.698035 | 9898811 | Epoxide |
| 32. | 9-Oxabicyclo[6.1.0]nonan-4-ol  (C_8_H_14_O_2_) | 24.355 | 0.659255 | 273831 | Alcohol |
| 33. | 7-Methyl-Z-tetradecen-1-ol acetate (C_17_H_32_O_2_) | 24.4 | 0.323164 | 5363222 | Alcohol |
| 34. | Fumaric acid, but-3-yn-2-yl undecyl ester (C_19_H_30_O_4_) | 24.431 | 0.853154 | 91701146 | Ester |
| 35. | 11-Methyltricosane (C_24_H_50_) | 24.555 | 1.370217 | 530326 | Alkane |
| 36. | Docosyl heptafluorobutyrate (C_26_H_45_F_7_O_2_) | 24.64 | 0.775595 | 91693307 | Ester |
| 37. | Octatriacontyl pentafluoropropionate (C_41_H_77_F_5_O_2_) | 24.725 | 1.279731 | 91693082 | Fluorinated carboxylic acid |
| 38. | Nonadecane (C_19_H_40_) | 24.818 | 1.253878 | 12401 | Alkane |
| 39. | 2-Exo-hydroxy-5-ketobornane (C_10_H_16_O_2_) | 24.865 | 0.478283 | 189214 | - |
| 40. | Methyl-2,3:4,6-di-O-furylidene.alpha.d-mannopyranoside (C_17_H_18_O_8_) | 24.94 | 0.555843 | 91697695 | Carbohydrate |
| 41. | 7-Hexadecenal, (Z)- (C_16_H_30_O) | 24.994 | 0.840228 | 5364438 | Aldehyde |
| 42. | 1-Hexadecanesulfonic acid, 3,5-dichloro-2,6-dimethyl-4-pyridyl ester (C_23_H_39_Cl_2_NO_3_S) | 25.07 | 0.646329 | 536380 | Ester |
| 43. | 9-(2',2'-Dimethylpropanoilhydrazono)-3,6-dichloro-2,7-bis-[2-(diethylamino)-ethoxy]fluorene (C_30_H_42_Cl_2_NO_4_S_3_) | 25.175 | 0.956567 | 590814 | - |
| 44. | 13-Methyl-Z-14-nonacosene (C_30_H_60_) | 25.248 | 0.698035 | 5365104 | Alkane |
| 45. | Cyclopentane, 1,1'-[3-(2-cyclopentylethyl)-1,5-pentanediyl]bis- (C_22_H_40_) | 25.32 | 0.310238 | 281840 | - |
| 46. | Eicosane, 2-methyl- (C_21_H_44_) | 25.36 | 0.853154 | 519146 | Alkane |
| 47. | Octane, 2-bromo- (C_8_H_17_Br) | 25.42 | 0.41365 | 79046 | Bromoalkane |
| 48. | Chloromethyl 8-chlorodecanoate (C_11_H_20_Cl_2_O_2_) | 25.49 | 1.124612 | 543787 | - |
| 49. | Cyclopentanemethanamine, 5-amino-2,2,4-trimethyl- (C_9_H_20_N_2_) | 25.54 | 0.723888 | 106962 | Amine |
| 50. | 2-Cyclohexen-1-one, 3-(3-hydroxybutyl)-2,4,4-trimethyl- (C_13_H_22_O_2_) | 25.755 | 0.374871 | 520295 | Ketone |
| 51. | 2-Piperidinone, N-[4-bromo-n-butyl]- (C_9_H_16_BrNO) | 25.809 | 0.866081 | 536377 | Ketone |
| 52. | Tetratriacontyl heptafluorobutyrate (C_38_H_69_F_7_O_2_) | 25.86 | 0.90486 | 91692919 | Ester |
| 53. | Triacontane, 1-bromo- (C_30_H_61_Br) | 25.956 | 0.891934 | 521082 | Bromoalkane |
| 54. | Carbonic acid, decyl hexadecyl ester (C_27_H_54_O_3_) | 26.359 | 0.607549 | 91693144 | Ester |
| 55. | Trichloroacetic acid, 4-hexadecyl ester (C_18_H_33_Cl_3_O_2_) | 26.753 | 0.439504 | 544057 | Ester |
| 56. | 5,8-Tridecadione (C_13_H_24_O_2_) | 26.829 | 0.504137 | 538300 | Ketone |
| 57. | 2(1H)-Naphthalenone, octahydro-4a-methyl-7-(1-methylethyl)-, (4a.alpha.,7.beta.,8a.beta.)- (C_14_H_24_O) | 27.054 | 0.387797 | 41133 | Ketone |
| 58. | Geranyl isovalerate (C_15_H_26_O_2_) | 27.155 | 0.426577 | 5362830 | Ester |
| 59. | 2-Methyltetracosane (C_25_H_52_) | 27.215 | 0.478283 | 527459 | Alkane |
| 60. | Docosane, 1,22-dibromo- (C_22_H_44_Br_2_) | 29.152 | 0.426577 | 545960 | Alkane |
| 61. | Octadecane, 3-ethyl-5-(2-ethylbutyl)- (C_26_H_54_) | 29.269 | 0.504137 | 292285 | Alkane |
| 62. | 2-Hydroxymethyl-2,6,8,8-tetramethyltricyclo[5.2.2.0(1,6)]undecane (C_16_H_28_O) | 29.754 | 0.297311 | 591157 | Alcohol |
| 63. | 2,3:5,6-Di-O-1-Cyclohexylieden-1,4-cyclohexandiallylether  (C_24_H_36_O_6_) | 29.938 | 0.49121 | 15704917 | Ester |
| 64. | Eicosyl heptafluorobutyrate (C_24_H_41_F_7_O_2_) | 30.7 | 0.620476 | 91693308 | - |
| 65. | Cholestan-3-ol, methyl carbonodithioate, (3.beta.,5.alpha.)- (C_29_H_50_OS_2_) | 30.781 | 0.336091 | 11016296 | - |
| 66. | 1-Dimethyl(3-chloropropyl)silyloxydodecane (C_17_H_37_ClOSi) | 31.164 | 0.620476 | 554879 | Siloxane |
| 67. | Allocryptopine (C_21_H_23_NO_5_) | 31.601 | 2.805067 | 98570 | Alkaloid |
| 68. | Protopine (C_20_H_19_NO_5_) | 32.526 | 1.977766 | 4970 | Alkaloid |
| 69. | 1,30-Triacontanediol (C_30_H_62_O_2_) | 32.645 | 0.775595 | 543982 | Alcohol |
| 70. | Octatriacontyl trifluoroacetate (C_40_H_77_F_3_O_2_) | 33.293 | 1.913133 | 91693163 | Ester |
| 71. | Vitamin E (C_29_H_50_O_2_) | 33.533 | 1.796794 | 14985 | Fat soluble vitamin |
| 72. | 1,25-Dihydroxyvitamin D3, TMS derivative (C_30_H_52_O_3_Si) | 33.897 | 0.439504 | 5364601 | Fat soluble vitamin |
| 73. | Cholest-5-en-3-ol, (3.alpha.)-, TMS derivative (C_30_H_54_OSi) | 36.539 | 17.43795 | 22211625 | Alcohol |
| 74. | Phenol, 2,4-bis(1,1-dimethylethyl)-, phosphite (3:1) (C_45_H_69_O_3_P) | 37.069 | 0.465357 | 111787 | Ester |
