# Supplementary material for: Unveiling the bactericidal effects of extracts and phytocompounds from Eichhornia crassipes (Mart.) Solms against methicillin-resistant Staphylococcus aureus (MRSA): An in vitro and in silico approach
Source: PLoS One. 2026 Jun 11;21(6):e0349750. doi: 10.1371/journal.pone.0349750 (PMC13258022; doi:10.1371/journal.pone.0349750)
Supplement: S3 Table — (DOCX) [file pone.0349750.s014.docx]

**S3 Table.** GC-MS identified phytochemicals in the ethanol extract of *Eichhornia crassipes* flower (EEECF).

| **Peak no.** | **Structure, name, and formula of the phytochemicals** | **Retention  time** | **Area %** | **Compound CID** | **Nature of phytochemicals** |
| --- | --- | --- | --- | --- | --- |
| 1. | 4-Heptenal, (Z)- (**C_7_H_12_O)** | 5.347 | 0.59 | 5362814 | Aldehyde |
| 2. | Ethanol, 2,2'-[1,2 ethanediylbis(thio)]bis- (C_6_H_14_O_2_S_2_) | 5.475 | 0.52 | 78904 | Dithioethanol / Dithioether |
| 3. | Acetic acid, octyl ester (C_10_H_20_O_2_) | 5.565 | 0.46 | 8164 | Ester |
| 4. | Benzeneacetaldehyde (C_8_H_8_O) | 6.045 | 0.39 | 998 | Aromatic aldehyde |
| 5. | 1-Butanol, 3-methyl-formate(C_10_H_12_O_2_) | 6.677 | 1.39 | 8052 | Ester |
| 6. | 4H-Pyran-4-one, 2,3-dihydro-3,5-dihydroxy-6-methyl- (C_6_H_8_O_4_) | 7.205 | 0.22 | 119838 | Pyronine |
| 7. |  | 10.295 | 0.81 |  | Sugar alcohol/ Polyol |
| 8. | d-Gluco-heptulosan (C_7_H_14_O_7_) | 10.767 | 0.20 | 548228 | Monosaccharide |
| 9. | Ethanol, 2-[2-(2-methoxyethoxy) ethoxy]- (C_7_H_16_O_4_) | 11.187 | 0.25 | 8178 | Ether |
| 10. | Ethyl. alpha.-d-glucopyranoside (C_8_H_16_O_6_) | 11.964 | 0.30 | 91694274 | Glycoside |
| 11. | Diethyl-.alpha.-naphthylamine (C_14_H_17_N) | 12.785 | 0.17 | 66547 | Aromatic amine |
| 12. | Neophytadiene (C_20_H_34_) | 14.307 | 0.21 | 10446 | Terpene |
| 13. | 2,5,8,11,14-Pentaoxahexadecan-16-ol (C_11_H_24_O_6_) | 14.391 | 0.21 | 90255 |  |
| 14. | Tetraglyme  (C_10_H_22_O_5_) | 14.456 | 0.17 | 8925 | Glycol ether |
| 15. | Pentadecanoic acid (C_15_H_30_O_2_) | 14.621 | 0.33 | 13849 | Fatty acid |
| 16. | Hexadecanenitrile (C_16_H_31_N) | 15.235 | 0.30 | 69424 | Fatty acid |
| 17. | Hexadecanoic acid, methyl ester (C_17_H_34_O_2_) | 15.547 | 0.25 | 8181 | Fatty acid ester |
| 18. | Oleyl alcohol, trifluoroacetate (C_20_H_35_F_3_O_2_) | 15.799 | 0.21 | 88368751 | - |
| 19. | n-Hexadecanoic acid (C_16_H_32_O_2_) | 16.157 | 17.17 | 985 | Fatty acid |
| 20. | Hexadecanoic acid, ethyl ester (C_18_H_36_O_2_) | 16.564 | 1.59 | 12366 | Fatty acid ester |
| 21. | 7-Hexadecenal, (Z)- (C_16_H_30_O) | 18.271 | 0.29 | 5364438 | Aldehyde |
| 22. | Phytol (C_20_H_40_O) | 18.434 | 1.06 | 5280435 | Terpenoid |
| 23. | 9,12-Octadecadienoic acid (Z,Z)- (C_18_H_32_O_2_) | 18.893 | 8.68 | 5280450 | Fatty acid |
| 24. | 9,12,15-Octadecatrienoic acid, (Z,Z,Z)- (C_18_H_30_O_2_) | 18.996 | 12.82 | 5280934 | Fatty acid |
| 25. | Thunbergol (C_20_H_34_O) | 19.175 | 0.60 | 5363523 | Diterpene alcohol |
| 26. | Linoleic acid ethyl ester (C_20_H_36_O_2_) | 19.249 | 3.37 | 5282184 | Fatty acid ester |
| 27. | 9,12,15-Octadecatrienoic acid, ethyl ester, (Z,Z,Z)- (C_20_H_34_O_2_) | 19.35 | 3.48 | 5367460 | Fatty acid ester |
| 28. | 2-((2R,4aR,8aS)-4a-Methyl-8-methylenedecahydronaphthalen-2-yl)acrylaldehyde (C_15_H_22_O) | 19.43 | 1.11 | 14262761 | Terpenoid |
| 29. | 1,4-Cyclohexanediol, (Z)-, TMS derivative (C_9_H_20_O_2_Si) | 19.56 | 0.17 | 554465 | - |
| 30. | Octadecanamide (C_18_H_37_NO) | 19.626 | 0.82 | 31292 | Fatty acid amide |
| 31. | Octadecanoic acid, 17-methyl-, methyl ester (C_20_H_40_O_2_) | 19.772 | 0.60 | 554141 | Fatty acid |
| 32. | Decanamide, N-(2-hydroxyethyl)- (C_12_H_25_NO_2_) | 21.765 | 0.27 | 111739 | Amide |
| 33. | 3-Buten-2-ol, 2-methyl-4-(1,3,3-trimethyl-7-oxabicyclo[4.1.0]hept-2-yl)- (C_14_H_24_O_2_) | 22.485 | 0.22 | 5363622 | Terpenoid |
| 34. | 9-Octadecenamide, (Z)- (C_18_H_35_NO) | 22.566 | 5.72 | 5283387 | Amide |
| 35. | Ethyl hydrogen dodecanedioate (C_14_H_26_O_4_) | 23.039 | 0.21 | 560435 | Ester |
| 36. | Oxalic acid, decyl 3,5-difluorophenyl ester (C_18_H_24_F_2_O_4_) | 24.337 | 0.22 | 6420726 | Bile acid |
| 37. | Ethyl iso-allocholate (C_26_H_44_O_5_) | 24.436 | 0.22 | 6452096 | Bile acid |
| 38. | Heptanoic acid, heptyl ester (C_14_H_28_O_2_) | 24.59 | 0.28 | 69350 | Fatty acid ester |
| 39. | Eicosane C_20_H_42_) | 24.742 | 1.07 | 8222 | Aliphatic hydrocarbon |
| 40. | Hexadecanoic acid, 2-hydroxy-1-(hydroxymethyl)ethyl ester C_19_H_38_O_4_) | 24.943 | 2.42 | 123409 | Fatty acid ester |
| 41. | 9-Hexacosene C_26_H_52_) | 27.446 | 0.18 | 5363630 | Hydrocarbon |
| 42. | 2-Methylhexacosane (C_27_H_56_) | 27.809 | 3.58 | 150931 | Hydrocarbon |
| 43. | Octadecanoic acid, 2,3-dihydroxypropyl ester (C_21_H_42_O_4_) | 28.122 | 0.71 | 24699 | Fatty acid ester |
| 44. | 1-Heptacosanol (C_27_H_56_O) | 30.497 | 1.04 | 74822 | Alcohol |
| 45. | Cyclohexane, 1-(cyclohexylmethyl)-2-ethyl-, cis- (C_15_H_28_) | 30.64 | 0.26 | 41236 | - |
| 46. | Tetratetracontane (C_44_H_90_) | 30.708 | 1.14 | 23494 | Aliphatic hydrocarbon |
| 47. | Allocryptopine (C_21_H2_3_NO_5_) | 32.535 | 0.21 | 98570 | Alkaloid |
| 48. | Protopine (C_20_H_19_NO_5_) | 32.634 | 0.22 | **4970** | Alkaloid |
| 49. | Heneicosyl trifluoroacetate (C_23_H_43_F_3_O_2_) | 33.061 | 0.44 | 14574255 | Fatty acid ester |
| 50. | 17-Pentatriacontene (C_35_H_70_) | 33.138 | 0.97 | 5365022 | Aliphatic hydrocarbon |
| 51. | Octatriacontyl trifluoroacetate (C_40_H_77_F_3_O_2_) | 33.54 | 1.00 | 91693163 | Fatty acid ester |
| 52. | 26-Nor-5-cholesten-3.beta.-ol-25 one  (C_26_H_42_O_2_) | 33.885 | 0.89 | 165617 | Sterol |
| 53. | Cholest-5-en-3-ol, 24-propylidene-, (3.beta.)- (C_30_H_50_O) | 35.43 | 1.12 | 6443745 | Sterol |
| 54. | Ergost-5-en-3-ol, (3.beta.)- (C_28_H_48_O) | 35.542 | 1.81 | 5283637 | Sterol |
| 55. | Stigmasterol (C_29_H_48_O) | 35.989 | 5.20 | 5280794 | Phytosterol |
| 56. | Docosyl heptafluorobutyrate (C_26_H_45_F_7_O_2_) | 36.112 | 1.22 | 91693307 | Fatty acid ester |
| 57. | Heptacos-1-ene (C_27_H_54_) | 36.23 | 0.17 | 528971 | Aliphatic hydrocarbon |
| 58. | Cholesta-5,20,24-trien-3-ol, (3.beta.)- (C_27_H_42_O) | 36.427 | 0.23 | 22295535 | Sterol |
| 59. | Ergost-7-en-3-ol (C_28_H_48_O) | 36.563 | 0.20 | 21116218 | Sterol |
| 60. | beta.-Sitosterol (C_29_H_50_O) | 37.087 | 7.20 | 222284 | Phytosterol |
| 61. | Fucosterol (C_29_H_48_O) | 37.372 | 0.84 | 5281328 | Phytosterol |
| 62. | 9,19-Cycloergost-24(28)-en-3-ol, 4,14-dimethyl,acetate,(3.beta.,4.alpha.,5.alpha.)- (C_32_H_52_O_2_) | 37.589 | 0.80 | 537081 | Sterol |
| 63. | 9,19-Cyclolanostan-3-ol, acetate, (3.beta.)- (C_32_H_54_O_2_) | 37.819 | 0.60 | 537304 | Sterol |
| 64. | 4,22-Stigmastadiene-3-one (C_29_H_46_O) | 38.416 | 0.39 | 5364563 | Steroid |
| 65. | Stigmast-4-en-3-one (C_29_H_48_O) | 39.76 | 0.23 | 5484202 | Steroid |
