# Supplementary material for: Unveiling the bactericidal effects of extracts and phytocompounds from Eichhornia crassipes (Mart.) Solms against methicillin-resistant Staphylococcus aureus (MRSA): An in vitro and in silico approach
Source: PLoS One. 2026 Jun 11;21(6):e0349750. doi: 10.1371/journal.pone.0349750 (PMC13258022; doi:10.1371/journal.pone.0349750)
Supplement: S4 Table — (DOCX) [file pone.0349750.s015.docx]

**S4 Table.** GC-MS identified phytochemicals in the Ethyl acetate extract of *Eichhornia crassipes* flower (EAEECF).

| **Peak no.** | **Structure, name, and formula of the phytochemicals** | **Retention  time** | **Area %** | **Compound CID** | **Nature of phytochemicals** |
| --- | --- | --- | --- | --- | --- |
| 1. | 1-Decene (C_10_H_20_) | 5.378 | 0.17 | 13381 | Alkene |
| 2. | 2,4,4-Trimethyl-1-pentanol (C_8_H_18_O) | 6.02 | 0.15 | 85984 | Alcohol |
| 3. | 1,2,3-Propanetriol, 1-acetate (C_5_H_10_O_4_) | 6.588 | 0.55 | 33510 | Alcohol |
| 4. | 1-Butanol, 3-methyl-, formate (C_6_H_12_O_2_) | 6.666 | 1.16 | 8052 | Ester |
| 5. | 1-Dodecene (C_12_H_24_) | 7.615 | 0.15 | 8183 | Alkene |
| 6. | Glycerol 1,2-diacetate (C_7_H_12_O_5_) | 8.152 | 0.08 | 66021 | Alcohol |
| 7. | 1-Tetradecene (C_14_H_28_) | 9.666 | 0.24 | 14260 | Alkene |
| 8. | Hexadecane (C_16_H_34_) | 9.74 | 0.09 | 11006 | Alkane |
| 9. | 1,3-Propanediol, 2-(hydroxymethyl)-2-nitro- (C_4_H_9_NO_5_) | 10.175 | 0.09 | 31337 | - |
| 10. | Undecanoic acid (C_11_H_22_O_2_) | 10.336 | 0.09 | 8180 | Carboxylic acid |
| 11. | E-14-Hexadecenal (C_16_H_30_O) | 11.515 | 0.28 | 5363106 | Aldehyde |
| 12. | 10-Heneicosene (c,t) (C_21_H_42_) | 13.742 | 0.24 | 5364553 | Alkene |
| 13. | Neophytadiene (C_20_H_38_) | 14.315 | 0.21 | 10446 | sesquiterpene |
| 14. | 3,7,11,15-Tetramethyl-2-hexadecen-1-ol (C_20_H_40_O) | 14.646 | 0.09 | 5366244 | Alcohol |
| 15. | Hexadecanoic acid, methyl ester (C_17_H_34_O_2_) | 15.555 | 0.18 | 8181 | Fatty acid |
| 16. | n-Hexadecanoic acid (C_16_H_32_O_2_) | 16.21 | 8.04 | 985 | Saturated fatty acid |
| 17. | Hexadecanoic acid, ethyl ester (C_18_H_36_O_2_) | 16.575 | 1.25 | 12366 | Ester |
| 18. | 2-Methyltetracosane (C_25_H_52_) | 18.277 | 0.12 | 527459 | Alkane |
| 19. | Tetradecanoic acid, 12-methyl-, methyl ester, (S)- (C_18_H_36_O_4_) | 18.685 | 0.08 | 167795 | Ester |
| 20. | (Z)6,(Z)9-Pentadecadien-1-ol (C_15_H_28_O) | 19.066 | 11.8 | 5365570 | Alcohol |
| 21. | Linoleic acid ethyl ester (C_20_H_36_O_2_) | 19.258 | 1.95 | 5282184 | Ester |
| 22. | 9,12,15-Octadecatrienoic acid, methyl ester, (Z,Z,Z)- (C_19_H_32_O_2_) | 19.361 | 3.84 | 5319706 | Ester |
| 23. | Tetradecanamide (C_14_H_29_NO) | 19.655 | 0.87 | 69492 | Amide |
| 24. | Dotriacontyl isopropyl ether (C_35_H_72_O) | 20.025 | 0.3 | 91692940 | Ether |
| 25. | Heneicosane (C_21_H_44_) | 21.529 | 0.55 | 12403 | Alkane |
| 26. | 1-Hydroxy-2,2,6,6-tetramethyl-3-piperidinomethyl-4-piperidone (C_15_H_28_N_2_O_2_) | 21.775 | 0.32 | 566506 | - |
| 27. | Dodecane, 5-cyclohexyl- (C_18_H_36_) | 22.256 | 0.14 | 524422 | Alkane |
| 28. | 9-Octadecenamide, (Z)- (C_18_H_35_NO) | 22.58 | 3.93 | 5283387 | Amide |
| 29. | Citronellol epoxide (R or S) (C_10_H_20_O_2_) | 23.005 | 0.07 | 98467 | - |
| 30. | 2-methyloctacosane (C_29_H_60_) | 23.252 | 0.32 | 519147 | Alkane |
| 31. | 9,12-Octadecadienoic acid (Z,Z)-, 2-hydroxy-1-(hydroxymethyl)ethyl ester (C_21_H_38_O_4_) | 23.375 | 0.25 | 5365676 | Fatty acid ester. |
| 32. | i-Propyl 9,12,15-octadecatrienoate (C_21_H_36_O_2_) | 23.485 | 0.57 | 14947687 | Ester |
| 33. | Oxalic acid, cyclohexylmethyl tridecyl ester (C_22_H_40_O_4_) | 23.935 | 0.1 | 6421725 | Ester |
| 34. | 1-Decanol, 2-octyl- (C_18_H_38_O) | 24.344 | 0.38 | 3084890 | Ester |
| 35. | Tetrapentacontane, 1,54-dibromo- (C_54_H_108_Br_2_) | 24.455 | 0.4 | 545963 | Fatty acid |
| 36. | Oleic Acid, (Z)-, TMS derivative (C_21_H_42_O_2_Si) | 24.625 | 0.75 | 5366433 | Alkene |
| 37. | Nonacosane (C_29_H_60_) | 24.747 | 1.3 | 12409 | Alkane |
| 38. | 2-Methylhexacosane (C_27_H_56_) | 24.837 | 0.4 | 150931 | Alkane |
| 39. | Hexadecanoic acid, 2-hydroxy-1-(hydroxymethyl)ethyl ester (C_19_H_38_O_4_) | 24.959 | 0.39 | 123409 | Ester |
| 40. | Tert-Hexadecanethiol (C_48_H_99_AuS_3_) | 25.085 | 0.09 | 109858 | - |
| 41. | 1-Nonadecene (C_19_H_38_) | 27.379 | 0.18 | 29075 | Alkene |
| 42. | Tetratetracontane (C_44_H_90_) | 27.825 | 4.26 | 23494 | Alkane |
| 43. | 1,1,3,6-tetramethyl-2-(3,6,10,13,14-pentamethyl-3-ethyl-pentadecyl)cyclohexane (C_32_H_64_) | 28.704 | 0.29 | 91693134 | - |
| 44. | 3-(6,6-Dimethyl-5-oxohept-2-enyl)-cyclohexanone (C_15_H_24_O_2_) | 29.405 | 0.14 | 5364977 | Ketone |
| 45. | 17-Pentatriacontene (C_35_H_70_) | 30.391 | 1.25 | 5365022 | Alkene |
| 46. | 9-Hexacosene (C_26_H_52_) | 30.505 | 1.09 | 5363630 | Alkene |
| 47. | .gamma.-Tocopherol (C_28_H_39_F_3_O_2_) | 32.816 | 0.11 | 92729 | Alcohol |
| 48. | Nonadecyl trifluoroacetate (C_21_H_39_O_2_) | 33.067 | 0.91 | 14574253 | Ester |
| 49. | Dotriacontane (C_32_H_66_) | 33.452 | 4.37 | 11008 | Alkane |
| 50. | Cholest-5-en-3-ol (3.beta.)-, propanoate (C_30_H_50_O_2_) | 33.901 | 1.05 | 313255 | Alcohol |
| 51. | Eicosyl isopropyl ether (C_23_H_48_O) | 34.907 | 0.47 | 91691499 | Ether |
| 52. | Tetracosamethyl-cyclododecasiloxane (C_24_H_72_O_12_Si_12_) | 35.145 | 0.56 | 167767 | Siloxane |
| 53. | Stigmasta-5,24(28)-dien-3-ol, (3.beta.)- (C_29_H_48_O) | 35.444 | 1.19 | 5281326 | Alcohol |
| 54. | Ergost-5-en-3-ol, (3.beta.)- (C_28_H_48_O) | 35.566 | 1.56 | 173183 | Alcohol |
| 55. | Stigmasterol (C_29_H_48_O) | 36.02 | 7.32 | 5280794 | Steroid |
| 56. | 1-Heptacosanol (C_27_H_56_O) | 36.13 | 2.74 | 74822 | Alcohol |
| 57. | Desmosterol (C_27_H_44_O) | 36.45 | 0.43 | 439577 | Sterol Alcohol |
| 58. | 1-Pentacosanol (C_25_H_52_O) | 36.795 | 0.27 | 92247 | Alcohol |
| 59. | .gamma.-Sitosterol (C_29_H_50_O) | 37.12 | 4.54 | 457801 | Alcohol |
| 60. | Cholest-5-en-3-ol, 24-propylidene-, (3.beta.)- (C_30_H_50_O) | 37.404 | 0.98 | 6443745 | Alcohol |
| 61. | Pregnan-17,21-diol-9,11-epoxy-3,20-dione, acetate (C_23_H_32_O_6_) | 37.614 | 0.84 | 540967 | Steroid |
| 62. | 9,19-Cyclolanostan-3-ol, acetate, (3.beta.)- (C_32_H_54_O_2_) | 37.846 | 0.57 | 537304 | Alcohol |
| 63. | Stigmast-7-en-3-ol, (3.beta.,5.alpha.,24S)- (C_29_H_50_O) | 38.264 | 0.1 | 5283639 | Alcohol |
| 64. | 4,22-Stigmastadiene-3-one (C_29_H_46_O) | 38.428 | 0.51 | 5364563 | Steroid |
| 65. | 9,19-Cyclolanost-24-en-3-ol, (3.beta.)- (C_30_H_50_O) | 38.72 | 0.17 | 92110 | Alcohol |
