# Supplementary material for: Unveiling the bactericidal effects of extracts and phytocompounds from Eichhornia crassipes (Mart.) Solms against methicillin-resistant Staphylococcus aureus (MRSA): An in vitro and in silico approach
Source: PLoS One. 2026 Jun 11;21(6):e0349750. doi: 10.1371/journal.pone.0349750 (PMC13258022; doi:10.1371/journal.pone.0349750)
Supplement: S5 Table — (DOCX) [file pone.0349750.s016.docx]

**S5 Table.** GC-MS identified phytochemicals in the methanol extract of *Eichhornia crassipes* leaves (MEECL).

| **Peak no.** | **Name and formula of the phytochemicals** | **Retention time** | **Area %** | **Compound CID** | **Nature of phytochemicals** |
| --- | --- | --- | --- | --- | --- |
| 1. | Ethyl iso-allocholate (C_26_H_44_O_5_) | 3.53 | 0.81 | 6452096 | Steroid derivative |
| 2. | 1,1,3,3-Tetramethyl-3-(1-methylpropoxy)disiloxan-1-ol (C_8_H_22_O_3_Si_2_) | 3.996 | 0.17 | 91724013 | Organosilicon alcohol |
| 3. | 1-Butanol, 3-methyl-, formate (C_6_H_12_O_2_) | 6.757 | 0.73 | 8052 | Fatty Ester |
| 4. | **Dodecane, 2,6,10-trimethyl- (C_15_H_32_)** | 8.492 | 0.29 | 19773 | Alkene, Sesquiterpene |
| 5.  . | 3-[N'-(3H-Indol-3-ylmethylene)-hydrazino]-5-methyl-[1,2,4]triazol-4-ylamine (C_12_H_13_N_7_) | 9.823 | 0.23 | 9600489 | Heterocyclic compound |
| 6. | 1,3-Propanediol, 2-(hydroxymethyl)-2-nitro- (C_4_H_9_NO_5_) | 10.193 | 5.25 | 31337 | Nitro alcohol |
| 7. | Pregnane-3,20-diol, (3.alpha.,5.beta.,20S)-, 2TMS derivative (C_27_H_52_O_2_Si_2_) | 10.365 | 0.82 | 22211864 | Steroid |
| 8. | Methyl(trimethylene)silyloxyoctane (C_12_H_26_OSi) | 10.534 | 0.65 | 588574 | Organosilicon |
| 9. | Phenol, 3,5-bis(1,1-dimethylethyl)- (C_14_H_22_O) | 10.778 | 0.48 | 70825 | Phenol |
| 10. | Fumaric acid, ethyl 2-propylphenyl ester (C_15_H_18_O_4_) | 11.39 | 0.4 | 91712905 | Ester |
| 11. | .alpha.-Methyl-l-sorboside (C_7_H_14_O_6_) | 11.505 | 0.3 | 219886 | Sorbose |
| 12. | Heptasiloxane, 1,1,3,3,5,5,7,7,9,9,11,11,13,13-tetradecamethyl- (C_14_H_42_O_6_Si_7_) | 11.585 | 0.21 | 6329088 | Siloxane |
| 13. | .alpha.-D-Glucopyranose, 4-O-.beta.-D-galactopyranosyl- (C_12_H_22_O_11_) | 11.881 | 0.98 | 84571 | Disaccharide |
| 14. | 4-Quinolinol, 6-fluoro- (C_9_H_6_FNO) | 11.915 | 0.96 | 2774498 | Quinoline |
| 15. | Fumaric acid, tetradecyl propyl ester (C_21_H_38_O_4_) | 12.065 | 0.63 | 91709943 | Ester |
| 16. | 1-Phenyl-7-methyl-5,6(4H)-dihydroimidazo(1,5-b)(1,2,4)triazepine-5-thione (C_13_H_12_N_4_S) | 12.145 | 0.79 | 5362658 | Heterocyclic compound |
| 17. | 2-Piperidinone, N-[4-bromo-n-butyl]- (C_9_H_16_BrNO) | 12.614 | 0.23 | 536377 | Ornithine Alkaloids |
| 18. | 6-Hydroxy-4,4,7a-trimethyl-5,6,7,7a-tetrahydrobenzofuran-2(4H)-one (C_11_H_16_O_3_) | 13.755 | 0.38 | 14334 | Terpenoids |
| 19. | Neophytadiene (C_20_H_38_) | 14.31 | 2.99 | 10446 | Terpenoids |
| 20. | 2,2,5-Trimethyl-cyclohexane-1,4-diol (C_9_H_18_O_2_) | 14.41 | 0.22 | 587708 | Terpenoids |
| 21. | 3,7,11,15-Tetramethyl-2-hexadecen-1-ol (C_20_H_40_O) | 14.909 | 0.96 | 5366244 | Alcohol |
| 22. | Hexadecanoic acid, methyl ester (C_17_H_34_O_2_) | 15.568 | 0.81 | 8181 | Ester |
| 23. | Hexasiloxane, tetradecamethyl- (C_14_H_42_O_5_Si_6_) | 17.505 | 0.2 | 7875 | Siloxane |
| 24. | cis-11,14-Eicosadienoic acid, methyl ester (C_21_H_38_O_2_) | 18.191 | 0.36 | 6430995 | Ester |
| 25. | 9,12,15-Octadecatrienoic acid, 2,3-dihydroxypropyl ester, (Z,Z,Z)- (C_21_H_36_O_4_) | 18.286 | 0.53 | 5367328 | Fatty acids |
| 26. | Phytol (C_20_H_40_O) | 18.44 | 1.14 | 5280435 | Terpenoids |
| 27. | l-Norvaline, N-(2-methoxyethoxycarbonyl)-, hexyl ester (C_15_H_29_NO5) | 18.809 | 0.66 | 91726564 | - |
| 28. | Tetradecanamide (C_14_H_29_NO) | 19.65 | 0.77 | 69492 | Fatty Amide |
| 29. | Carbonic acid, decyl tetradecyl ester (C_25_H_50_O_3_) | 19.881 | 0.33 | 91693142 | Ester |
| 30. | Fumaric acid, 2-dimethylaminoethyl octadecyl ester (C_26_H_49_NO_4_) | 21.253 | 0.25 | 91693823 | Fatty acids |
| 31. | 2-Methyltetracosane (C_25_H_52_) | 21.523 | 0.28 | 527459 | Fatty acids |
| 32. | Hexadecanoic acid, (3-bromoprop-2-ynyl) ester (C_19_H_33_BrO_2_) | 22.1 | 0.21 | 537302 | Ester |
| 33. | Trimethylsilyl 3-methyl-4-[(trimethylsilyl)oxy]benzoate (C_14_H_24_O_3_Si_2_) | 22.209 | 0.33 | 91740684 | Ester |
| 34. | 9-Octadecenamide, (Z)- (C_18_H_35_NO) | 22.56 | 30.02 | 5283387 | Fatty amide |
| 35. | Tetradecane, 2,6,10-trimethyl- (C_17_H_36_) | 23.24 | 0.21 | 85785 | Fatty acids |
| 36. | 2,3-Dioxabicyclo[2.2.2]oct-7-en-5-one, 1-(3-oxo-1-butenyl)-6,6,7-trimethyl (C_13_H_16_O_4_) | 23.665 | 0.19 | 5363634 | Lactone |
| 37. | **Cyclohexanepropionic acid, 4-oxo-, ethyl ester (C_11_H_18_O_3_)** | 24.605 | 0.24 | 143533 | Ester |
| 38. | 1-Bromo-4-bromomethyldecane (C_11_H_22_Br_2_) | 24.745 | 0.17 | 536423 | Alkane |
| 39. | Sulfurous acid, octadecyl 2-propyl ester (C_21_H_44_O_3_S) | 24.829 | 0.28 | 6420358 | Fatty acids |
| 40. | Hexadecanoic acid, 2-hydroxy-1-(hydroxymethyl)ethyl ester (C_19_H_38_O_4_) | 24.991 | 0.54 | 123409 | Fatty acids |
| 41. | DEHP (C_24_H_38_O_4_) | 25.243 | 0.42 | 8343 | Ester |
| 42. | trans-Geranylgeraniol (C_20_H_34_O) | 26.151 | 1 | 5281365 | Terpenoids |
| 43. | Fenpropathrin (C_22_H_23_NO_3_) | 27.086 | 0.17 | 47326 | Ester |
| 44. | i-Propyl 5,9,19-octacosatrienoate (C_31_H_56_O_2_) | 27.775 | 0.37 | 91697621 | Ester |
| 45. | 1-(Cyclopropyl-nitro-methyl)-cyclopentanol (C_9_H_15_NO_3_) | 28.355 | 0.24 | 534647 | Alcohol |
| 46. | Vinyl decanoate (C_12_H_22_O_2_) | 28.88 | 0.29 | 62140 | Fatty ester |
| 47. | 3-Hexen-1-ol, propanoate, (Z)- (C_9_H_16_O_2_) | 29.17 | 0.18 | 5365049 | Fatty ester |
| 48. | 3,3a-Epoxydicyclopenta[a,d]cyclooctan-4.beta.-ol, 9,10a-dimethyl-6-methylene-3.beta.-isopropyl- (C_20_H_32_O_2_) | 29.31 | 0.33 | 536470 | Terpenoids |
| 49. | Succinic acid, 2-methylpent-3-yl 2-methoxyphenyl ester (C_17_H_24_O_5_) | 29.45 | 1.04 | 91712382 | Ester |
| 50. | 4,7-Diisopropenyl-2,9-dimethyldecan-3,8-diol (C_18_H_34_O_2_) | 29.535 | 0.45 | 557576 | Alcohol |
| 51. | 1-(3-Hydroxypropyl)-5,5,8a-trimethyldecahydronaphthalen-2-ol (C_16_H_30_O_2_) | 29.585 | 0.44 | 535298 | Alcohol |
| 52. | Trimethyl[2-(tetramethyl-1,3,2-dioxaborolan-2-yl)ethynyl]silane (C_11_H_21_BO_2_Si) | 29.605 | 0.27 | 11447422 | Organosilicon |
| 53. | 2H-Tetrazole, 5-(thiophen-2-yl)- (C_5_H_4_N_4_S) | 29.655 | 0.27 | 235702 | Heterocyclic compound |
| 54. | Sambucinol (C_15_H_22_O_4_) | 29.685 | 0.44 | 5459101 | Phenol |
| 55. | 3-Chloropropionic acid, 2,6-dimethylnon-1-en-3-yn-5-yl ester (C_14_H_21_ClO_2_) | 29.749 | 0.24 | 531023 | Ester |
| 56. | Mandelic acid, 2TBDMS derivative (C_20_H_36_O_3_Si_2_) | 31.329 | 0.3 | 528583 | Aromatic acid |
| 57. | 2,3-Dihydroxypropyl icosanoate, 2TMS derivative (C_29_H_62_O_4_Si_2_) | 32.268 | 0.16 | 537899 | Ester |
| 58. | [Propanoic acid,2-methyl-,[(3aS,6R,6aR,9aS,9bR)-dodecahydro-6a-hydroxy-9a-methyl-3-methylene-2,9-dioxoazuleno[4,5-b]furan-6-yl]- methyl ester](https://pubchem.ncbi.nlm.nih.gov/compound/615944) (C_19_H_26_O_6_) | 32.355 | 0.26 | 615944 | Ester |
| 59. | 1-Dimethyl(3-chloropropyl)silyloxyoctadecane (C_23_H_49_ClOSi) | 32.71 | 0.15 | 554934 | Organosilicon |
| 60. | Cholesterol (C_27_H_46_O) | 33.882 | 1.88 | 5997 | Steroid |
| 61. | Isolongifolan-8-ol (C_15_H_26_O) | 34.565 | 0.32 | 535389 | Alcohol |
| 62. | 1-(.beta.-d-Arabinofuranosyl)-4-difluoromethyl-5-bromouracil (C_10_H_11_BrF_2_N_2_O_6_) | 34.6 | 1.11 | 13989305 | - |
| 63. | 1-Cyclododecanone, 2-ethylidene (C_14_H_24_O) | 35.185 | 0.56 | 5375859 | Ketone |
| 64. | 2,5-cyclohexadien-1-one, 4-[[4-(diethylamino)-2-methylphenyl]imino]-2-methyl-6-[[methyl(4-nitrophenyl)amino]methyl]- (C_26_H_30_N_4_O_3_) | 35.217 | 1.61 | 91741398 | - |
| 65. | Phosphonoacetic Acid, 3TMS derivative (C_11_H_29_O_5_PSi_3_) | 35.445 | 0.62 | 631032 | Inorganic acid |
| 66. | Ergost-5-en-3-ol, (3.beta.)- (C_28_H_48_O) | 35.535 | 1.49 | 173183 | Steroid |
| 67. | Stigmasterol (C_29_H_48_O) | 35.978 | 11.37 | 5280794 | Steroid |
| 68. | .beta.-Sitosterol (C_29_H_50_O) | 37.079 | 2.29 | 222284 | Steroid |
| 69. | Norgestrel, trimethylsilyl ether (C_24_H_36_O_2_Si) | 37.35 | 0.15 | 13368654 | Ether |
| 70. | .psi.,.psi.-Carotene, 1,1',2,2'-tetrahydro-1,1'-dimethoxy- (C_42_H_64_O_2_) | 37.387 | 0.45 | 5366411 | Terpenoid |
| 71. | Triisobutyl(2-phenylethoxy)silane (C_20_H_36_OSi) | 37.856 | 0.22 | 621863 | Organosilicon |
| 72. | Isocalamenediol (C_15_H_26_O_2_) | 38.2 | 0.15 | 91747826 | Alcohol |
