# Supplementary material for: Unveiling the bactericidal effects of extracts and phytocompounds from Eichhornia crassipes (Mart.) Solms against methicillin-resistant Staphylococcus aureus (MRSA): An in vitro and in silico approach
Source: PLoS One. 2026 Jun 11;21(6):e0349750. doi: 10.1371/journal.pone.0349750 (PMC13258022; doi:10.1371/journal.pone.0349750)
Supplement: S6 Table — (DOCX) [file pone.0349750.s017.docx]

**S6 Table.** GC-MS identified phytochemicals in the ethanol extract of *Eichhornia crassipes* leaves (EEECL).

| **Peak no.** | **Name and formula of the phytochemicals** | **Retention time** | **Area %** | **Compound CID** | **Nature of phytochemicals** |
| --- | --- | --- | --- | --- | --- |
| 1. | 1,6-Dideoxy-l-mannitol (C_6_H_14_O_4_) | 3.531 | 0.99 | [542304](https://pubchem.ncbi.nlm.nih.gov/compound/542304) | Alcohol |
| 2. | Butanoic acid, ethyl ester ([C_6_H_12_O_2_](https://pubchem.ncbi.nlm.nih.gov/#query=C6H12O2)) | **3.8** | **1.00** | [**7762**](https://pubchem.ncbi.nlm.nih.gov/compound/7762) | ester |
| 3. | 3,6,9,12,15-Pentaoxanonadecan-1-ol **(C_14_H_30_O_6_)** | **4.53** | **0.65** | **74516** | Alcohol |
| 4. | **Oxime-, methoxy-phenyl (C_8_H_9_NO_2_)** | 4.576 | 0.13 | 9602988 | - |
| 5. | Diphosphoric acid, diisooctyl ester (C_16_H_36_O_7_P_2_) | **5.342** | **0.59** | [**534663**](https://pubchem.ncbi.nlm.nih.gov/compound/534663) | ester |
| 6. | Mesitylene (C_9_H_12_) | 5.451 | 0.43 | 7947 | aromatic hydrocarbon |
| 7. | D-Limonene (C_10_H_16_) | 5.791 | 0.39 | 440917 | terpene |
| 8. | 1-Fluorononane (C_9_H_19_F) | **6.57** | **0.56** | **10029** | alkane |
| 9. | 1-Butanol, 3-methyl-, formate (C_6_H_12_O_2_) | 6.682 | 2.85 | 8052 | ester |
| 10. | 1-Decene (C_10_H_20_) | 7.618 | 0.41 | 13381 | ester |
| 11. | Exo-1,2-O-Ethylidene-.alpha.-d-erythrofuranose (C_6_H_10_O_4_) | 8.497 | 0.17 | 91691400 | - |
| 12. | Ethyl trans-2-decenoate (C_12_H_22_O_2_) | 9.61 | 0.20 | 5463904 | ester |
| 13. | 5-Octadecene, (E)- (C_18_H_36_) | 9.67 | 0.30 | 5364598 | alkene |
| 14. | Sucrose (C_12_H_22_O_11_) | 10.199 | 1.07 | 5988 | carbohydrate |
| 15. | 2-Oxooxolan-3-ylidene]amino}thiourea(C_5_H_7_N_3_O_2_S) | 10.43 | 0.23 | 45490185 | - |
| 16. | 9,12-Octadecadienoic acid (Z,Z)-, TMS derivative (C_21_H_40_O_2_Si) | 11.175 | 0.13 | 5352430 | - |
| 17. | Tetraethyleneglycol monomethylether (C_9_H_20_O_5_) | 11.236 | 1.55 | 90263 | ether |
| 18. | Fumaric acid, ethyl 2-methylallyl ester (C_10_H_14_O_4_) | 11.369 | 0.68 | 5461492 | ester |
| 19. | 3H-3,10a-Methano-1,2-benzodioxocin-3-ol, octahydro-7,7-dimethyl-, (3.alpha.,6a.beta.,10a.beta.)- (C_13_H_22_O_3_) | 11.475 | 0.72 | 606210 | - |
| 20. | 1-Nonadecene (C19H38) | 11.518 | 0.44 | 29075 | alcohol |
| 21. | 1-Cyclohexene-1-butanal, .alpha.,2,6,6-tetramethyl (C_14_H_24_O) | 11.816 | 0.17 | 579157 | aldehyde |
| 22. | Alpha.-campholenal (C_10_H_16_O) | 12.165 | 0.33 | 98497 | aldehyde |
| 23. | Cyclohexene, 1,5,5-trimethyl-6-acetylmethyl (C_12_H_20_O) | 12.61 | 0.34 | 579163 | alkene |
| 24. | 6-Hydroxy-4,4,7a-trimethyl-5,6,7,7a-tetrahydrobenzofuran-2(4H)-one (C_11_H_16_O_3_) | 13.743 | 0.70 | 14334 | alcohol |
| 25. | Neophytadiene (C_20_H_38_) | 14.313 | 4.24 | 10446 | diterpene |
| 26. | Ethene, (2-ethoxy-1-methoxyethoxy) (C_7_H_14_O_3_) | 14.408 | 1.58 | 547801 | alkene |
| 27. | 3,7,11,15-Tetramethyl-2-hexadecen 1-ol (C_20_H_40_O) | 14.912 | 1.40 | 5366244 | alcohol |
| 28. | Cyclopentanetridecanoic acid, methyl ester (C_19_H_36_O_2_) | 15.557 | 0.36 | 554135 | ester |
| 29. | Hexadecenoic acid, Z-11 (C_16_H_30_O_2_) | 15.937 | 1.70 | 5312414 | fatty acid |
| 30. | n-Hexadecanoic acid (C_16_H_32_O_2_) | 16.09 | 6.54 | 985 |  |
| 31. | Hexadecanoic acid, ethyl ester (C_18_H_36_O_2_) | 16.575 | 0.36 | 12366 | fatty acid ester |
| 32. | cis-11,14-Eicosadienoic acid, methyl ester (C_21_H_38_O_2_) | 18.183 | 0.25 | 6430995 | fatty acid ester |
| 33. | 8,11,14-Eicosatrienoic acid, methyl ester, (Z,Z,Z) (C_21_H_36_O_2_) | 18.281 | 0.28 | 5363092 | Ester |
| 34. | Phytol (C_20_H_40_O) | 18.439 | 2.02 | 5280435 | Terpenoids |
| 35. | Linoelaidic acid (C_18_H_32_O_2_) | 18.813 | 1.04 | 5282457 | fatty acid |
| 36. | 7-Tetradecenal, (Z) (C_14_H_26_O) | 18.91 | 0.72 | 5364468 | fatty aldehyde |
| 37. | **5-Keto-9-tetradecenamide (C_14_H_25_NO_2_)** | 18.98 | 0.33 | 5370333 | Fatty acid |
| 38. | Octadecanamide (C_18_H_37_NO) | 19.623 | 0.60 | 31292 | Fatty acid |
| 39. | Carbamic acid, 2-(dimethylamino)ethyl ester (C_5_H_12_N_2_O_2_) | 21.253 | 0.25 | 48131 | Ester |
| 40. | 9-Octadecenamide, (Z) (C_18_H_35_NO) | 22.555 | 25.21 | 5283387 | Fatty acid amide |
| 41. | Tetradecane, 2,6,10-trimethyl- (C_17_H_36_) | 23.239 | 0.34 | 85785 | Alkane |
| 42. | 3-Azonia-5-hexene-1-ol, N,N-dimethyl-, carbamate ester, bromide (C_8_H_17_N_2_O_2_^+^) | 24.115 | 0.13 | 535562 | Ester |
| 43. | 2-Isopropyl-5-methyl-1-heptanol (C_11_H_24_O) | 24.845 | 0.25 | 545941 | Alcohol |
| 44. | Hexadecanoic acid, 2-hydroxy-1-(hydroxymethyl)ethyl ester (C_19_H_38_O_4_) | 24.951 | 2.31 | 123409 | Ester |
| 45. | Ergost-25-ene-3,6-dione, 5,12-dihydroxy-, (5.alpha.,12.beta.)- (C_28_H_44_O_4_) | 25.04 | 0.26 | 91692405 | Steroid |
| 46. | 1,3-Dioxan-4-one, 2-(1,1-dimethylethyl)-6-methyl-5-methylene-, cis-(.+-.)- (C_10_H_16_O_3_) | 25.09 | 0.35 | 14739241 | Ketone |
| 47. | 1,6,10,14,18,22-Tetracosahexaen-3-ol, 2,6,10,15,19,23-hexamethyl-, (all-E)-(.+/-.)- (C_30_H_50_O) | 26.157 | 0.85 | 5366014 | Carotenoid |
| 48. | Z,E-7,11-Hexadecadien-1-yl acetate (C_18_H_32_O_2_) | 27.7 | 0.75 | 5363282 | Fatty acid ester |
| 49. | Fumaric acid, 2-methylallyl nonyl ester (C_17_H28O4) | 27.813 | 0.27 | 91694763 | Ester |
| 50. | 1,1,6-trimethyl-3-methylene-2-(3,6,10,13,14-pentamethyl-3-ethenyl-pentadec-4-enye)cyclohexane (C_32_H_58_) | 27.88 | 0.16 | 91697687 | Terpenoid |
| 51. | 1-(2-Isopropyl-5-methylcyclopentyl)ethanone (C_11_H_20_O) | 28.115 | 0.15 | 537646 | Ketone |
| 52. | Ethyl geranyl acetate (C_14_H_24_O_2_) | 28.3 | 0.15 | 5363297 | Ester |
| 53. | (Z)-5-(Pentadec-8-en-1-yl)benzene-1,3-diol (C_21_H_34_O_2_) | 29.344 | 8.09 | 5281852 | Alkyl benzene |
| 54. | Cyclohexane, 1,1'-(1-methylethylidene)bis- (C_19_H_32_O_2_) | 29.53 | 0.33 | 11833232 | Cyclohexane |
| 55. | 1-Bromo-4-bromomethyldecane (C_11_H_22_Br_2_) | 29.625 | 0.46 | 536423 | Alkane |
| 56. | (E)-Dodec-2-enyl ethyl carbonate (C_15_H_28_O_3_) | 30.706 | 0.31 | 91697792 | Ester |
| 57. | Retinal (C_20_H_28_O) | 32.349 | 0.16 | 638015 | Vitamin A |
| 58. | Cholesterol (C_27_H_46_O) | 33.884 | 2.18 | 5997 | Steroid |
| 59. | Butyl 9-decenoate (C_27_H_46_O) | 35.439 | 0.13 | 17825102 | Fatty acid esters |
| 60. | Ergost-5-en-3-ol, (3.beta.) (C_28_H_48_O) | 35.546 | 1.11 | 5283637 | Steroid |
| 61. | Stigmasterol (C_29_H_48_O) | 35.988 | 16.71 | 5280794 | Steroid |
| 62. | Beta-Sitosterol (C_29_H_50_O) | 37.081 | 2.11 | 222284 | Steroid |
| 63. | Cholest-5-en-3-ol, 24-propylidene-, (3.beta.) (C_30_H_50_O) | 37.379 | 0.55 | 6443745 | Sterols |
