# Supplementary material for: Unveiling the bactericidal effects of extracts and phytocompounds from Eichhornia crassipes (Mart.) Solms against methicillin-resistant Staphylococcus aureus (MRSA): An in vitro and in silico approach
Source: PLoS One. 2026 Jun 11;21(6):e0349750. doi: 10.1371/journal.pone.0349750 (PMC13258022; doi:10.1371/journal.pone.0349750)
Supplement: S7 Table — (DOCX) [file pone.0349750.s018.docx]

**S7 Table.** GC-MS identified phytochemicals in the ethyl acetate extract of *Eichhornia crassipes* leaves (EAEECL).

| **Peak no.** | **Name and formula of the phytochemicals** | **Retention time** | **Area %** | **Compound CID** | **Nature of phytochemicals** |
| --- | --- | --- | --- | --- | --- |
| 1. | Decane, 3,7-dimethyl- (C_12_H_26_) | 6.091 | 0.21 | 28468 | Alkane |
| 2. | 9-Octadecene, (E)- (C_18_H_36_) | 9.669 | 2.01 | 5364599 | Alcohol |
| 3. | Tetracosane, 1-iodo- (C_24_H_49_I) | 10.565 | 0.19 | 11282694 | Alkane |
| 4. | **Neophytadiene (C_20_H_38_)** | 14.316 | 1.79 | 10446 | Diterpene |
| 5. | 3,7,11,15-Tetramethyl-2-hexadecen-1-ol (C_20_H_40_O) | 14.914 | 0.50 | 5366244 | Alcohol |
| 6. | Octasiloxane, 1,1,3,3,5,5,7,7,9,9,11,11,13,13,15,15-hexadecamethyl- (C_16_H_48_O_7_Si_8_) | 15.415 | 2.69 | 6329087 | Siloxane |
| 7. | Heptacos-1-ene (C_27_H_54_) | 16.591 | 1.18 | 528971 | Alkene |
| 8. | 9-Octadecenamide, (Z)- (C_18_H_35_NO) | 22.561 | 23.95 | 5283387 | Amide |
| 9. | 11-Methyltricosane (C_24_H_50_) | 22.69 | 1.51 | 530326 | Alkane |
| 10. | Triacontyl heptafluorobutyrate (C_34_H_61_F_7_O_2_) | 23.08 | 1.75 | 91693301 | Ester |
| 11. | Pentatriacontane (C_35_H_72_) | 23.258 | 1.06 | 12413 | Alkane |
| 12. | Eicosyl isopropyl ether (C_23_H_48_O) | 23.797 | 3.00 | 91691499 | Ether |
| 13. | Carbonic acid, eicosyl vinyl ester (C_23_H_44_O_3_) | 24.593 | 1.06 | 91693137 |  |
| 14. | Tetratetracontane (C_44_H_90_) | 24.843 | 1.65 | 23494 | Alkane. |
| 15. | Octatriacontyl trifluoroacetate (C_40_H_77_F_3_O_2_) | 25.085 | 0.40 | 91693163 | Ester |
| 16. | Cyclohexane, 1,4-didecyl- (C_26_H_52_) | 25.57 | 0.26 | 296270 | Cyclohexane |
| 17. | 6,6-Diethylhoctadecane (C_22_H_46_) | 25.6 | 1.28 | 85977288 | Alkane |
| 18. | 1-Pentacosanol (C_25_H_52_O) | 25.75 | 1.56 | 92247 | Fatty alcohol |
| 19. | Tetrapentacontane (C_54_H_110_) | 26.12 | 1.77 | 521846 | Alkane |
| 20. | Tetrapentacontane, 1,54-dibromo- (C_54_H_108_Br_2_) | 26.359 | 2.93 | 545963 | Alkane |
| 21. | [1,1'-Bicyclohexyl]-4-carboxylic acid, 4'-pentyl-, 4-fluorophenyl ester (C_24_H_35_FO_2_) | 26.511 | 1.25 | 557929 | Ester |
| 22. | Carbonic acid, but-2-yn-1-yl heptadecyl ester (C_22_H_40_O_3_) | 26.628 | 0.40 | 91693224 | Ester |
| 23. | Hexacontane (C_60_H_122_) | 26.862 | 1.82 | 24318 | Alkane |
| 24. | 7-Hexadecenal, (Z)- (C_16_H_30_O) | 26.975 | 0.78 | 5364438 | Aldehyde |
| 25. | (1S,4R,5R)-1,3,3-Trimethyl-2-oxabicyclo[2.2.2]octan-5-ol (C_10_H_18_O_2_) | 27.02 | 0.21 | 439906 | Alcohol |
| 26. | Dodecane, 6-cyclohexyl- (C_18_H_36_) | 27.308 | 0.26 | 524434 | Alkane |
| 27. | 2-Octyldecyl propionate (C_21_H_42_O_2_) | 27.394 | 0.85 | 91693215 | Ester |
| 28. | Carbonic acid, octadecyl vinyl ester (C_21_H_40_O_3_) | 27.642 | 0.54 | 91693138 | Carbonic acid & Ester |
| 29. | Dotriacontane (C_32_H_66_) | 27.875 | 1.72 | 11008 | Alkane |
| 30. | 1,4-Piperazinediethanol, .alpha.,.alpha.'-bis(phenoxymethyl)- (C_22_H_30_N_2_O_4_) | 28.459 | 0.33 | 3095130 | - |
| 31. | E-10,13,13-Trimethyl-11-tetradecen-1-ol acetate (C_19_H_36_O_2_) | 28.535 | 0.21 | 5365074 | Alcohol |
| 32. | Disparlure (C_19_H_38_O) | 28.57 | 0.59 | 205983 | Pheromone |
| 33. | Disulfide, di-tert-dodecyl (C_24_H_50_S_2_) | 28.804 | 0.94 | 117981 | - |
| 34. | Octatriacontyl pentafluoropropionate (C_41_H_77_F_5_O_2_) | 29.32 | 0.71 | 91693082 | - |
| 35. | Fumaric acid, 4-methoxyphenyl 8-chlorooctyl ester (C_19_H_25_ClO_5_) | 29.369 | 2.65 | 91712129 | Ester |
| 36. | Pentadec-7-ene, 7-bromomethyl- (C_16_H_31_Br) | 30.105 | 0.24 | 5362747 | - |
| 37. | **Hexadecanal (C_16_H_32_O)** | 30.52 | 1.02 | 984 | Aldehyde |
| 38. | Heptyl triacontyl ether (C_37_H_76_O) | 30.6 | 1.54 | 91692961 | Ether |
| 39. | 1-Hexacosene (C_26_H_52_) | 30.955 | 0.50 | 29303 | Alkene |
| 40. | Hexacosyl nonyl ether (C_35_H_72_O) | 31.175 | 0.68 | 91693158 | Ether |
| 41. | 1,1,3,6-tetramethyl-2-(3,6,10,13,14-pentamethyl-3-ethyl-pentadecyl)cyclohexane (C_32_H_64_) | 31.53 | 0.28 | 91693134 | - |
| 42. | 1-Decanol, 2-hexyl- (C_16_H_3_4O) | 32.055 | 0.19 | 95337 | Alcohol |
| 43. | Isocholesteryl methyl ether (C_28_H_48_O) | 33.895 | 1.68 | 262105 | Ether |
| 44. | Stigmasterol (C_29_H_48_O) | 35.996 | 29.85 | 5280794 | sterol |
