# Supplementary material for: Unveiling the bactericidal effects of extracts and phytocompounds from Eichhornia crassipes (Mart.) Solms against methicillin-resistant Staphylococcus aureus (MRSA): An in vitro and in silico approach
Source: PLoS One. 2026 Jun 11;21(6):e0349750. doi: 10.1371/journal.pone.0349750 (PMC13258022; doi:10.1371/journal.pone.0349750)
Supplement: S8 Table — (DOCX) [file pone.0349750.s019.docx]

**S8 Table**. Protein ligand interaction analysis of β-lactamase and PBP2a proteins with CID 4970, CID 615944 and control CID 6087.

| **Targeted protein** | **Compound CID** | **Interacting Residues** | **Hydrogen Bonds** | **Hydrophobic Bonds** |
| --- | --- | --- | --- | --- |
| **β-lactamase** | **CID 4970** | ALA 69, SER 70, THR 71, LYS 73, ALA 104, TYR 105, TYR 129, SER 130, ASP 131, ASN 132, GLU 166, ILE 167, ASN 170, TYR 171, ASN 214, SER 216, LEU 220, LYS 234, SER 235, GLY 236, GLN 237, ALA 238, ILE 239, THR 240 | ASN 132 | ALA 69, ALA 104, TYR 105, TYR 129, ILE 167, TYR 171, LEU 220, ALA 238, ILE 239 |
|  | **CID 615944** | ALA 69, SER 70, THR 71, LYS 73, ALA 104, TYR 105, SER 126, THR 128, TYR 129, SER 130, ASP 131, ASN 132, GLU 166, ILE 167, LEU 169, ASN 170, ASN 214, LYS 215, SER 216, GLY 217, THR 219, LEU 220, LYS 234, SER 235, GLY 236, GLN 237, ALA 238, ILE 239, ARG 244, ASN 245, ASP 276 | ARG 244 | ALA 69, ALA 104, TYR 105, TYR 129, ILE 167, LEU 169, LEU 220, ALA 238, ILE 239 |
|  | **CID 6087 (Control)** | TYR 68, ALA 69, SER 70, THR 71, SER 72, LYS 73, VAL 103, ALA 104, TYR 105, SER 106, SER 126, MET 127, THR 128, TYR 129, SER 130, ASP 131, ASN 132, GLU 166, ILE 167, LEU 169, ASN 170, TYR 171, ASN 214, SER 216, GLY 217, LEU 220, LYS 234, SER 235, GLY 236, GLN 237, ALA 238, ILE 239, THR 240, SER 243, ARG 244, ASN 245 | SER 130, SER 235, GLN 237, ARG 244 | TYR 68, ALA 69, VAL 103, ALA 104, TYR 105, MET 127, TYR 129, ILE 167, LEU 169, TYR 171, LEU 220, ALA 238, ILE 239 |
| **PBP2a** | **CID 4970** | GLY 402, SER 403, LYS 406, THR 444, ARG 445, TYR 446, GLU 447, VAL 448, ILE 459, GLU 460, SER 461, SER 462, ASP 463, ASN 464, TYR 519, VAL 578, LYS 581, THR 582, HIS 583, LYS 584, GLU 585, ASP 586, LYS 597, SER 598, GLY 599, THR 600, ALA 601, TRP 616, PHE 617, GLY 640, MET 641, ALA 642, SER 643 | THR 600 | TYR 446, VAL 448, ILE 459, TYR 519, VAL 578, ALA 601, TRP 616, PHE 617, MET 641, ALA 642, SER 643 |
|  | **CID 615944** | ILE 171, GLU 189, LEU 190, SER 191, LYS 215, THR 216, VAL 217, LYS 218, LYS 219, ASP 221, GLU 222, TYP 223, LEU 224, SER 225, ASP 226, PHE 227, ALA 228, LYS 230, ASP 367, VAL 368, TYR 369, PRO 370, PHE 371, MET 372, TYR 373, GLY 374, MET 375, SER 376, GLU 379 | N/A | ILE 171, LEU 190, VAL 217, TYP 223, LEU 224, PHE 227, ALA 228, VAL 368, TYR 369, PRO 370, PHE 371, MET 372, TYR 373, MET 375 |
|  | **CID 6087 (Control)** | GLY 402, SER 403, THR 404, LYS 406, TYR 446, GLU 447, ILE 459, GLU 460, SER 461, SER 462, ASP 463, ASN 464, VAL 578, LYS 581, THR 582, HIS 583, LYS 584, GLU 585, ASP 586, LYS 597, SER 598, GLY 599, THR 600, ALA 601, ILE 614, TRP 616, PHE 617, LYS 639, GLY 640, MET 641, ALA 642, SER 643, TYR 644, ASN 645 | ALA 642 | TYR 446, ILE 459, VAL 578, ALA 601, ILE 614, TRP 616, PHE 617, MET 641, ALA 642, TYR 644 |
